# The expression of *Pax6* and retinal determination genes in the eyeless arachnid *A. longisetosus* reveals vestigial eye primordia

**DOI:** 10.1186/s13227-025-00245-7

**Published:** 2025-07-09

**Authors:** Isabella Joyce, Austen A. Barnett

**Affiliations:** Department of Biology, DeSales University, Center Valley, PA USA

## Abstract

**Background:**

Evidence suggests that *Pax6* genes are necessary for the specification of eyes in a variety of metazoans, including mandibulate arthropods. In these arthropods, *Pax6* genes usually interact with a conserved set of genes, collectively called the retinal determination gene network (RDGN), to specify eye cells. However, recent data have argued that *Pax6* genes lack a role in the development of the eyes in Chelicerata (= arachnids, horseshoe crabs, and sea spiders). A genome sequence of the eyeless mite *Archegozetes longisetosus* revealed that it retains two *Pax6* paralogs, as well as singleton orthologs of all RDGN genes. We hypothesized that the retention of these two *Pax6* paralogs could be due to their non-eye determining roles, and/or their expression in vestigial eye primordia. We therefore used hybridization chain reactions (HCRs) to follow the embryonic expression of these genes.

**Results:**

To provide a basis for understanding RDGN expression patterns, we developed a staging system for *A. longisetosus* head development. This showed the presence of structures that in other arachnids form neural components of all eye types. We then showed that two genes in the RDGN of eyed arachnids, *i.e*., *sine oculis* and *atonal*, are expressed in a manner that are suggestive of vestigial eye primordia. We also found that the expression of the *Pax6* paralogs was consistent with their roles in the development of the central nervous system. By co-staining for these genes with the conserved head-patterning gene *orthodenticle*, we observed early expression patterns of these genes in the brains of early *A. longisetosus* embryos that are comparable to those arachnids with embryonic eyes.

**Conclusions:**

Our data provide support for the hypothesis that the retention of *Pax6* genes in *A. longisetosus* is due to their non-eye patterning roles. Furthermore, our survey of RDGN gene expression also provides support that *A. longisetosus* patterns vestigial eye primordia. Lastly, our data suggest that the *Pax6* genes, with *orthodenticle*, acts to specify the ancestral arachnid brain. We then discuss our results considering eye loss in other arachnids.

**Supplementary Information:**

The online version contains supplementary material available at 10.1186/s13227-025-00245-7.

## Background

Eyes have likely evolved independently multiple times within animals, underscoring their adaptive advantage in a variety of ecosystems [1, 2]. These complex and diverse organs can be found across disparate metazoan lineages, including cnidarians, mollusks, vertebrates, and arthropods (reviewed in [3]). Ancestrally, the arthropods (= insects, crustaceans, myriapods and chelicerates) had a pair of multifaceted compound eyes, as well as multiple simple eyes called ocelli [4, 5]. This basic theme is generally conserved across arthropods, albeit with lineage-specific modifications [6, 7].

The euarthropod clade Chelicerata (= arachnids, horseshoe crabs, and sea spiders) comprises many species that have modified this basic theme to suit diverse ecological niches [8]. Evidence suggests that the arachnid lateral eyes are homologous to the insect compound eyes, whereas the medial eyes are homologous to the ocelli [6, 9, 10]. This ground plan differs from that of the likely sister group of arachnids, Xiphosura (= horseshoe crabs [11], but see [12, 13] for an alternate hypothesis). Instead, horseshoe crabs retain the basal arthropod state of housing both compound eyes and ocelli [4]. Sea spiders (= pycnogonids) also represent a non-arachnid chelicerate group, and these animals bear two pairs of median eyes, but lack lateral eyes [14, 15]. At the other extreme of chelicerate visual systems lie groups that have lost their eyes entirely, including many members of Acariformes, the clade comprised mites [16].

Despite the multiple occurrences of independent eye evolution across animal phyla, the development of these organs usually involves the utilization of Pax6 transcription factors [1]. Within arthropods, including chelicerates, phylogenetic evidence suggests that the last common ancestor of arthropods likely had at least two paralogous *Pax6* genes (see [17]), first identified as *eyeless* (*ey*) [18] and *twin of eyeless* (*toy*) [19] in the fruit fly *Drosophila melanogaster*. These distinct *Pax6* paralogs are used differentially across arthropod groups, obfuscating their ancestral roles in arthropod eye development. For example, in *D. melanogaster,* both *ey* and *toy* are expressed in the eye/antennal imaginal discs, which give rise to the adult eyes [20]*.* In these imaginal discs, both *ey* and *toy* have been shown to be necessary for the development of the compound eye [18], whereas only *toy* is necessary for specifying the ocelli [21–23]. In another insect, the beetle *Tribolium castaneum*, both *Pax6* orthologs are required for the formation of the adult eyes, however they do act redundantly in this role with a third transcription factor, Dachshund (Dac) [24]. In crustacean exemplars, the knockout of *ey* in *Daphnia magna* generated eye deformities [25]. In the decapod crustacean *Exopalaemon carinicauda*, the knockout of *ey* resulted in a range of compound eye deformities, whereas the knockout of *toy* had no effect on eye development [26]. Studies into the utilization of *Pax6* orthologs in myriapods are thus far restricted to expression studies on the millipede *Glomeris marginata*, where both *toy* and *ey* are expressed in the developing head [27], and on the centipede *Strigamia maritima*, where its *Pax6* orthologs (= *Pax-6A* and *Pax-6B*) are also both expressed in the developing head (however, note that *S. maritima* lacks eyes [28]). These studies are suggestive of a conserved role of *Pax6* orthologs in at least some processes of eye development in the mandibulates, *i.e.*, the clade comprised insects, crustaceans and myriapods.

The utilization of *Pax6* orthologs in developing chelicerate eyes, however, is much less clear, as initially demonstrated by studies on horseshoe crabs. The current data suggest that the last common ancestor of Xiphosura underwent three rounds of whole-genome duplications [29–31]. These expansions of the horseshoe crab genome resulted in the retention of three *ey* orthologs and two *toy* orthologs in their genome [31]. Prior to these findings, an expression study in the Atlantic horseshoe crab *Limulus polyphemus* showed that a *toy* ortholog was not expressed in any of the eye anlagen during embryogenesis [32]. This discovery was surprising, as it was the first to suggest that a *Pax6* ortholog does not contribute to eye development in a chelicerate species. This unexpected revelation was further complicated by studies into the utilization of *Pax6* orthologs in the likely sister clade to Xiphosura, the arachnids.

Within arachnids, the expression of *ey* and *toy* has been studied extensively in spiders. In the spider *Cupiennius salei*, *ey* (= *Cs-pax6a*) was expressed in the developing medial eyes. However, its *toy* ortholog (= *Cs-pax6b*) was only expressed in the anlagen of the optic neuropils that became associated with the medial eyes, and not the eyes themselves [9]. In the spider *Parasteatoda tepidariorum*, neither its *ey* (= *Pt-pax6.1*) nor its *toy* (= *Pt-pax6.2*) orthologs were expressed in any of the developing eyes but were rather expressed in the developing neural tissue that is adjacent to the anlagen of the anterior medial eyes [10, 33]. A recent survey of eye development genes across seven spider species also showed no evidence of *Pax6* gene input into the development of their eyes [34] and another recent study into the expression of conserved retinal determination genes in the cave spider *Tegenaria pagana* recovered no *Pax6* expression from their embryonic transcriptome, which the authors took to suggest that *Pax6* input is not required for its eye development [35]. Outside of spiders, *Pax6* expression was surveyed in the opilionid (daddy-longlegs) *Phalangium opilio*. *P. opilio Pax6* paralogs were found to be expressed in parts of the brain, as well as its eyes. Specifically, *Po-Pax6a* was expressed in the lentigenic layer of the eye at later embryonic stages, and both of its *Pax6* paralogs were mainly expressed in the neural tissue of the eye folds [36].

Aside from eye development, a distinct, and possibly independent, function for *Pax6* genes in arthropods may be uncovered by their expression in the non-eye patterning regions of the arthropod brain and head. The specific architecture of the arthropod brain is currently debated, and these debates hinge on whether the arthropod brain is segmented, non-segmented, or some combination thereof (see [37–40] and arguments therein). Nonetheless, the arthropod brain develops from the most anterior region of the embryo. This region, often called the “ocular region” (*e.g., *sensu [37]), is directly anterior to the deutocerebral segment, *i.e.*, the cheliceral segment in chelicerates and the first antennal segment of mandibulates. Furthermore, this region is characterized by anterior *six3* expression and more posterior expression of *orthodenticle* (see [37, 39, 40] for arguments and summaries of expression data). *Pax6* genes are also expressed in non-eye producing domains of the ocular region across arthropod taxa, including arachnids ( [9, 10, 34, 36]; see [37, 40] for summaries). There is also functional evidence that arthropod *Pax6* genes operate in the development of non-visual components of the brain. For example, in *T. castaneum*, both *ey* and *toy* are expressed in the ocular region, and function redundantly to specify the head lobes that subsequently develop into components of the brain [41]. Additionally, this study showed *Pax6* functions in the mushroom bodies. Furthermore, it was shown that *eyeless* mutants in *D. melanogaster* have severe brain defects [42]. Taken together, these data support the role of *Pax6* genes in ancestrally patterning both eye and non-eye-patterning precursors of the ocular region.

The megadiverse arachnid clade Acariformes comprises 54,614 nominal species of mites [43]. Eye loss is widespread among acariform mites; however, how extensive eye loss is in this clade is currently unknown, nor is it known how many convergent eye loss events have occurred in Acariformes (see [8, 16, 44, 45] for review). Eye loss is a common occurrence in endogean (*i.e*., soil-dwelling) species, and such species often show similar phenotypes to species inhabiting cave ecosystems (*i.e*., troglodytic species) [35, 46–52]. Numerous acariform species occupy endogean habitats, likely leading to the loss of their eyes [44, 45, 53]. The acariform mite *Archegozetes longisetosus* is one such soil-dwelling species that has secondarily lost its eyes (reviewed in [44, 54, 55]). Despite its loss of eyes, the recently published genome sequence of *A. longisetosus* paradoxically showed that it retains both *ey* and *toy* orthologs [54]. Furthermore, orthologs of genes that are commonly used in arthropod eye development, collectively called the Retinal Determination Gene Network (RDGN) were also present. Moreover, this study provided RNAseq evidence that the *Pax6* genes and the RDGN genes were expressed during embryogenesis [54]. These observations are indicative for a role in *Pax6* and other RDGN genes in some set of developmental roles in *A. longisetosus*.

Recent studies have focused on the developmental expression of RDGN genes in troglomorphic arachnids, [35, 56]. One study compared the transcriptome of a whip spider with reduced eyes to a closely related eye-bearing whip spider. The resulting data suggested that RDGN components, including *Pax6* genes, were likely targets of natural selection that have led to the loss of eyes [56]. To better understand how *Pax6* and other RDGN genes are expressed in arachnids that have lost their eyes, and to thus to reveal potential convergent developmental events leading to eye loss, we followed the embryonic expression of these genes in *A. longisetosus*. Initially, to gain a better understanding of the development of the mite ocular region and its subsequent derivatives, we described the morphogenesis of this region from the germ band stage through the early prelarval stage. This provided the first staging system of brain/head development in a mite species using modern confocal microscopy to date. It has also recently been shown in another arachnid exemplar that by observing the expression of RDGN components, vestigial visual organs can be identified [36]. To test the hypothesis that *A. longisetosus* lacks vestigial eye primordia, we followed the expression of the RDGN genes identified in [54] using Hybridization Chain Reactions (HCRs) and confocal microscopy. We found that two components of the RDGN, *i.e., Al-eyes absent* and *Al-sine oculis* are expressed in an ocular domain, in a manner like other arachnid taxa that have eyes. However, these expression patterns disappeared at later stages of development. These data are consistent with the hypothesis that *A. longisetosus* patterns some vestigial eye primordia during mid-embryogenesis, however the remaining downstream differentiation of eye structures is either inhibited or not activated. To explore the potential roles for the *Pax6* genes in this eyeless arachnid, we also followed their expression throughout embryonic development. We conclude that *ey* likely participates in the establishment of brain compartments, specifically the optic vesicles and the mushroom bodies of the mite protocerebrum. Furthermore, we show that the expression dynamics of *Al-toy* are consistent with a role in establishing the prosomal shield, a conserved arachnid structure that migrates late in development to cover the brain. We also followed the expression of the conserved head-patterning gene *orthodenticle* in *A. longisetosus* simultaneously with *Pax6* expression in early blastoderm stages. This provided further support for the role of *Pax6* genes in the development of the early *A. longisetosus* head/anterior region.

Taken together, our results provide the first embryonic expression patterns of *Pax6* and RDGN genes in an acariform mite. Additionally, our results provide a framework for understanding the development of arachnid central nervous systems and provide a basis for future comparative studies into the convergent loss of eyes seen in other cave-dwelling and soil-dwelling arachnid taxa.

## Methods

### Animal husbandry, embryo collection, and embryo fixation

Mites were reared on a plaster-of-Paris/charcoal substrate in plastic jars to maintain appropriate humidity. Mites were kept in these jars in an incubator at 25 °C with wood chips to promote oviposition. Mites were fed with brewer’s yeast daily. Mite embryos were collected and fixed in the same manner described in [57–60]. Our lab population was started as a gift by Dr. Adrian Bruckner from the California Technical Institute, which was started from the original population raised by Dr. Roy Norton.

### Gene identification and bioinformatic analyses

The *A. longisetosus* singleton orthologs of *wingless, peropsin, rhodopsin, eyes absent, Six3, sine oculis,* and *atonal* were identified previously in [54]. The ortholog of *orthodenticle* was identified in [61], and *dachshund* in [59]. To identify the potential *A. longisetosus* orthologs of *ey, toy, beta-arrestin,* and *myosin-III*, the *D. melanogaster* orthologs of each gene were used as queries for a tBLASTn screen of the *A. longisetosus* transcriptome [54]. The resulting top hits were transcribed and subsequently aligned with selected metazoan protein sequences using MUSCLE with eight iterations [62]. These alignments were then used with PhyML [63] and the Smart Model Selection (SMS) tool [64] to construct phylogenetic trees. Branch support for these trees were also calculated using the approximate likelihood-ratio test (SH-like) [65]. All trees were then edited to make publication-quality images using FigTree (v1.4.3). All phylogenetic statistics are reported in Table S1.

### Hybridization chain reactions and imaging

For all single and double hybridization chain reactions (HCRs), we followed the protocol developed by [66]. Probes specific to each mRNA were developed using the HCR 3.0 Probe Maker software [67]. The mRNA sequences used for probe production were from the transcriptome assembled in [54]. The identifiers of all transcripts used to design probes, as well as their associated HCR amplifiers, can be found in Table S2.

All resulting probes were ordered as oPools from Integrated DNA Technologies at a scale of 50 pmol per oligo. The probe sequences that were used in this study are listed in Tables S4–S17. If a transcript was too small to make the recommended 20 pairs of probes (*i.e., Al-arrestin-2* and *Al-peropsin*), we increased the probe concentration twofold as recommended by [66]. Control HCRs were performed in parallel but lacked the addition of DNA probes. All HCR buffers and HCR amplifiers were purchased from Molecular Instruments. The amplifier fluorophores were also ordered from Molecular Instruments, and included fluorophores 594, 514, and 647 for use with amplifiers B1, B2, and B3, respectively. All HCR imaging was done on a Zeiss LSM 880 at Lehigh University, Bethlehem, PA. All images were processed in FIJI (v.2.9.0/1.53t), and all figures were assembled using Adobe Illustrator CS6.

## Results

### Development and compartmentalization of the *A. longisetosus* brain

In an effort both to establish a basis for comparable gene expression patterns between *A. longisetosus* and other arachnids, and to determine if any vestiges of eye development are retained during *A. longisetosus* embryogenesis, we followed the embryonic development of the *A. longisetosus* brain. It is important to note that, unlike most emerging arthropod systems, *A. longisetosus* adults lay eggs at mixed developmental stages. This is because its oviducts serve as brood chambers, and therefore its clutches of eggs often contain embryos at different stages of development [68]. Consequently, the traditional “hours after egg laying” criterion cannot be used for this species. Instead, we use morphology to establish brain/head development-specific stages for the remainder of this paper*, *e.g., “Brain-Development Stage-1 (= BDS-1).” Also, because development is a continuum, we ascribe non-integer stages to intermediate stages in the remainder of the manuscript (e.g., BDS-3.5 is an intermediate of BDS-3 and BDS-4).

**Fig. 1:**
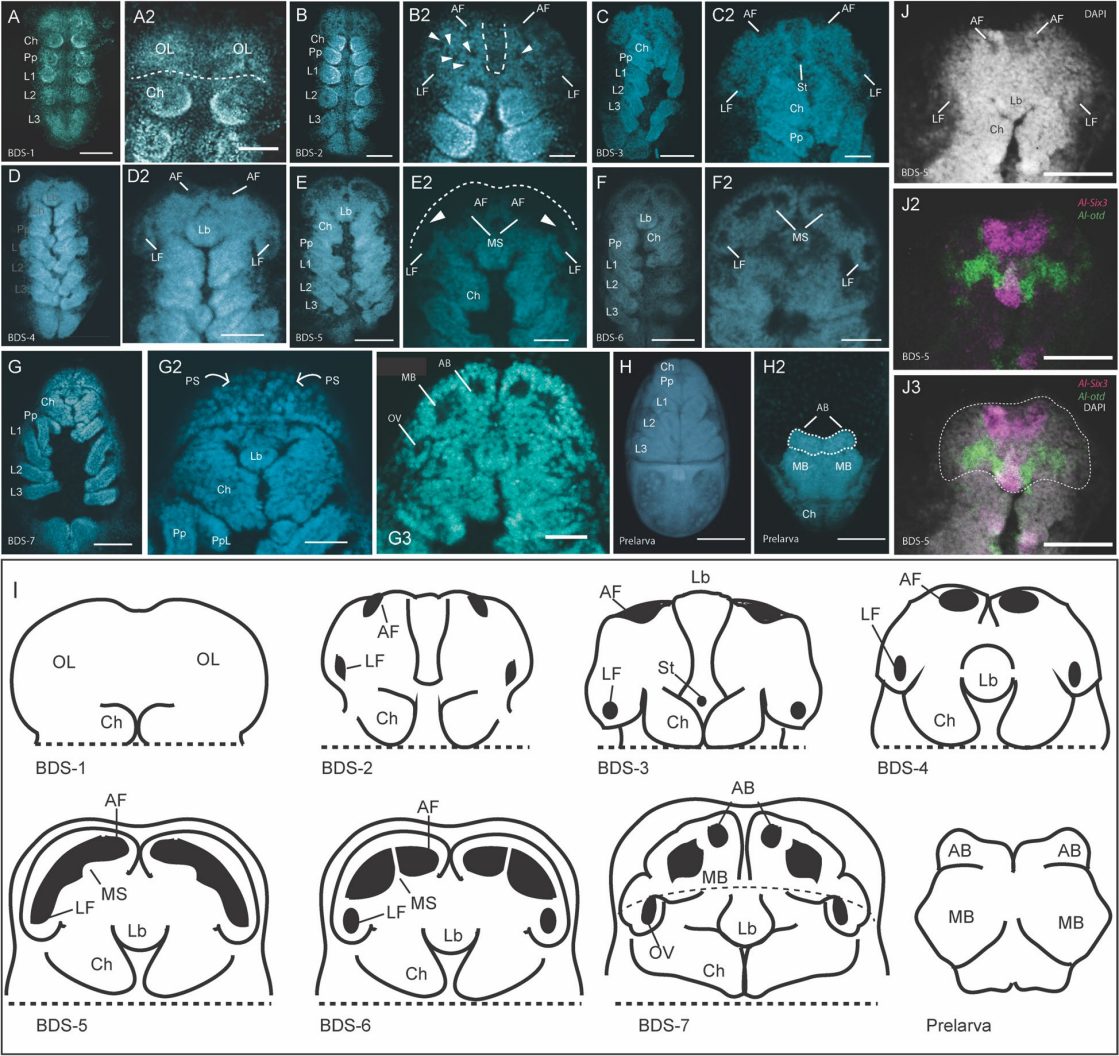
The development and compartmentalization of the *A. longisetosus* pre-cheliceral region**. A** An embryo at Brain Development Stage 1 (BDS-1). **A2** The same embryo shown in **A**, showing the paired optic lobes (OL) of the pre-cheliceral region. The dotted line demarks the boundary between the deuterocerebral region and the ocular region. **B** An embryo at stage BDS-2. **B2** shows the same embryo in **B**. The arrowheads point to “pits” in the presumptive neural tissue that are likely neural-precursor cells. Paired lateral furrows (LF) and anterior furrows (AF) are present at this stage, as well as a “groove” medially separating the optic lobes (dotted line). **C** An embryo at stage BDS-3. **C3** A closer image of the same embryo shown in **C**, showing the presence of the anterior and lateral furrows, as well as the newly formed stomodaeal opening (St.). **D** An embryo at stage BDS-4. **D2** A close-up of the head region of the same embryo shown in **D**. The labral halves have fused at this stage, and the labrum (Lb) has migrated posteriorly. **E** An embryo at stage BDS-5, and a close-up of this embryo, **E2**, showing the continuous opening formed by the fusion of the anterior and lateral furrows. The arrowheads mark a continuum between the lateral and anterior furrows, and the dotted line represents the anterior boundary of the embryo that is out of focus. Also at this stage, the medial subdivisions (MS) have begun to send out extensions that will eventually subdivide these continuous tubes. **F** An embryo at stage BDS-6, and **F2** a close-up of the head region of the same embryo. At this stage, the projections of the medial subdivisions have expanded to almost contact the lateral edges of the continuous tube of the lateral and anterior furrows. **G** An embryo at stage BDS-7. **G2** shows a ventral confocal 3D projection of this embryo. At this stage, the two halves of the prosomal shield (PS) have migrated ventrally and posteriorly to cover the developing brain region. The arrows show the movement of the prosomal shield halves. **G3** A confocal image of the same embryo in **G-G2** showing a more dorsal Z-slice. Embryos at this stage have subdivided the continuous tubes of the lateral and anterior furrows into the paired arcuate bodies (AB), mushroom bodies (MB), and optic vesicles (OV). **H** A ventral image of the prelarval stage of *A. longisetosus*. In **H2**, the embryo has been rotated so that its dorsum is shown. The brain region has migrated dorsally at this stage and appears “upside-down” in comparison to the prior stages. The dotted line demarks the fused arcuate body. **I** Schema outlining the deduced morphogenetic events shown in **A-H2.** See text for details. All embryos shown in **A-H2** are oriented with the anterior of the embryo directed towards the top of the page, however, please note that in prelarvae, the brain region is inverted as it folds over the head region. **I** Schematics of the aforementioned stages. Note that in the prelarval stage, the brain is “upside-down” in relation to the other stages. **J-J3** Images of an embryo at approximately late BDS-5, highlighting the ocular region. **J** A DAPI image of this embryo. **J2** A double-HCR showing *Al-Six3* expression (magenta) and *Al-otd* expression (green) in the ocular region. **J3** merged #D projection of this embryo with the DAPI signal. The dotted line demarks the ocular region. Abbreviations are Ch, chelicerae; L1-L3, walking legs 1–3, respectively; Pp, pedipalps. Scale bars in (**A–H**) and (**H2**) represent 50 µm. The scale bars in the remaining images represent 20 µm. All embryos shown in the confocal images were stained with DAPI

The arthropod brain and its derivatives develop from the anterior-most region of the embryo. In modern literature, this region is either called the “ocular segment”, (*e.g*., [41, 69, 70]), the “ocular region”, (*e.g*., [37]) the “pre-antennal region” if discussing mandibulates (*e.g.* [28]*,*), or the “pre-cheliceral” region when describing chelicerates (*e.g*., [71–73]). We acknowledge that the segmental nature of the arthropod brain is still a matter of debate (see [37–40]), however we adopt the term “ocular region” sensu [37] for the remainder of the paper, defined as the region anterior to the cheliceral segment in which the genes *Six3* and *orthodenticle* (*otd*) are expressed [37] (see Fig. 1J-J3). Note that the specifics of these expression patterns will be discussed in later sections of this work.

As is typical of arthropod brain development, the first stage of brain morphogenesis began with the ocular region of *A. longisetosus* bifurcating into two lateral lobes, often called the “optic lobes” during BDS-1 (Fig. 1A-A2). At BDS-2 (Fig. 1B-B2), we observed the appearance of “pits” in the ocular region (Fig. 1B2, arrowheads). We took these “pits” to be invaginating neuroblasts, based on their similarity to structures found in spider head development [9, 72–75]. This stage was also characterized by medial boundaries forming around each of the optic lobes, resulting in an antero-posteriorly oriented “groove” between them (Fig. 1B2, dotted line demarks the boundaries of this structure). Also, during BDS-2, we observed a pair of anterior-medial furrows, as well as two lateral furrows on each optic lobe. Based on comparative data from the spiders *C. salei* [9, 71, 72] and *P. tepidariorum* [10, 73], and the opilionid *P. opilio* [36, 69], we took the two lateral furrows and the two anterior furrows to correspond to the same structures seen in these arachnids (Fig. 1B-B2).

BDS-3 was characterized by the deepening of the anterior and lateral furrows into the embryo. The antero-posterior groove that subdivides each ocular lobe was maintained at this stage, and a clear stomodeal opening was present at its posterior terminus (Fig. 1C-C2). BDS-4 followed the fusion of the labral halves, and their subsequent posterior migration (see [76] for details; Fig. 1D-D2). At BDS-5, the anterior furrows expanded posterior-laterally and grew larger. Also at this stage, the anterior furrows produced a continuous opening with their adjacent lateral furrows (Fig. 1E2, arrowheads mark a continuum between the lateral and anterior furrows). This is noteworthy, as a similar morphogenetic movement has not been observed in either opilionids or spiders, which maintain distinct lateral and anterior furrows during brain morphogenesis [9, 10, 36, 69, 72, 73]. Also, at BDS-5, we observed a field of cells that were growing towards the middle of each anterior furrow (Fig. 1E2). In the spiders *P. tepidariorum* and *C. salei*, two fields of neural precursor cells, called the medial subdivisions, also appear on each of the optic lobes and partially cover the anterior furrow [9, 71–73]. Due to the similarity of these structures to those in these spider species, we take these structures to be homologous to the medial subdivisions. This is notable, as it has been proposed that the medial subdivisions give rise to the optic neuropils of the median eyes of *C. salei* (see Discussion in [9]).

At stage BSD-6, the lateral furrows had closed and had distinct boundaries (Fig. 1F-F2). The medial subdivisions extended anteriorly to contact the opposite sides of each anterior furrow (Fig. 1F2). In the spiders *P. tepidariorum* and *C. salei*, a second group of neural precursor cells, called the lateral subdivisions, migrate and partially cover the lateral furrows [9, 72, 73]. We did not observe any comparable morphogenetic movements or structures in *A. longisetosus*. This is also of interest, as these lateral furrows are presumed to form the lateral eye optic neuropils in these spiders [9].

In arachnids, the non-neurogenic ectoderm at the anterior rim of the head lobes migrates to cover the developing brain. This “hood-like” structure is often called the prosomal shield [9, 10, 36, 69, 71–73]. We observed the same structure form in *A. longisetosus*, and its downward migration was mostly complete by BDS-7 (Fig. 1G-G2). Also, at BDS-7, we observed that the medial subdivisions divided the anterior furrow into two distinct compartments. Based on observations of comparable stages of spider brain development, we take the anterior-most compartments to be the arcuate bodies (= central complexes) and the immediately posterior compartments to be the mushroom bodies (see [71], their fig. 4 compared to our Fig. 1G3). By using comparable stages in spiders, we also deduced that the lateral furrow forms the homolog to the spider optic vesicle (see [71], their fig. 4 compared to our Fig. 1G3). We use these terms to describe these structures at this stage and in subsequent stages. In the following prelarval stage, the anterior of the brain “flipped” resulting in the anterior of the brain pointing posteriorly. This movement appears to be conserved in arachnids, as this is also seen in spiders and opilionids, as well as in an extinct chelicerate [12, 36, 71, 77]. In Fig. 1H2, we demarcate the structures by dotted lines that we understand to be the arcuate bodies to highlight this morphological movement.

Taken together, the development of the *A. longisetosus* brain is like that of spiders and opilionids, albeit with lineage-specific differences. All groups develop both anterior and lateral furrows. However, these combine to form a continuous “tube” during mid-embryogenesis in *A. longisetosus*. These tubes are then subdivided to form specific compartments in the brain, *i.e.*, the optic vesicles, mushroom bodies, and the arcuate bodies. Surprisingly, structures associated with eye development in spiders are also present in *A. longisetosus*, despite their lack of eyes. These structures include the optic vesicles, as well as the formation of medial subdivisions that have been suggested to be the precursors of the optic neuropils of the median eyes [9]. See Table S3 for a comparison of the stages of the major developmental events in *A. longisetosus* with model, and emerging model, arachnids.

### The expression of genes associated with downstream eye development does not support the presence of vestigial eyes in *A. longisetosus*

Because of the similarities of *A. longisetosus* brain development to that of spiders, we asked if gene expression patterns could be used to detect embryonic eye primordia that may subsequently degenerate, in a similar manner that was used to discover the vestigial lateral eyes of *P. opilio* [36]. In the opilionid *P. opilio* and the spider *P. tepidariorum*, the opsin gene *peropsin* is expressed in the terminally differentiated visual organs, as well as the embryonic rudiments of the eyes of the opilionid after the completion of the migration of their prosomal shields [10, 36]. Therefore, to detect the possible presence of rudimentary eyes in *A. longisetosus*, we performed HCRs targeting the *A. longisetosus* ortholog of *peropsin* (*Al-peropsin*). This experiment showed no expression of *Al-peropsin* at any stage of embryonic development, in the brain or otherwise (Fig. S1A-A4). Alongside *Al-peropsin*, *Al-rhodopsin-7* was identified as the only other opsin retained in the *A. longisetosus* genome [54]. *rhodopsin-7* genes have been implicated in circadian rhythm photoreception in various taxa (reviewed in [78]). To test for the possibility that this gene may be expressed in developing, vestigial eyes, we performed HCRs targeting this gene’s expression. We also did not detect any *Al-rhodopsin-7* expression at any developmental stage in the ocular region (Fig. S1B2, C2).

Beta-arrestins also are utilized in photoreceptor specification, and their expression patterns have been recently used to identify the vestigial eyes of *P. opilio* [36]. By scouring the published *A. longisetosus* transcriptome [54], we identified three candidates for beta-arrestin orthologs. To verify these potential orthologs, a phylogenetic reconstruction was performed, which placed the transcript TRINITY_GG_5120_c51_g1_i7 in the same clade as Dm-Kurtz with high support (aLRT = 0.90). The transcript TRINITY_GG_4713_c203_g1_i1 was placed in a clade with Dm-Arrestin-2 (aLRT = 0.99), and the transcript TRINITY_GG_3318_c59_g1_i3 was placed in a clade with Dm-Arrestin-1 (aLRT = 0.99) (Fig. S2). An HCR targeting all three genes showed no expression at any BDS in the ocular region (Fig. S1B, B3, C, C3, D-D3, E-E3).

The expression of the *myosin-III* gene (known as *ninaC* in *D. melanogaster*) is expressed in the larval and adult eyes of the horseshoe crab [79], and the paralog *Po-myoIII-2* was used to detect the vestigial eyes of *P. opilio* [36]. By using several chelicerate and arthropod NinaC/Myosin-III proteins as queries, and by subsequently performing phylogenetic analyses of our possible hits, we were unable to detect any potential *myosin-III* orthologs in the *A. longisetosus* genome or transcriptome. This is noteworthy, as NinaC proteins in *D. melanogaster* are expressed in the photoreceptor cells, and their mutational abrogation results in photoreceptor defects [80]. Therefore, the absence of a *myosin-III/ninaC* in the eyeless mite is interesting for future studies into how natural selection targets photoreceptor genes in species undergoing eye loss or eye reduction. In summation, the absence of the expression of opsins and *beta-arrestins* together support the hypothesis that *A. longisetosus* lacks embryonic or vestigial eyes.

### The expression of retinal determination genes in *A. longisetosus*

The above results suggest that the downstream molecules of eye development are not activated in the mite ocular region. However, we hypothesized that vestigial eye gene expression upstream of these genes could reveal vestigial developing eye primordia. The development of arthropod eyes usually requires a conserved set of developmental genes, collectively called the RDGN [81, 82], and these genes have been shown to be expressed in the developing eyes of other arachnid taxa [9, 10, 33, 35, 36]. To detect potential vestigial eye anlagen in *A. longisetosus*, we performed HCRs targeting its orthologs of the RDGN genes *eyes absent* (*Al-eya*), *sine oculis* (*Al-so*), *dachshund* (*Al-dac*), *Six3* (*Al-Six3*), *atonal* (*Al-ato*). Below, we describe the resulting expression patterns of these RDGN orthologs.

#### *Al-eyes absent* (*Al-eya*) expression

In *Drosophila*, *eya* encodes a protein tyrosine phosphatase that acts as a transcriptional co-activator with the products of *sine oculis* and *dachshund* within the RDGN [83]. The use of *eya* in patterning the eyes of arthropods appears to be ancestral, as exemplified by its expression in chelicerate eyes, *i.e.*, spiders and opilionids [9, 10, 34, 36].

**Fig. 2:**
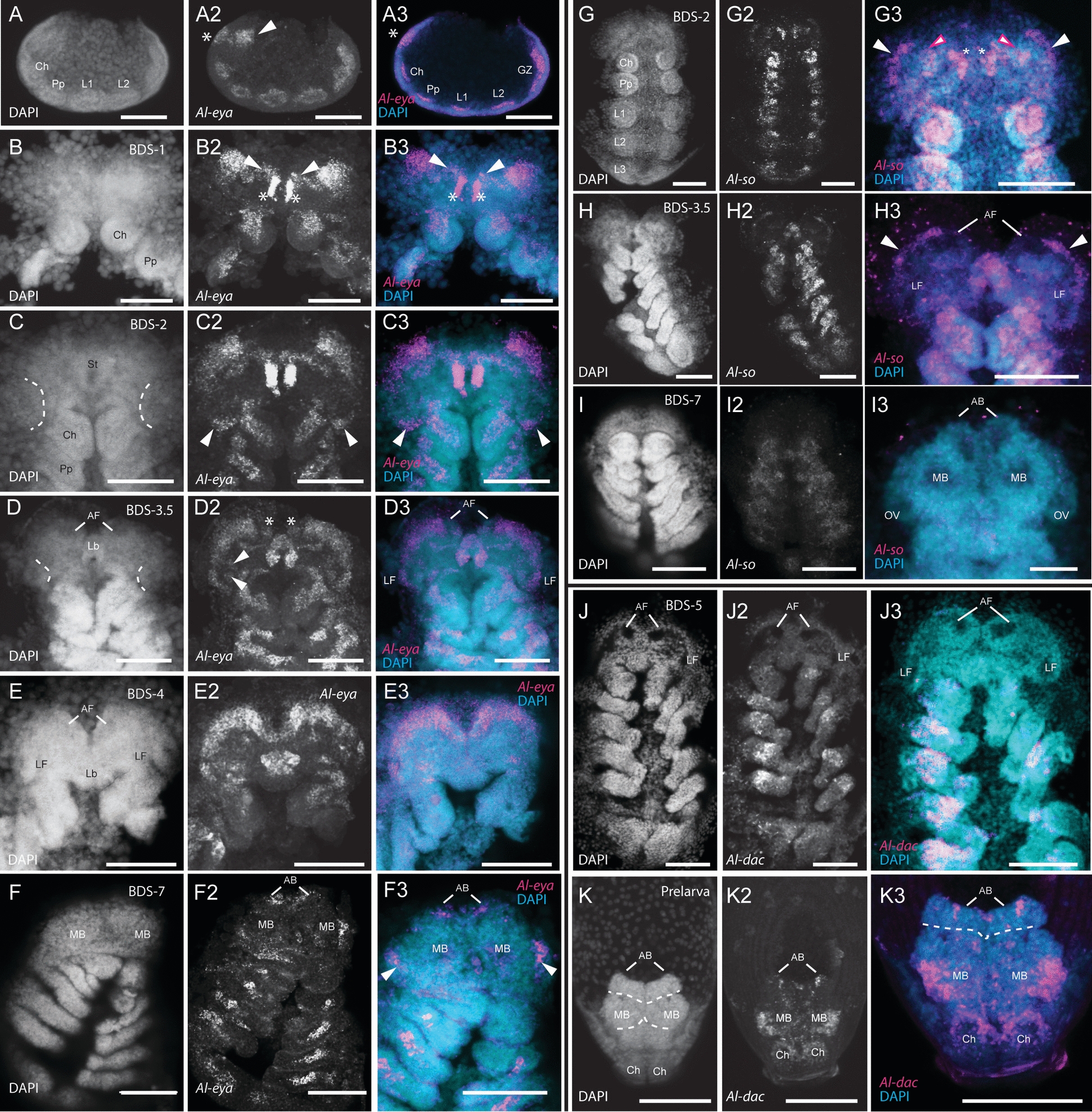
*Al-eyes absent (Al-eya), Al-sine oculis (Al-so),* and *Al-dachshund (Al-dac)* expression. **A-A3** Confocal images of *Al-ato* expression in an early segmental-stage embryo. **A** and **A2** are maximum projections, whereas **A3** is a Z-slice. The asterisks in **A2** and **A3** demark expression in the medial groove, whereas the arrowhead in **A2** points to expression in the lateral pre-cheliceral region. **B-B3** Confocal images of *Al-eya* expression in an embryo at BDS-1. The asterisks demark expression along the lines of the median groove, whereas the arrowheads demark thin lines of expression that appear at this stage. **C-C2** *Al-eya* expression in an embryo at BDS-2. The dotted lines in the DAPI image of **C** delimit the median boundaries of the lateral furrows where *Al-eya* expression appears at their posterior regions (arrowheads). **D-D3** *Al-eya* expression in an embryo at BDS-3.5. The DAPI image in **D** delimit the median boundaries of the lateral furrows. The asterisks demark *Al-eya* expression in the anterior furrows, and the arrowheads demark *Al-eya* expression in the medial lips of the lateral furrows. **E-E3** *Al-eya* expression in an embryo at BDS-4. **F-F3** *Al-eya* expression in an embryo at BDS-7. The arrowheads in **F3** demark Al-eya expression underneath the prosomal shield in the mushroom bodies. **G-G3** *Al-so* expression in an embryo at BDS-2. Magenta-outlined arrowheads in **G3** demark expression in the medial grooves, and the white arrowheads demark *expression* in two lateral domains of the pre-cheliceral region. **H-H3** *Al-so* expression in an embryo at BDS-3.5. The arrowheads demark expression in the lateral regions of the head lobes. **I-I2** *Al-so* expression in an embryo at BDS-7. At this stage, *Al-so* expression is not present in the developing head and brain. **J-J3** *Al-dac* expression at BDS-5, showing no expression in the head/brain region. **K-K3** *Al-dac* expression in the brain of a prelarva. All abbreviations are the same as in other figures. All scale bars represent 50 µm

We first observed the expression of *Al-eyes absent* (*Al-eya*) at the early stages of prosomal segmentation, when the cheliceral, pedipalpal, and the first two walking leg segments had formed (Fig. 2A-A3). At this stage, *Al-eya* expression was in each of the developing prosomal segments, as well as in a lateral domain in the ocular region (arrowhead in Fig. 2A2). This lateral expression domain is similar to the triangular expression domains of *Al-ey* at this stage (see below) and may thus be involved in the formation of the same structure (*i.e.*, the lateral furrows; discussed below). We also observed *Al-eya* in an anterior ectodermal domain (asterisk in Fig. 2A2), which likely demarks the “groove” that separates the head lobes at BDS-2 (see Fig. 1B-B2). Interestingly, we also observed *Al-eya* expression in the presumptive mesoderm of the developing prosomal segments, as well as in the growth zone of the opisthosoma (Fig. 2A3). This is markedly different from the ectodermal expression of *Al-eya* noted above. These expression patterns largely continued into BDS-1 (Fig. 2B-B3), however *Al-eya* was also expressed in two lateral expression domains that connected the bilateral triangular domains in the ocular region (arrowheads in Fig. 2B2-B3) to the medial groove-lining domains (asterisks in Fig. 2B2-B3). We take these medial groove-lining domains to be the same ectodermal domain observed in Fig. 2A2 (asterisk).

At approximately BDS-2 (Fig. 2C-C3), the bilateral domains of *Al-eya* expression moved anteriorly. The previously described *Al-eya* expression domains were retained at this stage; however, two new bilateral *Al-eya* expression domains appeared. These domains were restricted to the posterior margins of the lateral furrows (arrowheads in Fig. 2C2-C3). *Al-eya* expression was broadly retained in these domains at late BDS-3/early BDS-4 (*i.e*., BDS-3.5). Intriguingly, however, the two domains of *Al-eya* expression merged into one, lateral domain on each of the head lobes (Fig. 2D-D3). In addition, two domains of *Al-eya* expression appeared on the medial “lip” of each lateral furrow (arrowheads mark one pair in Fig. 2D2). *Al-eya* was also expressed in the anterior furrows at this stage (asterisks in Fig. 2D2). Embryos at BDS-4 displayed comparable *Al-eya* expression patterns to the previous stage (Fig. 2E-E3). Because of the current lack of reliable markers for neural vs. non-neural ocular ectoderm, we were unable to definitively interpret the expression patterns of *Al-eya* in the rims of the ocular lobes as being neural or non-neural. However, by comparing these HCRs to those of other arachnids, we take these expression patterns to be in both the non-neural and neural ectoderm (*c.f.* Fig. 2B-F3 to comparable expression in [9, 10, 34–36]). *Al-eya* expression continued into BDS-7 (Fig. 2F-F3). At focal planes underneath the prosomal shield, we identified *Al-eya* expression in the lateral margins of the mushroom bodies (arrowheads in Fig. 2F2-F3), as well as in the arcuate bodies (asterisks in Fig. 2F-F3). In these older embryos we did not detect any *Al-eya* expression in the non-neural prosomal shield that suggested either rudimentary or vestigial eyes. Taken together, we interpret these expression patterns to be consistent with the hypothesis that *Al-eya* is situated at a position to “pre-figure” eye expression in the non-neural ectoderm (*e.g.*, Fig. 2C–E) with subsequent “clearing” of *Al-eya* expression in the non-neural ectoderm of the prosomal shield at BDS-7 (Fig. 2F-F3).

#### *Al-sineoculis* (*Al-so*) expression

In *Drosophila*, the Six-family gene *so* encodes a transcription factor that interacts with several members of the RDGN and has been shown to directly form a protein complex with Eya (reviewed in [84]). We first detected *Al-so* expression in BDS-2 embryos (Fig. 2G-G3). *Al-so* was expressed in the presumptive mesoderm of the prosomal appendages, in a similar manner to *Al-eya* expression (see above). BDS-2 embryos showed *Al-so* expression in the medial grooves in a similar manner to *Al-eya* (Fig. 2G3, asterisks). *Al-so* expression was also detected in bilateral domains adjacent to the medial-groove expression domains (magenta-outlined arrowheads in Fig. 2G3) and in two lateral domains (arrowheads in Fig. 2G3). At roughly BDS-3.5, *Al-so* expression remained similar to its expression at BDS-2. We did observe, however, that the *Al-so* expression domains at the lateral edges observed at BDS-2 expanded to line the edge of each head lobe, as well as the lateral lip of each lateral furrow. After comparing these expression patterns lining the head lobes to those of other published arachnids (*e.g.* [9, 10, 33–36]*,*), we interpret these *Al-so*-positive cells to be the non-neural ectoderm (Fig. 2H-H3). As development progressed into BDS-7, the expression of *Al-so* continued in the mesoderm of the appendages (Fig. 2I-I3). However, in the ocular region expression of *Al-so* disappeared (Fig. 2I-I3). In summation, like *Al-eya* expression, *Al-so* expression in the lateral rims of the head lobes (Fig. 2G-H3) is consistent with a potential role of “pre-figuring” eye primordia with subsequent clearing of these primordia at later stages (Fig. 2I-I3).

### *Al-dachshund* (*Al-dac*) expression

In *Drosophila*, *Dm-dachshund* (*Dm-dac*) interacts with other components of the RDGN, and *Dm-dac* mutants lack eyes [85, 86]. We performed HCRs targeting the single-copy ortholog of *dac* found in the *A. longisetosus* genome (*Al-dac*). As previously reported, *Al-dac* is expressed in the medial domains of the extending embryonic limbs [59]. However, we did not observe *Al-dac* expression in the ocular region at any embryonic stage (an example is shown in a BDS-5 embryo in Fig. 2J-J3, consistent with [59]. We did, however, observe post-embryonic expression of *Al-dac* in the brains of prelarvae (Fig. 2K-K3). In these prelarvae, *Al-dac* was detected in regions we take to be the mushroom bodies, as well as in three small domains in the arcuate bodies (Fig. 2K-K3).

#### *Al-Six3* expression

The six-family transcription factor Six3/Optix has a highly conserved role in demarking the anterior-most region of animal embryos [87]. In arthropods, this region has been proposed to be the prosocerebrum of the brain [40]. In addition to its role of anterior head regionalization, *Six3* is also involved in the formation of animal eyes (*e.g.*, [88]). In *Drosophila*, *Six3* is required for the progression of the morphogenetic furrow of the developing retinas in the eye/antennal imaginal discs [89].

**Fig. 3:**
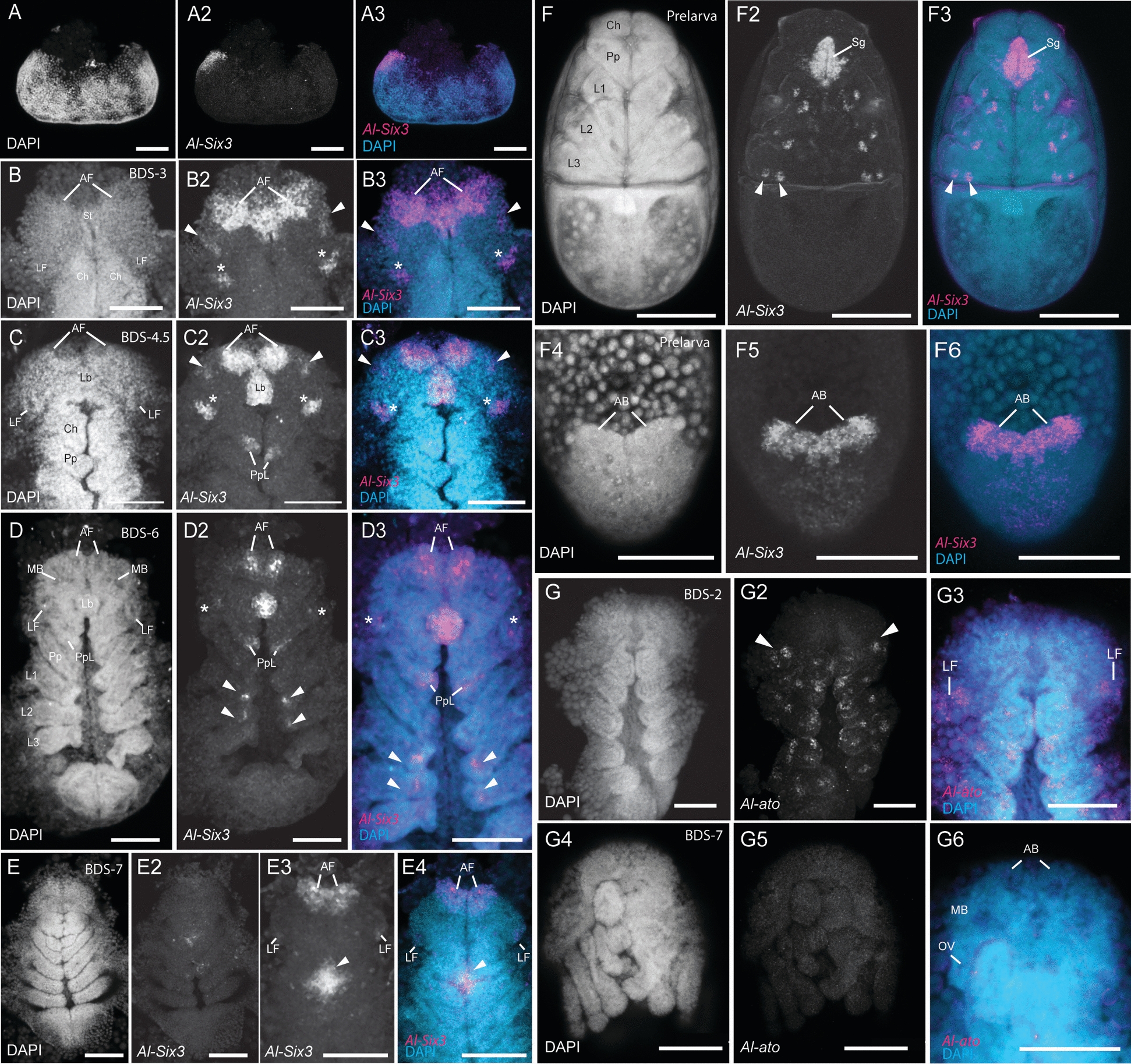
*Al-Six3* and *Al-atonal (Al-ato)* expression. **A-A3** *Al-Six3* expression in an early germ band embryo. **B-B3** *Al-Six3* expression at BDS-3. The arrowheads in **B2-B3** demark expression in the incipient mushroom bodies. **C-C3** *Al-Six3* expression at approximately BDS-4.5. The arrowheads in **C2-C3** demark expression in the incipient mushroom bodies, and the asterisks demark expression in the lateral furrows. **D-D3** *Al-Six3* expression at BDS-6. Asterisks demark expression in the lateral furrows, whereas the arrowheads demark expression in the tips of the developing legs. **E-E4** *Al-Six3* expression at BDS-7. The arrowheads demark potential expression in the synganglion. **F-F3** *Al-Six3* expression in a prelarva. Note the expression in the synganglion (Sg). **F4-F6** *Al-Six3* expression in the same prelarva, rotated to the dorsum to visualize expression in the brain. **G-G3** *Al-ato* expression in an embryo at BDS-2. The arrowheads in **G2** demark expression in the lateral furrows. **G4-G6** An HCR of *Al-ato* showing lack of expression at BDS-7. All abbreviations are the same as in other figures. All scale bars represent 50 µm

In *A. longisetosus*, we observed *Al-Six3* expression in an early germ band stage (Fig. 3A-A3). At this stage, *Al-Six3* was detected in the anterior-most region of the embryo, consistent with observations in other animal taxa [87]. At approximately BDS-3, *Al-Six3* was observed in a large anterior domain that spanned and connected the anterior furrows (Fig. 3B-B3). We also detected *Al-Six3* expression in two domains within the neuroectoderm that we take to form the mushroom bodies (arrowheads in Fig. 3B2-B3) as well as in two domains that were in the region of the lateral furrows (arrowheads in Fig. 3B2-B3). As embryogenesis progressed to approximately BDS-4.5, *Al-Six3* expression was retained in the neuroectodermal anterior furrows (Fig. 3C-C3). The mushroom-body associated expression was also retained (Fig. 3C2-C3, arrowheads). The expression of *Al-Six3* in the lateral furrows of this stage was restricted to the lateral edges of each furrow (Fig. 3C2-C3, asterisks). *Al-Six3* was also present in the labrum, as well as in the extending pedipalpal lobes (Fig. 3C2; note that these are underneath the chelicerae at this stage; see [59, 76]). At approximately BDS-6, *Al-Six3* expression remained in the neuroectoderm of the ocular region (Fig. 3D-D3). However, the expression of *Al-Six3* in the lateral furrows was markedly reduced and appeared to be restricted to their center (Fig. 3D2-D3, asterisks). Additional differences in *Al-Six3* expression from the previous stages include its expression in the distal tips of the first and second pairs of walking legs (Fig. 3D2, arrowheads), as well as stronger expression in the extended pedipalpal lobes. Also, *Al-Six3* expression in the mushroom bodies disappeared by this stage. In these stages, we did not detect *Al-Six3* transcripts in the non-neural head ectoderm. This was made further evident by *Al-Six3* expression at BDS-7, at which point the prosomal shield has migrated over the neural ectoderm (Fig. 3E-E3). At BDS-7, *Al-Six3* expression was completely absent in the non-neural prosomal shield (Fig. 3E2). However, in Z-stacks deeper into the embryos, *Al-Six3* expression was still present in the anterior furrows/ arcuate bodies, as well as in the lateral furrows/optic vesicles (Fig. 3E3-E4). We also observed “patches” of *Al-Six3* expression in the midline of each embryo at this stage (Fig. 3E3-E4; arrowheads). In prelarvae, *Al-Six3* expression persisted (Fig. 3F-F6). *Al-Six3* expression in prelarvae was observed in two “spots” of expression in each of the walking legs (Fig. 3F2-F3; arrowheads demarcate two such spots in a third walking leg). Additionally, *Al-Six3* was ubiquitously expressed in a structure whose position and shape suggest that it is the synganglion (*i.e.*, the fused and compacted ventral nerve cord found in mites and ticks [90]). *Al-Six3* was also observed in the arcuate bodies at this stage, as well as in punctate domains in the anterior brain (Fig. 3F4-F6). From these observations, we conclude that *Al-Six3* likely does not participate in the formation of vestigial eye primordia.

#### *Al-atonal* (*Al-ato*) expression

In *Drosophila*, the product of *atonal* (*Dm-ato*) is activated by the products of *Dm-so* and *Dm-eya* to initiate photoreceptor development (reviewed in [84]). We detected the earliest expression of the singleton *atonal* ortholog in *A. longisetosus* (*Al-ato*) in BDS-2 embryos (Fig. 3G-G3). In these embryos, *Al-ato* was expressed in “clusters” of cells in the developing prosomal appendages. In the ocular region, *Al-ato* expression was observed in two clusters of cells in each optic lobe, in regions straddling each presumptive lateral furrow within the neural ectoderm (Fig. 3G2, arrowheads). We did not observe *Al-ato* expression in the ocular region in subsequent stages (Fig. S1F-F2 shows no expression in a BDS-5 embryo; Fig. 3G4-G6 shows the absence of expression in the prosomal shield of an embryo at BDS-7).

#### Expression of the *Pax6* gene *Al-eyeless* (*Al-ey*)

**Fig. 4:**
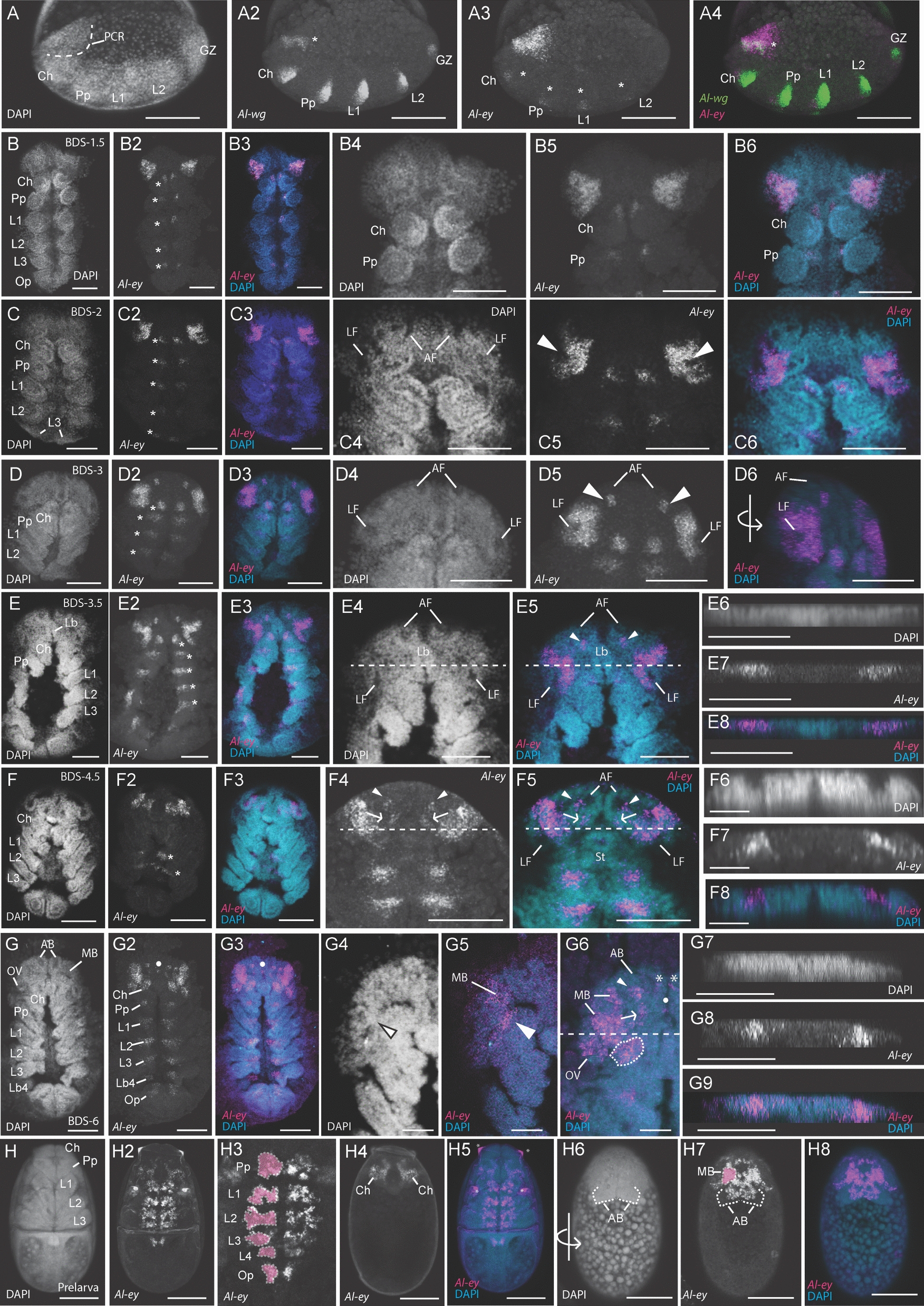
*Al-eyeless* (*Al-ey*) expression. **A-A4** Confocal images of a double hybridization chain reaction (HCR) in an early, prosomal-segmentation stage embryo targeting both *Al-wingless* (*Al-wg*) and *Al-ey*. All images of this embryo are oriented to show its lateral side and its anterior is directed towards the left of the page. **A** DAPI nuclear counterstain image of this embryo, showing the location of the pre-cheliceral region (PCR), outlined in a dotted line. **A2** *Al-wg* is expressed in each of the developing prosomal segments at this stage, as well as in the segmental growth zone (GZ) and in a stripe of expression in the pre-cheliceral region (asterisk). **A3** *Al-ey* is expressed in this same embryo in a triangular-shaped domain of expression, and also in paired, clusters of cells in each of the prosomal segments (asterisks). **A4** A merged confocal image of this embryo showing *Al-wg* expression (green) concurrently with *Al-ey* expression (magenta). Note the co-expression of both genes in the pre-cheliceral region (asterisk). **B-B6** An embryo at a stage approximately between BDS-1 and BDS-2 (BDS-1.5). **B** DAPI channel. **B2** *Al-ey* expression in the same embryo, showing the retention of *Al-ey* expression in triangular domains in the pre-cheliceral region, and in paired clusters of expression in each of the prosomal segments (asterisks). **B4-B6** Confocal images of the same embryo, zoomed in to highlight the morphology of the pre-cheliceral region **B4** DAPI channel. **B5** *Al-ey* expression in this region. **B6** Both channels merged. **C-C6** Confocal images of a single embryo at BDS-2. **C** Nuclear counterstain with DAPI. **C2** Expression of *Al-ey* in this embryo, showing the retention of *Al-ey* expression in paired domains of the pre-cheliceral region, and also the paired expression in clusters of each prosomal segment (asterisks). **C3** Merged image of both the DAPI counterstain (blue) and *Al-ey* expression (magenta). **C4-C6** Confocal images of this embryo zoomed-in to show the appearance of the anterior and lateral furrows (**C4**, AF and LF, respectively). *Al-ey* expression “clears” from the lateral furrows at this stage (arrowheads, **C5**)_._ **C6** Merged confocal image of DAPI (blue) and *Al-ey* expression (magenta). **D-D6** Confocal images of an embryo at late BDS-3. **D** Nuclear counterstain with DAPI. **D2** Confocal image of *Al-ey* expression in this embryo, showing the retention of *Al-ey* expression in the pre-cheliceral region and in the paired clusters of the prosomal segments (asterisks). **D3** merged image of DAPI (blue) and *Al-ey* expression (magenta). **D4-D6** Zoomed-in confocal images of this embryo. At this stage, the anterior and lateral furrows are more distinct (**D4**). **D5** *Al-ey* expression is “ring-like” at this stage, as it surrounds the deepening lateral furrow. Two additional clusters of expression are also present at this stage (arrowheads), just posterior to each anterior furrow. **D6** A 3D-projection of this embryo, showing the merged DAPI (blue) and *Al-ey* (magenta) channels. The embryo has been rotated (see rotated arrow for orientation) to show the absence of *Al-ey* expression in the lateral furrows. **E-E5** Confocal images of a single embryo at BDS-3.5. **E** DAPI nuclear counterstain. **E2** *Al-ey* expression in this embryo, showing the retention of *Al-ey* in the pre-cheliceral region and in the paired, segmental clusters of the prosomal segments (asterisks). **E3** Merged image of DAPI (blue) and *Al-ey* expression (magenta). **E4** Zoomed-in image of the same embryo showing the DAPI nuclear counterstain of the pre-cheliceral region. **E5** *Al-ey* expression (magenta) merged with the DAPI counterstain (cyan) showing the “opening” of the *Al-ey* expression domains around the lateral furrows (see text for details). Arrowheads point to the *Al-ey* expression domains at the posterior of the anterior furrows. Dotted lines in **E4-E5** mark the position of the orthogonal slices shown in **E6-E8**. **E6** Confocal orthogonal slice through the antero-posterior axis of this embryo. **E7** *Al-ey* expression in the same orthogonal slice. **E8** Merged image of the DAPI (cyan) and *Al-ey* (magenta) confocal channels in the same orthogonal slice. **F-F5** Confocal images of an embryo at stage BDS-4.5. **F** DAPI nuclear counterstain of this embryo. **F2** *Al-ey* expression in this embryo. Note that the paired domains in the prosomal segments are retained at this stage, however only those of the second and third walking legs are visible (asterisks). **F3** Merged confocal image of this embryo showing the DAPI counterstain (cyan) and *Al-ey* expression (magenta). **F4** *Al-ey* expression in a Z-slice towards the dorsum of the same embryo. **F5** Merged confocal image of the same embryo, showing the DAPI counterstain (cyan) and *Al-ey* expression (magenta). In both **F4** and **F5**, arrowheads point to *Al-ey* expression in clusters associated with the anterior furrows, and the arrows point to newly appeared clusters of *Al-ey* expression posterior to these. Dotted lines in **F4-F5** mark the position of the orthogonal slices shown in **F6-F8. F6** DAPI image of this orthogonal slice. **F7** *Al-ey* expression in this orthogonal slice, showing the internalization of *Al-ey* positive cells migrating inward. **F8** Merged DAPI and *Al-ey* channels in this orthogonal slice. **G-G6** Confocal images of an embryo approximately at BDS-6. **G** DAPI image of this embryo. **G2** *Al-ey* expression in this embryo. **G3** Merged DAPI and *Al-ey* expression channels in this embryo. Note the appearance of paired clusters of *Al-ey* expression in the fourth walking leg segment bearing the fourth limb buds (Lb4) and also in paired clusters in the opisthosoma (Op). Furthermore, note the appearance of a new, central cluster of cells expressing *Al-ey* just above the labrum (arrowheads in **G2** and **G3**). **G4** DAPI image of this embryo, zoomed-in to show the structure of the pre-cheliceral region at this stage. The arrowhead points to the closing boundary separating the mushroom body from the arcuate body. **G5** Merged DAPI (cyan) and *Al-ey* (magenta) channels, showing expression in this closing boundary (arrowhead), and in the surface of the region above the mushroom body (MB). **G6** Dorsal Z-slice of this embryo, showing the internalized *Al-ey* expression in the pre-cheliceral region (see text for details). The horizontal, dotted line demarks the region of the orthogonal slices shown in **G7-G9**. **G7-G9** Orthogonal confocal slices through the pre-cheliceral region showing the internalization of *Al-ey* positive cells. **G7** DAPI channel. **G8** *Al-ey* expression. **G9** Merged DAPI channel (cyan) and *Al-ey* channel (magenta). **H-H8** *Al-ey* expression persists in the prelarval stage. **H** DAPI stain. **H2** *Al-ey* expression. **H3** *Al-ey* expression in the same prelarval. The paired, segmental *Al-ey* expressing clusters have become more complex. The left-most clusters are highlighted in pink and outlined. **H4** Z-slice showing the more dorsal cheliceral expression of *Al-ey*. **H5** Merged DAPI (cyan) and *Al-ey* expression (magenta) channels. Asterisks denote artefactual cuticle staining. **H6-H8** Confocal images of the same embryo, rotated to show the dorsally migrated brain/pre-cheliceral region at this stage. **H6** DAPI stain; the arcuate bodies (AB) are outlined with a dotted line. **H7** *Al-ey* expression in this region, showing its expression in the mushroom bodies (MB) and the arcuate bodies. The left mushroom body is outlined and highlighted in pink. **H8** Merged DAPI (cyan) and *Al-ey* expression (magenta) channels. Scale bars represent 50 µm in all images, except in (**F6-F8**) and (**G4-G6**), where they represent 20 µm. All other abbreviations are the same as in other figures

We identified singleton orthologs of each of the *Pax6* genes, *Al-ey* and *Al-toy* (see Supplemental Text and Fig. S1). We initially performed HCRs simultaneously targeting *Al-ey* with the segmentation gene *Al-wingless* (*Al-wg*), which has been used as a marker for early segmentation stages in a variety of arthropods (reviewed in [91]). Using this methodology, we detected the earliest expression of *Al-ey* during the prosomal segmentation stage preceding BDS-1, when the first four prosomal segments had been delineated (*i.e.*, the cheliceral, pedipalpal and first two walking leg segments; Fig. 4A-A4). *Al-ey* expression was observed in two paired, triangular-shaped domains within the ocular region (Fig. 4A3). Embryos of this stage have an additional domain of *Al-wg* expression in the ocular region (Fig. 4A2, asterisk), which has also been observed in a number of chelicerates during head/brain development [92]. Our double HCRs of *Al-ey* and *Al-wg* revealed that *Al-ey* expression in the ocular region encompasses this *Al-wg* domain (Fig. 4A4). At this early stage, we also observed paired clusters of expression in each of the developing prosomal segments (Fig. 4A3-A4; asterisks in 4A3). These expression domains of *Al-ey* were also observed at a later stage between BDS-1 and BDS-2 (*i.e*., BDS-1.5; Fig. 4B-B6) in the nascent tissue of the ventral nerve cord (Fig. 4B2, asterisks). This segmental expression of *Al-ey* was present in all subsequent stages of *A. longisetosus* embryogenesis, leading up to the prelarval stage.

At BDS-2, we observed the “clearing” of *Al-ey* expression in the newly formed lateral furrows (Fig. 4C-C6; arrowheads in C5; note the left lateral furrow in C4 is obfuscated by the surrounding tissue). This expression pattern was maintained in late BDS-3, with *Al-ey* expression present in a ring-like domain surrounding the deepening lateral furrows (Fig. 4D-D6). We also detected additional expression in two clusters of cells just posterior to the anterior furrows (Fig. 4D5, arrowheads). At BDS-3.5, the ring-like domain of expression was transformed into a “C-shaped” expression pattern, leaving *Al-ey* expression in the ventral-most cells of the periphery of the lateral furrows (Fig. 4E-E8). This appeared to be the result of the ring of expression observed in BDS-3 “breaking” at its dorsum. Additionally, *Al-ey* expression was maintained in the clusters of cells posterior to the anterior furrows (Fig. 4E5, arrowheads). In arachnids, the process of brain development involves the internalization of neural tissues (*e.g*., [71]). Therefore, we also asked whether, and when, a comparable internalization occurs in *A. longisetosus* through visualizing orthogonal views of *Al-ey* stained embryos. Using this method, we observed that at BDS-4, the bilateral *Al-ey* positive cells were still embedded within the surrounding tissue (Fig. 4E6-8). These data are consistent with the hypothesis they are beginning to internalize into the surrounding tissue, however live-imaging embryos during this process would be needed to verify this.

At approximately BDS-4.5, we identified *Al-ey* expression in clusters of cells on the ventral margins of the region of the “opened” lateral furrow, as well as the insides of this combined lateral and anterior furrow (Fig. 4F-F8). This suggests that the *Al-ey* expressing cells in the periphery of the lateral furrow at BDS-3 and 4 internalized as the lateral and anterior furrows form a continuous tube. To explore this further, we also imaged *Al-ey* expression along an orthogonal plane in a similar region to that shown in Fig. 4E6-8. This revealed the presence of *Al-ey* expressing cells surrounded by the edges of the “tube” made by the fusion of the lateral and anterior furrows (Fig. 4F6-8). *Al-ey* expression was also retained in two small domains at the posterior-lateral region of each anterior furrow (Fig. 4F4-F5; arrowheads). Additionally, another pair of small domains appeared at this stage posterior to the initial pair (Fig. 4F4-F5; arrows).

At approximately BDS-6, the *Al-ey* expression patterns became more complex. *Al-ey* expression was maintained in segmental clusters in the developing central nervous system (CNS), however additional clusters appeared in the fourth walking leg segment as well as in the opisthosoma (see [57, 60] for explanations on the divergent posterior segmentation in this species). Within the developing brain region, *Al-ey* expression was mostly internalized, however some external (*i.e.*, surface-level) expression did remain. Of note, a small cluster of *Al-ey* positive cells emerged in the center of the ocular region, just anterior to the labrum (Fig. 4G2, G3 and G6, dots). Surface-level expression was also found at the closing border of the continuous anterior and lateral furrows at the site of the lateral furrows’ anterior border (Fig. 4G4-5, arrowheads). Additionally, we observed external *Al-ey* expression in the region above the incipient mushroom bodies (Fig. 4G4-5). Further towards the dorsal Z-axis, we observed *Al-ey* expression in the posterior-lateral region of the arcuate bodies (Fig. 4G6, arrowhead). We take these to be the same *Al-ey* expressing cells that we observed in a similar location at BDS-5 (Fig. 4E5 and F5). The two clusters of *Al-ey* expression just posterior to these were also retained at this stage (Fig. 4G6, arrow; compare to Fig. 4F4 and F5, arrows). Two additional clusters of *Al-ey* expression were likewise seen in the medial portion of each arcuate body (Fig. 4G6, asterisks). *Al-ey* positive cells were also found in the newly compartmentalized optic vesicles, as well as inside of the incipient mushroom body region of the closing tubes (Fig. 4G6). An orthogonal view along the frontal plane revealed that these *Al-ey* expressing cell clusters of the mushroom bodies took on a triangular shape, with their vertices pointing ventrally (Fig. 4G7-9). This contrasted with the shape of these clusters in BDS-5 and may indicate a pattern of internal migration via changes in cell shape, (*e.g.*, apical constriction) as the continuous lateral furrow/anterior furrow tubes close.

We also detected *Al-ey* expression in the post-embryonic prelarval stage, where its segmental expression was maintained from earlier stages (Fig. 4H-H8). *Al-ey* expression in the segmental clusters of the CNS became more complex, likely reflecting the differentiation of the neural cells expressing this gene. It is also important to note that the segmental CNS expression of the cheliceral segment moved to a more dorsal position. *Al-ey* was also expressed in the dorsal-most region of the brain, occupying the same space as the arcuate body, and in larger and more anterior paired expression domains, which we take to be the mushroom bodies (Fig. 4H6-H8).

Taken together, our results indicate that *Al-ey* is expressed in the developing lateral furrows/optic vesicles, the mushroom bodies, and the components of the anterior furrow/arcuate body during embryogenesis. Furthermore, *Al-ey* expression persists in the CNS of post-embryonic stages. We did not observe *Al-ey* expression at these stages in the cell populations taken to be the precursors to the embryonic eyes, *i.e.*, the non-neural ectoderm of the head lobes.

#### Expression of the *Pax6* gene *Al-twin of eyeless* (*Al-toy*)

**Fig. 5:**
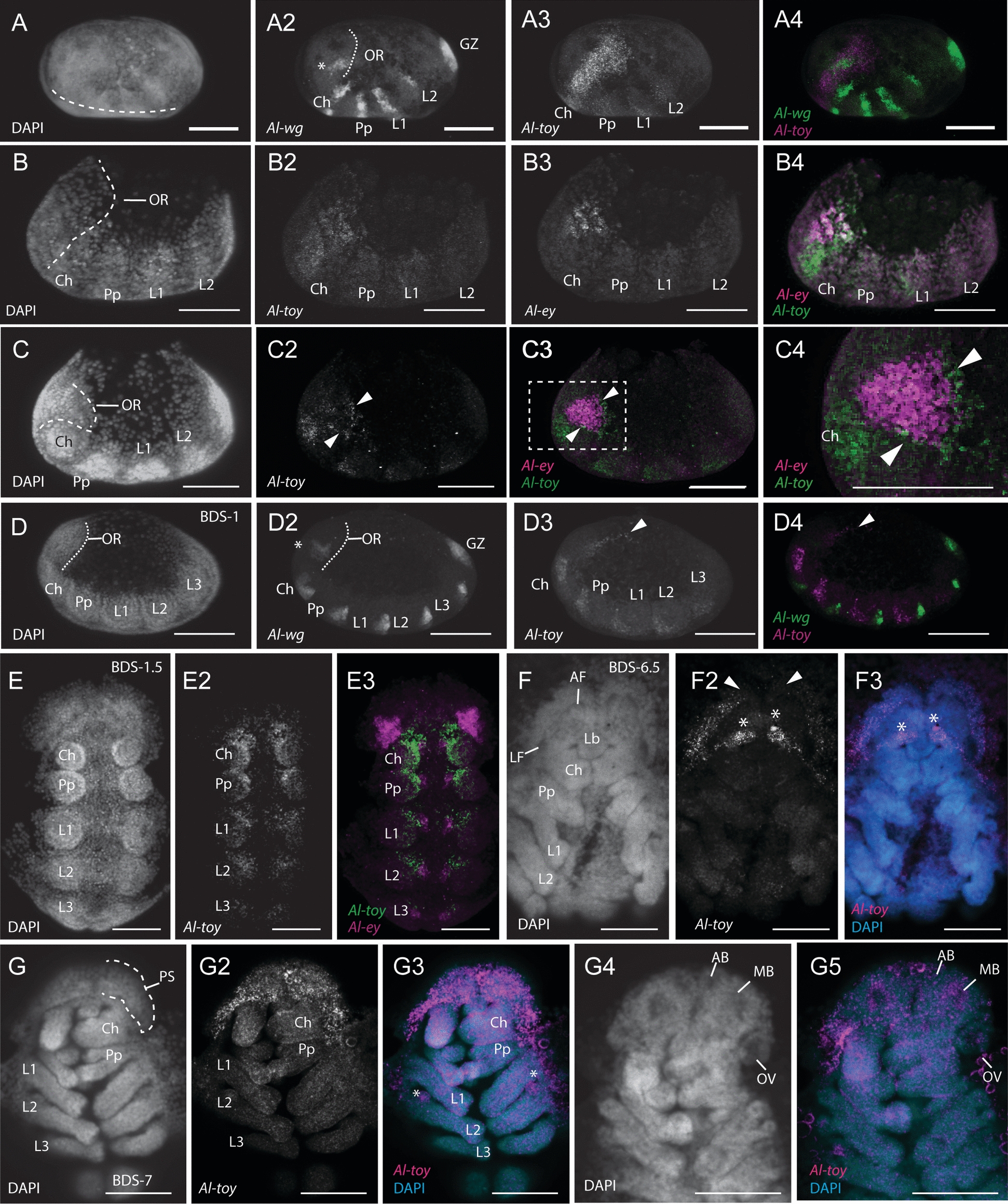
*Al-twin of eyeless* (*Al-toy*) is dynamically expressed throughout development. **A-A4** An early blastoderm/ prosomal-segmentation stage embryo. Note that the embryo is slightly “tilted” showing some expression patterns that are on the other lateral side of the embryo. **A** DAPI nuclear counterstain of this embryo. The dotted line represents the midline of the embryo. **A2** *Al-wg* expression in this embryo, showing its expression in stripes of the first four prosomal segments, in a stripe in the ocular region (asterisk), and in the growth zone (GZ). The ocular region (OR) is outlined. **A3** Confocal image of *Al-toy* expression in this embryo. *Al-toy* is expressed in a broad domain in the cheliceral segment and its extension into the pre-cheliceral region. *Al-toy* was also weakly expressed in the developing prosomal limb buds. **A4** A merged confocal image showing simultaneous *Al-wg* (green)and *Al-toy* (magenta) expression. **B-B4** A slightly older blastoderm stage embryo. **B** DAPI counterstain of this embryo. The ocular region is outlined in a dotted line. **B2** *Al-toy* expression in a broad anterior domain. **B3** *Al-ey* expression in the same embryo in a triangular domain in the ocular region. **B4** *Al-toy* (green) and *Al-ey* (magenta) expression overlap at this stage. **C-C4** *Al-toy* and *Al-ey* co-expression at an early, four limb bud-stage embryo. **C** Nuclear DAPI counterstain of this embryo. The ocular region is outlined. **C2** *Al-toy* is expressed in the anterior of each limb bud, and in boundary-like domain (arrowheads). **C3** This *Al-toy* boundary (green) “outlines” *Al-ey* (magenta) expression. The dotted-lined box represents the region zoomed into in **C4,** which shows the mutually exclusive expression domains of both *Pax6* orthologs. The arrowheads demark the *Al-toy* “boundary” type expression pattern. **D-D4** Confocal images of an embryo just prior to BDS-1. **D** DAPI counterstain of this embryo with the ocular region outlined. **D2** *Al-wg* expression. **D3** *Al-toy* expression in the anterior of the prosomal limb buds, and in a thin domain in the pre-cheliceral region (arrowhead). **D4** Merged image of *Al-wg* and *Al-toy* expression. The arrowhead demarks the thin *Al-toy* expression domain in the ocular region. **E-E3** Confocal images of an embryo at approximately BDS-1.5. **E** DAPI counterstain of this embryo. **E2** *Al-toy* expression in the developing limb buds. Note the absence of expression in the ocular region. **E3** Co-stain of both *Al-toy* (green) and *Al-ey* (magenta) expression. Note that both genes are not co-expressed at this stage. **F-F3** Confocal images of an embryo at approximately at stage BDS-6.5. **F** DAPI counterstain of this embryo. **F2** *Al-toy* is expressed in the lateral non-neural ectoderm, in paired clusters above the labrum (asterisks), and in lines of expression connecting these to the lateral ectoderm (arrows). Arrowheads mark the anterior ocular non-neural ectoderm that lacks *Al-toy* expression. **F3** Merged confocal image of the DAPI (cyan) channel and the *Al-toy* (magenta) channel. **G-G5** Confocal images of an embryo approximately at stage BDS-7. **G** DAPI counterstain of this embryo. The dotted line outlines one half of the prosomal shield (PS). **G2** *Al-toy* is expressed in migrating prosomal shield. **G3** Merged confocal images of DAPI (cyan) and *Al-toy* (magenta). The asterisks denote expression in the Claparede’s organs. **G4** DAPI counterstain of a Z-slice deeper into the embryo. **G5** Merged confocal images of DAPI (cyan) and *Al-toy* (magenta) in this Z-slice. *Al-toy* is notably expressed in the medullae of the mushroom bodies (MB). Embryos in (**A-D4**) are oriented with their anterior poles directed towards the left of the page. Embryos in the remaining images are oriented with their anterior poles directed towards the top of the page. All scale bars represent 50 µm. All other abbreviations are the same as in other figures

As with *Al-ey*, we initially co-stained embryos for *Al-toy* expression simultaneously with the segmentation gene *Al-wingless* (*Al-wg*). We detected the earliest expression patterns of *Al-toy* during a similar early blastoderm stage as shown in Fig. 4A-A4, *i.e.*, when the first four prosomal segments had been delineated by *Al-wg* (Fig. 5A-A4). Also at this stage, the ocular stripe of *Al-wg* was present. *Al-toy* was expressed in a broad domain that extended diagonally from the anterior to the posterior of the embryo from the cheliceral segment. Its posterior domain broadened into the ocular region where it covered the ocular *Al-wg* stripe (Fig. 5A3-A4). *Al-toy* was also expressed weakly in the developing limb buds (Fig. 5A3).

In spiders and daddy-longlegs, the *Pax6* orthologs are often expressed at the same embryonic stage and appear to have specific early-stage expression domains [9, 10, 33–36, 93]. We therefore performed HCRs simultaneously targeting both *Al-ey* and *Al-toy* to resolve when both orthologs are potentially co-expressed during the development of the ocular region. We observed the co-expression of *Al-ey* and *Al-toy* in embryos at the same stage shown in Fig. 5A (Fig. 5B). At this early germ-band stage, we observed *Al-toy* expression in the ocular region (Fig. 5B2), indicative of its slight anterior migration from the cheliceral segment (see Fig. 5A-A4). Low-level *Al-toy* expression was also present in the developing prosomal appendages. Interestingly, the triangular *Al-ey* expression domain (first shown in a slightly later stage in Fig. 4A-A4) completely overlapped with this *Al-toy* domain (Fig. 5B3-4B4). This is comparable to the co-expression of both *toy* and *ey* in the spider *P. tepidariorum* during its stage 8.1 and 8.2 [93], and thus likely represents a conserved feature for these genes in arachnids.

Subsequently, when the prosomal limb-buds have begun to grow more distinct, the overlap between the *Pax6* genes decreased (Fig. 5C-C4). The expression of *Al-ey* was retained in its anterior, triangular domain. However, *Al-toy* was expressed at the margins of the ocular region, *i.e.*, at the anterior cheliceral segment boundary, and at the lateral boundaries marking the lateral edges of the presumptive ocular lobes (Fig. 5C2 and C3; arrowheads). Co-staining with *Al-ey* revealed that this ocular expression of *Al-toy* encompassed the triangular domains of *Al-ey* expression, with *Al-toy* expression being “cleared” from the region of *Al-ey* expression (Fig. 5C3 and C4). *Al-toy* expression was also retained in the first four prosomal limb buds; however, it was restricted to the anterior of each developing limb (Fig. 5C-C3).

At the onset of BDS-1, *Al-toy* expression was maintained in the anterior portion of the first four pairs of limb buds and also appeared in the third walking leg buds (Fig. 5D-D4). We also observed *Al-toy* expression in a thin domain trailing from anterior to posterior from the ocular region. Based on the position of this trailing domain, as well as its position near the ocular *Al-wg* stripe (Fig. 5D2–D4), we take this to be the remnant of *Al-toy* expression seen in the ocular region of the preceding stages (Fig. 5D3 and 5D4; arrowheads). At approximately BDS-1.5, all ocular expression of *Al-toy* was absent, however *Al-toy* expression in the developing limbs persisted (Fig. 5E-E4). We noticed that the expression of *Al-toy* in the developing limbs was reminiscent of the segmental expression of *Al-ey* in the embryonic midline (Fig. 5C-C3). We therefore asked to what extent *Al-toy* was co-expressed with *Al-ey* at this stage. Our double-HCR targeting both transcripts revealed that these paralogs were not segmentally co-expressed, with *Al-toy* being restricted to the limb buds, and *Al-ey* being restricted to the CNS (Fig. 5E3).

*Al-toy* expression was not detected in subsequent stages (not shown) until approximately BDS-6.5 (Fig. 5F-F3). This is notable, as *toy* orthologs are expressed at comparable stages in spiders [9, 10, 33, 34] and opilionids [36]. At BDS-6, *Al-toy* expression was absent from the developing appendages*. Al-toy* transcripts were detected, however, in the lateral boundaries surrounding the optic lobes, as well as in the dorso-lateral margins of the lateral furrows. In spiders, these lateral margins have been described as the non-neural ectoderm that eventually migrates to form the prosomal shield (see [9, 72, 73]). *Al-toy* was also expressed in a pair of domains just above the labrum (Fig. 5F2, asterisks), and in a line of cells connecting these domains to the lateral optic lobe domains (Fig. 5F2, arrows). *Al-toy* expression was also absent from the anterior-most region of the optic lobes (Fig. 5F2, arrowheads). Comparable expression patterns are not seen for *toy* in either spiders [9, 10, 33, 34] or opilionids [36].

At approximately BDS-7, *Al-toy* expression was ubiquitous in the prosomal shield (Fig. 5G-G3), confirming our hypothesis that *Al-toy* expression in the previous stage (i.e., Fig. 5F-F3) was in the non-neural ectoderm of the head lobes. This is striking, as neither *Pax6* ortholog is expressed in the developing prosomal shield in spiders. However, in the opilionid *P. opilio*, its *toy* ortholog was expressed in the leading margin of the migrating prosomal shield ([36]; their Fig. S2). Thus, our observations may represent a lineage-specific use for *toy* in mites. We additionally observed *Al-toy* expression in the Claparede’s organs. These organs are modified coxal extensions of the second walking legs that act to aid in water uptake in *A. longisetosus* larvae (see [76] for notes on their development). Also, deeper into the embryo, we observed punctate expression of *Al*-*toy* in the developing brain, as well as its expression in the interior medullae of the mushroom bodies (Fig. 5G4–G5). We did not observe *Al-toy* in any subsequent stages, including the prelarval stage, following BDS-7.

### Early co-expression of the *Al-Pax6* paralogs and the head-patterning gene *orthodenticle*

In a wide array of arthropod exemplars, *orthodenticle* orthologs are co-expressed with *Pax6* genes in the protocerebral region of the brain (see [40] and arguments therein for a summary). We reasoned that, if we observed early *Al-ey* and/or early *Al-toy* co-expression with *Al-otd,* this would be indicative of a role for these *Pax6* genes in specifying the protocerebrum.

**Fig. 6:**
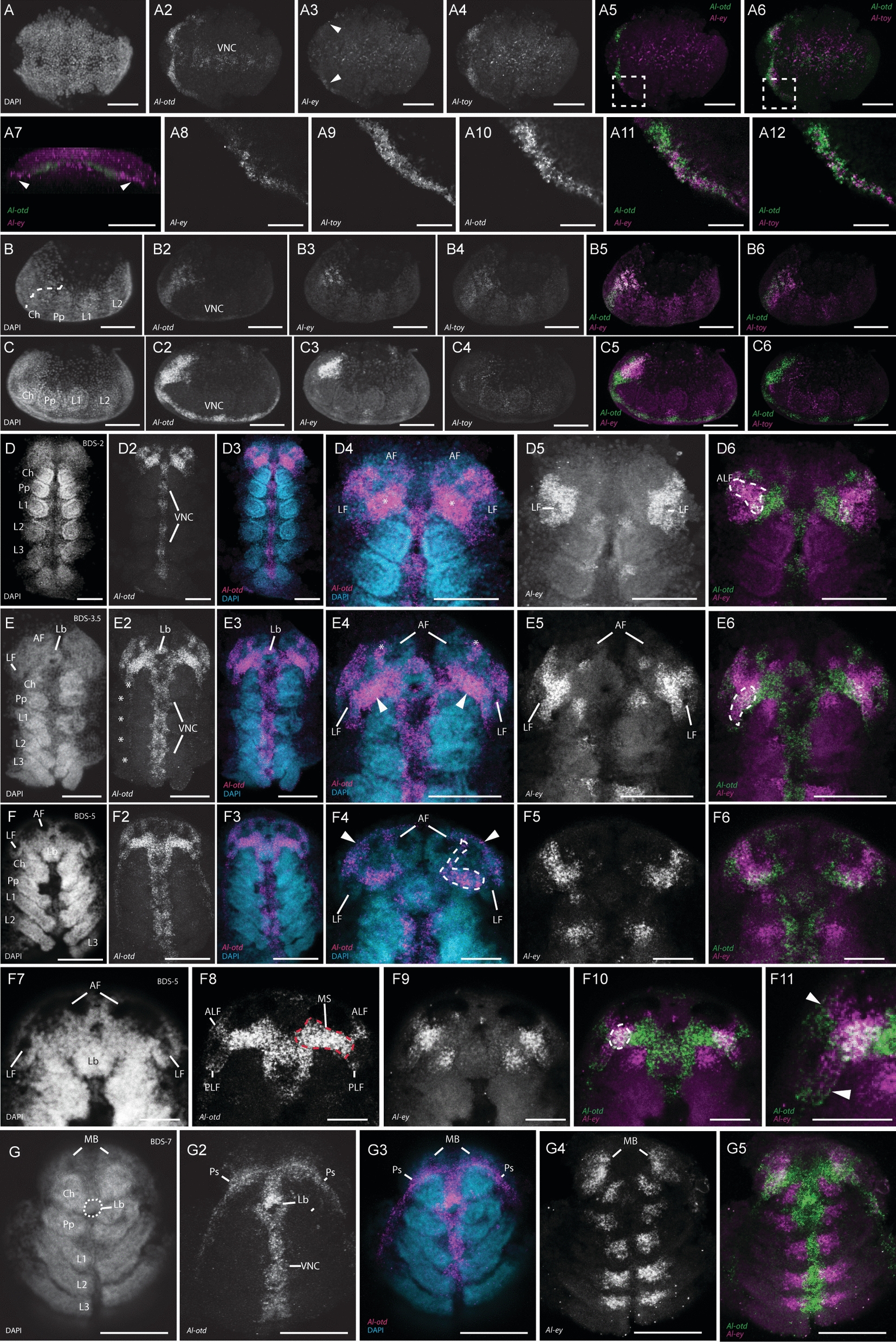
The co-expression of the *Al-Pax6* orthologs with the head-patterning gene *orthodenticle*. **A-A6** *Al-Pax6* co-expression with *Al-orthodenticle* (*Al-otd*) in an early, pre-segmental embryo. The ventral portion of the embryo is shown. **A** DAPI counterstain of this embryo. **A2** *Al-otd* expression in this embryo in a continuous stripe of expression in the embryonic anterior (left of the page). Also, *Al-otd* is weakly expressed in cells of the incipient ventral nerve cord (VNC). **A3** *Al-ey* expression is restricted to two, lateral domains in the anterior rim of the embryo. **A4** *Al-toy* is also expressed in two, paired domains in the anterior of the embryo. **A5** Merged image of the *Al-otd* (green) and *Al-ey* (magenta) channels. **A6** Merged image of the *Al-otd* (green) and *Al-toy* (magenta) channels. Dotted boxes in these images represent the fields of view shown in A8-A12. **A7** Three-dimensional projection of the same embryo, rotated to show the frontal-most expression of *Al-otd* (green) and *Al-ey* (magenta; arrowheads) in the anterior. The embryo is oriented with the ventral towards the top of the page. **A8-A10** Single channel confocal images of *Al-ey*, *Al-toy*, and *Al-otd*, respectively, in the region of the dotted boxes outlined in A5 and A6. **All** Confocal slice of the region of the embryo outlined in the dotted box in A5. **Al2** Confocal slice of the region of the embryo outlined in the dotted box in A6. A11-A12 show that both *Al-Pax6* orthologs are co-expressed with *Al-otd* in this region. **B-B6** *Al-Pax6* and *Al-otd* expression in an early, three prosomal segmented staged embryo. **B** DAPI nuclear counterstain. **B2** *Al-otd* expression in the developing head. Expression also persists in the VNC. **B3** *Al-ey* expression. **B4** *Al-toy* expression. **B5** Merged confocal channels of *Al-otd* (green) and *Al-ey* (magenta) expression. **B6** Merged confocal channels of *Al-otd* (green) and *Al-toy* (magenta) expression. Note that this is the same embryo shown in Fig. 3B-B4. **C-C6** *Al-Pax6* and *Al-otd* expression in an early, limb-bud stage embryo. **C** DAPI nuclear counterstain. **C2** *Al-otd* expression. **C3** *Al-ey* expression. **C4** *Al-toy* expression. **C5** Merged confocal channels of *Al-otd* (green) and *Al-ey* (magenta) expression. **C6** Merged confocal channels of *Al-otd* (green) and *Al-toy* (magenta) expression. **D-D6** *Al-ey* and *Al-otd* expression in an embryo at approximately stage BDS-2. **D** DAPI nuclear counterstain. **D2** *Al-otd* expression. **D3** Merged confocal channels of *Al-otd* (magenta) and the DAPI counterstain (cyan). **D4** The same merged image shown in D3, zoomed in on the developing pre-cheliceral region. Asterisks demark the “blocks” of *Al-otd* expression that surrounds the incipient stomodaeum. LF = the sites of the developing lateral furrows. **D5** Image showing *Al-ey* expression in this embryo. **D6** The same embryo, however the channels showing *Al-otd* expression (green) and *Al-ey* expression (magenta) have been merged. The dotted line outlines the co-expression of these genes in the anterior lateral furrows. **E-E6** *Al-ey* and *Al-otd* expression in an embryo at approximately stage BDS-3.5. **E** DAPI nuclear counterstain. **E2** *Al-otd* expression. The asterisks demark the horizontal lines of expression at the proximal-most boundary of each prosomal appendage. **E3** Merged confocal channels of *Al-otd* (magenta) and the DAPI counterstain (cyan). **E4** The same merged image shown in E3, zoomed in on the developing pre-cheliceral region. Arrowheads mark the “blocks” of *Al-otd* expression. Asterisks mark expression in the lateral margins of the anterior furrows. **E5** Image showing *Al-ey* expression in this embryo. **E6** The same embryo, however the channels showing *Al-otd* expression (green) and *Al-ey* expression (magenta) have been merged. The dotted line outlines the co-expression of these genes in the ventral-most portion of the lateral furrows. **F-F11** *Al-ey* and *Al-otd* expression in an embryo at approximately stage BDS-5. **F** DAPI nuclear counterstain. **F2** *Al-otd* expression. **F3** Merged confocal channels of *Al-otd* (magenta) and the DAPI counterstain (cyan). **F4-F6** Confocal slices of the same embryo, however the slices were taken more dorsally in the embryo. **F4** The same merged image shown in F3, zoomed in on the developing pre-cheliceral region. Arrowheads point to *Al-otd* expression in the margins of the head lobes in the presumptive non-neural ectoderm. *Al-otd* expression in one the medial subdivisions are outlined with a dotted line. **E5** Image showing *Al-ey* expression in this embryo. **F6** The same embryo, however the channels showing *Al-otd* expression (green) and *Al-ey* expression (magenta) have been merged. **F7-11** The same embryo, however the images were taken at a more “surface-level”, or ventral position of the embryo in comparison to (**F4-F6**). **F7** DAPI counterstain at this position. **F8** *Al-otd* expression at this level, showing its expression relative to the anterior lateral furrows (ALF) and the posterior lateral furrows (PLF). MS = expression in the medial subdivision. The right-most MS is outlined in a red, dotted line. **F9** *Al-ey* expression at this position. **F10** Merged image showing *Al-otd* expression (green) and *Al-ey* expression (magenta). Note the co-expression of these genes in the lateral component of the medial subdivisions. The left one is outlined in a dotted line. **F11** The same image shown in F10, however it is zoomed into one of the lateral furrows. Arrowheads point to the boundaries between *Al-ey* and *Al-otd* expression. **G-G5** *Al-ey* and *Al-otd* expression in an embryo at approximately stage BDS-7. **G** DAPI nuclear counterstain. The dotted outline shows the position of the labrum which has been obscured by the movements of the pre-cheliceral region at this stage. **G2** *Al-otd* expression in this embryo, showing its expression in the lateral margins of the two halves of the prosomal shield (PS). **G3** Merged confocal channels of *Al-otd* (magenta) and the DAPI counterstain (cyan). **G4** Image showing *Al-ey* expression in this embryo. **G5** The same embryo, however the channels showing *Al-otd* expression (green) and *Al-ey* expression (magenta) have been merged. Embryos shown in (**B-C6**) are oriented with their anterior pointing towards the left of the page, and their dorsal regions towards the top of the page. All subsequent images are oriented so that the anterior of the embryos are directed towards the top of the page. All scale bars represent 50 µm, except as follows: **A8-A12**, 10 µm; **F4-F11**, 20 µm. All other abbreviations are the same as in other figures

The earliest expression of *Al-otd* that we observed was in early germ-band embryos, where it was expressed in a continuous domain in the embryonic anterior, as well as in the cells incipient ventral nerve cord (= VNC, Fig. 4A-A2). This anterior *Al-otd* domain overlapped with the bilateral *Al-ey* expression domain at this stage, specifically at the lateral margins of *Al-otd* expression (Fig. 6A3, A5, A7, A8, and A11). Furthermore, *Al-otd* was expressed in the same cells as *Al-toy* at this stage. This co-expression with *Al-otd* was more extensive than with *Al-ey* (Fig. 6A4, A6, A9, and A12). This early expression of *Al-toy* extended more medial-ventrally than that of *Al-ey*, where it overlapped *Al-otd* expression in all but the medial domains of *Al-otd* (Fig. 6A4 and A6). Taken together, these early expression patterns of *Al-ey, Al-toy*, and *Al-otd* are similar to those observed in the spider *P. tepidariorum* at roughly its Stage 8 during the early development of the ocular region [93, 94].

In a subsequent germ-band stage, at which the first four prosomal segments had been delineated (*i.e.*, the cheliceral, pedipalpal, and first two walking leg segments), *Al-otd* expression was retained in the incipient ventral nerve cord, as well as the ocular region (Fig. 6B2). *Al-ey* was co-expressed with *Al-otd* at this stage in its lateral, triangular domains of expression (Fig. 6B3). *Al-toy* was also co-expressed with *Al-otd* at this stage, however *Al-toy*’s expression domain was much broader and fully encompassed the domain of *Al-otd* where it extended more posteriorly in the ocular region (Fig. 6B4 and B6).

As shown in Fig. 5, *Al-toy* expression dramatically decreases in the ocular region following its initial expression. Approximately at the stage when this decrease began, and when the prosomal limb buds became more distinct, *Al-otd* retained its expression domain in the ocular region where it was still co-expressed with *Al-ey* (Fig. 6C2, C3, and C5). However, *Al-toy* was not observed to be co-expressed with *Al-otd* at this stage (Fig. 6C6). We take these data to be consistent with a role for both *Pax6* genes and *Al-otd* acting together to specify the neural field of the incipient protocerebrum.

### Late expression of *Al-otd* suggests the ancestral role of *orthodenticle* in arachnids

It was also shown that both the duplicated spider *otd* paralogs, as well as the singleton *otd* ortholog in the opilionid *P. opilio*, are expressed in the developing brain and eyes at developmental stages following the establishment of the head [9, 10, 35, 36, 93]. We thus utilized these data to ask how the spider *otd* paralogs were potentially subfunctionalized, or neo-functionalized, in the lineage leading to spiders.

We began these observations at BDS-2, when the morphology of the ocular region becomes more complex (note that *Al-otd* is expressed broadly at BDS-1 in a manner similar to Fig. 4C5; not shown). At this stage, we detected *Al-otd* expression in the ocular region as well as its retention in the developing ventral nerve cord (Fig. 6D-D3). Within the ocular region, *Al-otd* was expressed in the anterior and posterior margins of the nascent lateral furrows. Note that *otd2* is also expressed in the lateral furrows of the spider *P. tepidariorum* [10, 34], as is *otd* in the opilionid *P. opilio* [36]. *Al-otd* was also expressed in connected domains that span the central to the lateral ocular region (Fig. 6D4). We also detected *Al-otd* expression in two “blocks” of cells surrounding the site of the future stomodaeum (Fig. 6D4, asterisks) in a similar manner to *otd* in *P. opilio* [36] and *otd2* in spiders [34]. We likewise co-stained these embryos for *Al-ey* expression and found that *Al-otd* and *Al-ey* expression overlap in the anterior of the lateral furrows (note that *Al-toy* expression is absent in the ocular region at this stage). However, they do not overlap at the posterior portion of the lateral furrows (Fig. 6D6, the left anterior portion of the lateral furrow, ALF, is outlined).

At approximately BDS-3.5, *Al-otd* expression was still present in the developing ventral nerve cord (Fig. 6E-E2). Faint *Al-otd* expression was also detected in horizontal lines of expression at the proximal-most boundary of each prosomal appendage (Fig. 6E2, asterisks). This appendicular expression may be homologous to that of *Pt-otd2* expression at later stages (*i.e.*, stages 12 and 13; see figs. 11H-I in [10]) and in the opilionid *P. opilio* ([36], their Fig. S2). Within the ocular region, *Al-otd* expression was detected in the developing labrum (Fig. 6E-E3), in a similar manner to spider *otd2* orthologs [9, 34] and to the expression of *otd* in *P. opilio* [36]. *Al-otd* expression was subsequently retained in the “blocks” of cells surrounding the stomodaeum (Fig. 6E4, arrowheads), and was also detected in the lateral margins of the anterior furrows (Fig. 6E4, asterisks). By co-staining for *Al-ey* expression, we were able to detect its co-expression with *Al-otd* expression in the ventral-most portion of the lateral furrows (Fig. 6E6, dotted outline).

At BDS-5, the expression of *Al-otd* in the ventral nerve cord remained, and the proximal appendicular expression domains became more pronounced. Furthermore, each of these domains appeared to combine to become continuous with one another on their respective half of the embryo (Fig. 6F-F3) like *otd* expression at stages 12–15 in *P. opilio* [36]. Also, in a similar manner to the spider *otd2* orthologs [9, 34], and the *P. opilio otd* ortholog [36], we additionally detected *Al-otd* expression in the margins of the head lobes in the presumptive non-neural ectoderm (Fig. 6F4, arrowheads). Also, *Al-otd* expression surrounded the periphery of the fused labrum (Fig. 6F-F3).

Recall that at this stage, the medial subdivisions send out “extensions” of cells that will separate the anterior furrows from the nascent mushroom bodies (Fig. 1E-E2). We detected *Al-otd* expression in these medial subdivision extensions (Fig. 6F4; the right-most medial subdivision’s extension is outlined). Because of their proximity to the persisting “blocks” of *Al-otd* expression surrounding the labrum at this stage, we take the “blocks” of *Al-otd* expression in BDS-2 and BDS-4 to be the medial subdivisions. We also detected the co-expression of *Al-ey* and *Al-otd* within a population of cells in the lateral halves of these medial subdivisions (Fig. 6F8-11; one of these populations is outlined in F10). These patterns are like those of spider *otd2* expression [34] and to *P. opilio otd* expression [36], suggesting a high degree of conservation of *otd* expression in these precursors.

Because *Al-ey* at this stage is also expressed in the ventral-most half of the lateral furrows (see Fig. 4F-F3), we asked to what extent *Al-otd* and *Al-ey* are co-expressed in these “open” lateral furrows. By co-detecting *Al-ey* with *Al-otd*, we found that their expression is mutually exclusive in the anterior lateral furrows, with *Al-otd* expression located more “inwardly” in the lateral furrow compared to *Al-ey* expression (Fig. 6F10-11; arrowheads in F11 point to the boundaries between *Al-ey* and *Al-otd* expression). These expression patterns are interesting, as they may point to a coordinate-type system to establish polarity, or a mechanism of regionalization, in the lateral furrows. Lastly, both *Al-ey* and *Al-otd* were co-expressed in the ventral-lateral cells that are adjacent to the lateral furrows (Fig. 6F10-11; these cells are outlined in the left side of the embryo in F10).

At approximately BDS-7, when the prosomal shield halves had migrated and fused, we observed *Al-otd* expression in the margins of the fused prosomal shield. This expression domain was continuous to the lateral appendicular expression domains of the previous stages (Fig. 6G-G3). A similar colorimetric staining pattern was observed for *otd2* orthologs in the spider species studied in [34], however the authors described these as the results of artefactual cuticle staining. Interestingly, we did not observe similar staining to that shown in Fig. 6G-G5 in our control experiments, nor in any other HCR experiments of other genes. Furthermore, *P. opilio otd* is expressed in a similar pattern at stages 12–15 [36]. Therefore, we take this expression pattern to be a conserved mode of *otd* expression among arachnids.

*Al-otd* expression was also present in the ventral nerve cord and the periphery of the labrum at BDS-7 (Fig. 6G-G3). Within the ocular region, we did not observe *Al-otd* in any of the tissues in which it was expressed in the previous stages. This was shown via the co-staining of *Al-otd* with *Al-ey*, which is expressed largely in the mushroom bodies at this stage (Fig. 6G4-G5). We did not observe expression of *Al-otd* at any subsequent pre-larval or larval stages.

## Discussion

### The morphogenesis of the arachnid head and brain in light of *A. longisetosus*

**Fig. 7:**
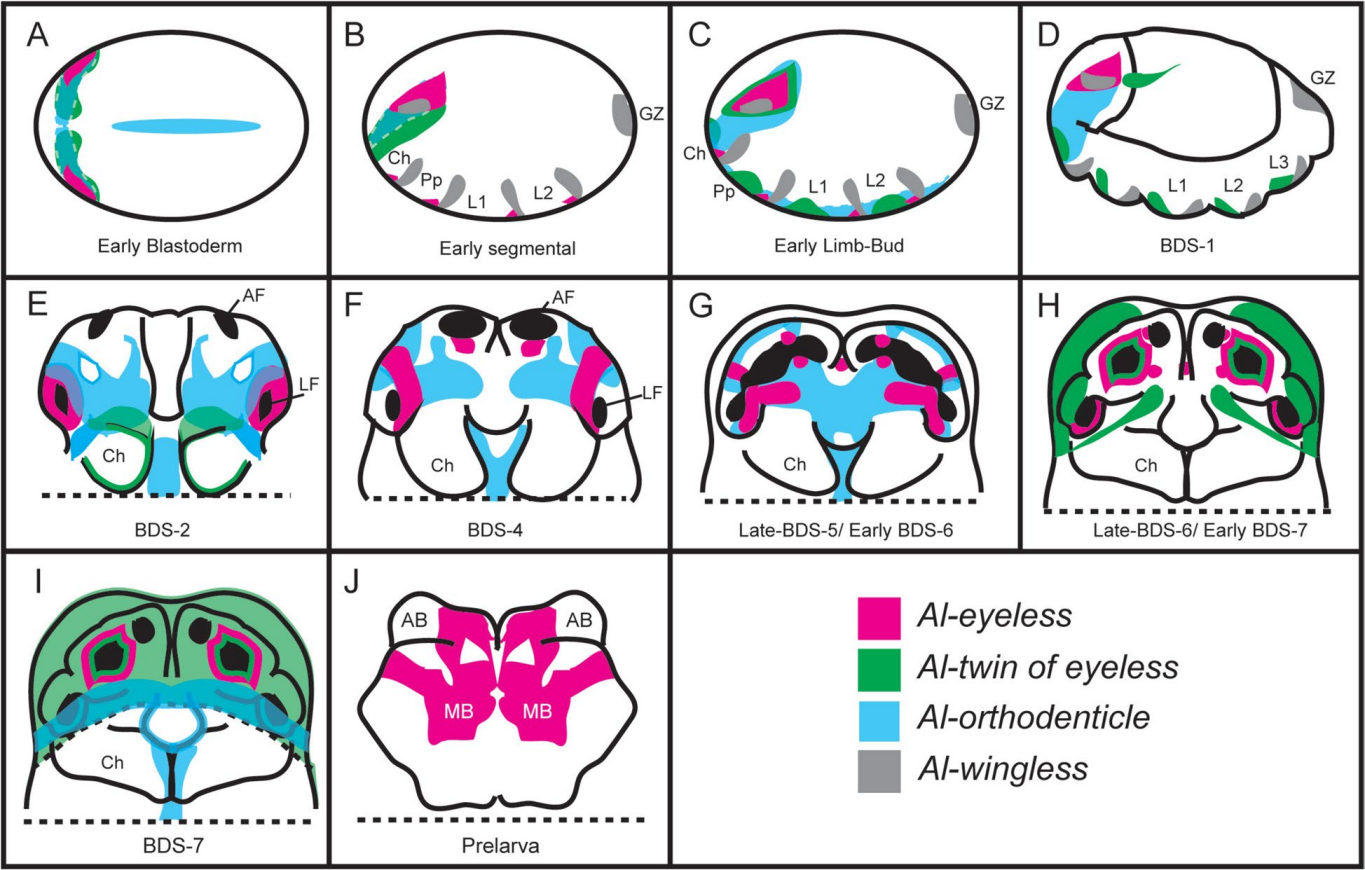
Summary drawings showing the relative expression patters of the *Al-Pax6* orthologs and *Al-otd* throughout the development of the pre-cheliceral region. See text for details

Modern studies into the development of the arachnid head have largely focused on the spiders *P. tepidariorum* (*e.g*., [73]) and *C. salei* (*e.g*., [71, 72]). These studies, in conjunction with recent studies on the development of the opilionid *P. opilio* [36, 69], have revealed potential shared features of arachnid head development. These include the appearance of the anterior and lateral furrows, followed by the migration of the non-neural prosomal shield over the ocular region. Despite the conservation of these features in *A. longisetosus*, our data show major morphogenetic divergences between mites and other arachnids in the embryonic ocular region (Fig. 7).

First, we observed potential differences in the timing of the appearance of the lateral and anterior furrows. In spider exemplars, the lateral furrows form first, followed by the anterior furrows [35, 72, 73]. This may also be the order of appearance in *A. longisetosus*; however, with our methods, we were only able to visualize the appearance of both pairs of furrows simultaneously at BDS-2. In *P. opilio*, it is also unclear as in what order these furrows appear, as they also seem to appear simultaneously (see fig. 8C in [12]). Live imaging of mite and opilionid development is needed to explore this hypothesis further.

Another point of divergence between spiders and *A. longisetosus* can be seen in the later morphogenesis of the anterior and lateral furrows. In spiders, the division of the anterior furrows into the mushroom and arcuate bodies occurs through the migration of the medial subdivisions expanding into the anterior furrows, which results in the compartmentalization of the arcuate and mushroom bodies [71–73]. Our observations suggest that this aspect of brain morphogenesis is conserved between mites and spiders, with one major exception. In these spider species, the anterior furrows do not appear to make continuous grooves with the lateral furrows at any stage. In *A. longisetosus*, however, the lateral and anterior furrows become continuous with one another after their initial appearances as distinct structures (see Fig. 1). These continuous grooves are then subsequently subdivided to form the arcuate bodies, mushroom bodies, and the optic vesicles, with the arcuate and mushroom bodies delimited by extensions of the medial subdivisions. A secondary, and yet unnamed, group of cells forms later to separate the posterior of the mushroom bodies from the anterior of the lateral furrows.

One final point of divergence was seen in the development of the lateral furrows. In spiders, the lateral furrows are further subdivided through the expansion of another grouping of paired elevated neural tissues called the lateral subdivisions. These lateral subdivisions subdivide the lateral furrows into the lateral and medial optic vesicles [9, 10, 33–36, 71]. Our observations of *A. longisetosus* brain compartmentalization did not show evidence of any lateral subdivisions. Interestingly, it has been proposed that it is these lateral vesicles that give rise to the optic ganglia of the lateral eyes of spiders [71]. It is therefore tempting to attribute the absence of the lateral subdivisions in *A. longisetosus* to their absence of eyes. However, to test this hypothesis, the compartmentalization of the brains of acariform mites that have retained their eyes needs to be studied.

Taken together, our results suggest that presence of paired anterior and lateral furrows as well as the migration of the prosomal shield are conserved aspects of arachnid brain/ocular region development. Despite this, the subsequent subdivisions of these furrows may be lineage-specific. An alternative explanation for our observations could be that the mode of brain compartmentalization in *A. longisetosus* is highly derived within Acariformes. To clarify this, more studies into the brain development of members of this hyper-diverse clade are needed. Also, given that *A. longisetosus* lacks eyes, the presence of the lateral furrows that give rise to the optic vesicles needs an explanation. The simplest explanation is that these compartments of the brain do not solely give rise to the optic neuropils as they do in spiders, and they therefore may contribute to other important components of the brain. Alternatively, these lateral furrows could be vestiges of the optic neuropils of true acariform eyes. This latter hypothesis could support an example of “developmental burden”, an evolutionary concept developed by Rupert Reidl [95, 96]. Briefly, developmental burden is the concept that if some characteristic of an organism has many important functions and/or there are many other characters that depend on its function, then that character will be less likely to undergo evolutionary change. The retention of the lateral furrows and the resulting optic vesicles could thus be a product of their interconnectedness with other neural structures and/or functions. Cellular lineage tracing experiments, which are not yet available for this species, are needed to further explore these hypotheses.

### Ocular RDGN expression in *A. longisetosus* in comparison to other arachnids and chelicerates

Our RDGN expression results are the first to be described for an acariform mite. These data provide an opportunity to explore similarities and differences among their expression in arachnids. Furthermore, these data also allow for the potential to identify common targets for eye loss across arachnid exemplars. Below we discuss our HCR results in comparison to other studied chelicerates.

#### Arachnid *eyes absent (eya)*

Unlike many of the RDGN genes, only a single copy of *eya* has been recovered in all surveyed spider taxa [34, 35]. In the developing head of the spiders *P. tepidariorum* and *C. salei*, *eya* was expressed in the non-neural margins of the head lobes of early embryos. In each lobe, *eya* was detected in two separate domains, *i.e.*, an anterior and posterior domain. As the prosomal shields migrated, *eya* expression was enriched in the edges of the prosomal shield, and these cells were taken to be the primordia of the eyes. Upon the completion of prosomal shield migration, *eya* was expressed in all eye types of *P. tepidariorum* [10]. However, in *C. salei*, *eya* was only expressed in the secondary eyes (*i.e.,* all eye types to the exclusion of the anterior-median eyes) [9]. This may be specific to *C. salei*, as a phylogenetic survey of a wide range of spider species showed that their *eya* orthologs are also expressed in all eye subtypes [34]. In a recent HCR screen of RDGN genes in the cave spider *T. pagana*, its *eya* ortholog was also embryonically expressed in all the eye anlagen [35].

Outside of spiders, the only other chelicerate in which the embryonic expression of *eya* was surveyed was in the opilionid *P. opilio.* Like spiders, the earliest expression pattern of *Po-eya* was in the lateral margins of the head lobes. *Po-eya* was later expressed in the developing rims of the lateral furrows as well as in the anterior furrows. This observation is interesting, as it was reported that there was no *eya* expression in the lateral furrows in the spider *P. tepidariorum* [10] nor in *C. salei* [9]. With the aid of high-resolution imaging of *Po-eya* expression, the authors were able to distinguish between non-neural and neural *Po-eya* expression. *Po-eya* appeared to be expressed in both the anlagen of the median and vestigial lateral eyes in the non-neural ectoderm as well as in the adjacent neural ectoderm. As the prosomal shield migrated, *Po-eya* expression was observed in the developing median and vestigial lateral eyes [36]. Outside of eye and brain development, the aforementioned studies on spiders and opilionids showed *eya* expression in the labrum, stomodaeum, segmental clusters of the ventral central nervous system, and in what appears to be the mesoderm of the appendages.

Our *Al-eya* expression patterns are very similar to those mentioned above for other arachnids up to later stages (BDS-7, Fig. 2F-F3). The presence of *Al-eya* in the margins of the ocular lobes is likely non-neural, as its expression is very similar to its expression in arachnids that have eyes, and thus we take this non-neural expression to likely be early eye tissue. However, we acknowledge that there may also be neural *Al-eya* expression, as seen in *P. opilio* [36]. To date, there is no specific genetic marker for the non-neural ectoderm of the ocular region in chelicerates. Current methods that could reveal such a marker include single-cell RNAseq, which has already been used to discover novel developmental genes in *P. tepidariorum* [93, 97–99]. This method is being planned for *A. longisetosus* (AAB, pers. comm.), and could reveal such a marker for the non-neural ectoderm of the ocular region. Nevertheless, we take our evidence as support for the hypothesis that *A. longisetosus* begins to develop eye primordia; however, *Al-eya* expression is subsequently downgraded. This is interesting, as the comparative RNAseq study of an eyed and a whip spider with reduced eyes showed that the single *eya* ortholog is upregulated in embryos from the reduced-eyed species (*i.e., Charinus israelensis*) [56]. Therefore, natural selection may target *eya* genes for eye-reduction differently across arachnid taxa. Reverse genetic techniques, such as RNA interference (RNAi) or CRISPR, are needed to clarify this hypothesis; however, these methods are not yet available for *A. longisetosus*, although they are being explored (AAB, pers. comm.).

#### Arachnid *sine oculis* (*so*) expression

Like many of the genes in the RDGN, all surveyed spider species retain two paralogs of *so* in their genomes [9, 34]; however, the expression of these paralogs in the development of eyes varies across spiders. In all spider taxa investigated in [34], *so1* was shown to be expressed in all eye subtypes. A notable exception to this was the usage of *so1* in *C. salei* (= *Cs-six1a*), which was expressed in all eye subtypes to the exclusion of the anterior-median eyes [9]. The expression of the second paralog, *so2*, was present in all eye subtypes in most of the species examined by [34]. However, in the two species of spiders that belong to the clade Synspermiata, *so2* expression was not observed in any of the eye anlagen. The variability of *so2* usage in eye development was also demonstrated by its expression in only the anterior-lateral eyes of *P. tepidariorum*. Lastly, the *C. salei so2* ortholog (= *Cs-six1b*) was expressed in all eye subtypes, to the exclusion of the anterior-lateral eyes [9]. In the cave spider *T. pagana,* its *so1* paralog was expressed in all eye subtypes (note that the *so2* paralog was not studied). The only functional study of an arachnid *sine oculis* paralog was done on *P. tepidariorum.* This study showed that targeted RNAi against this gene resulted in the loss of all eye subtypes [56]. Outside of spiders, the single copy of *so* in the opilionid *P. opilio* was expressed in the medial eyes as well as in the vestigial lateral eyes [36].Taken together, *sine oculis* genes in arachnids are likely needed to specify eyes in arachnids.

In *D. melanogaster*, *so* interacts directly with *eya* [83], and both are expressed similarly in ﻿Araneomorph spiders [9, 10, 33, 35]. *Al-eya* and *Al-so* are also similarly expressed with one another (*e.g*., compare Fig. 2D3 to H3). This suggests that there is a conserved interaction between the products of both genes that may also be conserved in arachnids to specify eyes. Therefore, we hypothesize that the expression of *Al-so*, as with *Al-eya*, is likely a remnant of early eye specification. Further support for this is the downregulation of both genes seen at later stages (*i.e.*, BDS-7).

#### Arachnid *dachshund* expression

As with most of the RDGN genes, spiders have two paralogs of *dac*. The expression of these paralogs in spider eyes seems to be clade-specific; however, in each species surveyed, at least one *dac* paralog is expressed in an embryonic eye [34, 35]. In *P. opilio*, *Po-dac* is expressed in the medial eyes as well as the vestigial lateral eyes. Furthermore, RNAi targeting *Po-dac* results in the absence of the lateral eyes, without affecting the median eyes [36]. Our data show a lack of *Al-dac* in the embryonic ocular region; however, it is expressed in the prelarval mushroom and arcuate bodies. Because *dachshund* acts downstream of *so* and *eya* in the RDGN of *Drosophila* [85, 86], our results are consistent with the hypothesis that some developmental gene may be inhibiting the action of *Al-eya* and *Al-so* in activating *Al-dac* in the ocular region. This does, of course, assume that this molecular interaction is conserved in arachnids. Therefore, functional comparisons are needed between *A. longisetosus* and arachnids with eyes to test this hypothesis further.

#### Arachnid *Six3/Optix* expression

Spiders have two paralogs of *Six3*, and in most spider species, one or both paralogs are expressed in at least one of the eye anlagen, except for the eyes of the spiders *A. geniculata* and *P. phalangioides* [34, 35]. In the daddy-longlegs *P. opilio, Six3* is expressed in the developing median eyes [36]. Our data are consistent with other arachnid and arthropod taxa which show *Six3* expression in the central complex/arcuate bodies of the ocular region. However, we did not observe comparable expression patterns in the ocular region that would suggest vestigial eye primordia. Interestingly, an RNAseq study in the whip spider *C. israelensis* that has reduced eyes showed that the *Six3* paralog *OptixA* was upregulated during embryogenesis [56]. This suggests, like the upregulation of *eya* in this species, that the convergent reduction of eyes between this whip spider and *A. longisetosus* suggests a different mode of eye repression.

We did observe *Al-Six3* expression in the lateral furrows (Fig. 2B-D3), which later develop into the optic vesicles. The paralog *Six3.2* was not expressed in the lateral furrows of *T. pagana* [35] (note that *Six3.1* was not followed in this study). In *C. salei*, *Six3a* is expressed in the lateral furrows [9] as is *Six3.1* in *P. tepidariorum* [10]. It is unclear what the *Six3*-positive cells in these species develop into, however these data are consistent with the hypothesis that *Six3* genes pattern some ancestral aspect of the arachnid lateral furrows.

#### Chelicerate *atonal* expression

*Atonal* expression has been followed in a non-arachnid chelicerate, the horseshoe crab *L. polyphemus*. In this study, it was revealed that its *atonal* ortholog is not expressed in any of the developing eye primordia [32]. In spiders, however, the *ato1* paralog seems to have a conserved expression domain in the anlagen of all eye subtypes, with the only exception being the spider *Segestria senoculata*, a member of the Synspermiata [34]. The same study also provided evidence that supports the hypothesis that the *ato2* paralog was ancestrally expressed in the primary eye primordia, as it is expressed solely in these tissues in all taxa it surveyed [34]. In the spider *C. salei*, neither of its two *atonal* orthologs are expressed in the embryonic eye vesicles, however both are expressed in the lateral furrows [9]. In the spider *T. pagana*, the only paralog studied (*atonal 1*) was expressed in the anterior furrows and the medial subdivisions, and subsequently in the anlagen of all eyes [35].

We did not observe any *Al-ato* expression in the ocular region that would be indicative of vestigial eye formation, *i.e.,* in the non-neural ectoderm of the head. In fact, *Al-ato* expression at this stage was most similar to the expression of the *ato2* paralog of the spider *P. tepidariorum* [33]. *Pt-ato2* was shown to be expressed in the ocular region in two neuroectodermal clusters like our observations of *Al-ato* (see fig. 5I in [33]). In summation, these data show that the use of atonal to pattern eye tissue may be specific to spiders. Thus, further study into the expression and utilization of *atonal* orthologs in non-spider chelicerates are needed to explore this hypothesis further.

#### Chelicerate *eyeless* expression

Our data show both shared and derived modes of *eyeless* expression in arachnids. We found that early *Al-ey* expression is similar to that of spiders, specifically in comparison to early *P. tepidariorum eyeless* expression [10, 34, 93]. In both taxa, *eyeless* is expressed early in an anterior domain in the ocular region. Furthermore, our data highlight other conserved aspects of arachnid *eyeless* expression. We observed its expression in the nascent mushroom bodies and the optic vesicles. *eyeless* expression was similarly observed in spiders [9, 10, 34, 93], and may also be expressed in these structures in *P. opilio* [36].

By evaluating *eyeless* expression patterns reported for other taxa, we conclude that in spiders, in an opilionid and in *A. longisetosus* (Fig. 2), *eyeless* is subsequently expressed in cells associated with the lateral furrows. Because the lateral furrows likely develop into the optic vesicles in spiders [71], the usage of these optic vesicles in arachnids lacking eyes should be a key focus of study to further our understanding of both the arachnid brain, its development, and the function of *eyeless* in patterning these structures. Our results cannot falsify the hypothesis that our observed *Pax6* expression patterns are vestigial, *i.e.*, relictual features of eye development. We are currently exploring methods to abrogate gene expression in *A. longisetosus*; however, we are thus far limited to gene expression surveys. Once methods to test for gene functions are in place, we plan to knock down both *Pax6* genes to test this hypothesis directly. If *Pax6* gene expression is indeed vestigial in *A. longisetosus*, we would expect to see no morphogenetic anomalies in *Pax6*-depleted embryos. Related to this, we cannot thus far falsify the hypothesis that the lateral furrows/optic vesicles are themselves vestigial. Because we do not yet know if, or exactly what, other non-visual roles of the optic vesicles may be, more functional neural studies into these compartments of the arachnid brain are necessary before making this conclusion.

#### Chelicerate *twin of eyeless* expression

Our data also suggest both conserved and derived aspects of *toy* expression among arachnids. To date, early expression data for *toy* in arachnids are limited to the spider *P. tepidariorum* [93]. In both this spider and in *A. longisetosus*, *toy* is expressed in a broad anterior domain in the ocular region. Following this pattern, *Al-toy* expression deviates dramatically from its ortholog’s expression in other arachnids. For instance, *Al-toy* is expressed in the developing prosomal appendages, in a manner not seen in other studied arachnids. Also, *Al-toy* expression disappears from the ocular region at intermediate stages of development. This is in stark contrast to opilionids and spiders, in which *toy* expression persists in the ocular region at comparable stages [34, 36]. We also observed ubiquitous expression of *Al-toy* in the migrating prosomal shield at late stages of development. In the opilionid *P. opilio*, both *Pax6* orthologs appear to be ubiquitously expressed in this structure at later stages [36]. In spiders, neither *Pax6* ortholog appears to be expressed ubiquitously in the prosomal shield [34]. It is tempting to conclude that these deviations in the use of *toy* in *A. longisetosus* are due to their absence of eyes. However, since *toy* does not contribute to eyes in any studied arachnid, the differential use of *toy* in *A. longisetosus* necessitates future functional studies.

#### Arachnid *orthodenticle* expression

The ocular region sensu [37] in all studied arthropods is, in part, defined by the expression of *orthodenticle* orthologs. *orthodenticle* orthologs are expressed in the ocular region, and are also expressed in the anlagen of the eyes in all arachnids surveyed [9, 10, 34–36], aside from *A. longisetosus* (this study). In terms of eye development, one *P. tepidariorum otd* ortholog, *Pt-otd2*, is expressed late in development in the anterior-median eyes [10, 34]. In the spider *C. salei*, both of its *otd* paralogs are expressed in tissues associated with the eyes. Specifically, *Cs-otxa* (= *otd1*) is expressed in the vesicles of the posterior-lateral eyes, whereas *Cs-otxb* (= *otd2*) is expressed in all sets of lateral eyes as well as the posterior median eye vesicles [9]. In a recent, comprehensive study of diverse spider taxa, it was shown that in all the spider species studied, *otd2* orthologs are expressed exclusively in the anterior-median eyes, with the notable exception of a lack of any eye *otd* expression in *Pholcus phalangioides* [34]. In T. pagana, its *otd2* gene was expressed in all eye subtypes (note that *otd1* was not found in their transcriptome) [35]*.* Additionally, a recent study on *P. opilio* revealed that its *otd* ortholog is expressed in all eye primordia [36]. We did not observe similar expression patterns of *Al-otd* in our HCR experiments, further confirming the absence of vestiges of eyes in *A. longisetosus* during embryonic development. In summation, our results, coupled with those observed in *P. opilio* [36]*,* suggest that *Al-otd* expression is most like spider *otd2* ortholog expression in the ocular region [34].

### Eyes in mites

Members of Acari (mites and ticks) display a wide degree of morphological diversity, owing to their occupancy of numerous ecological niches [44, 45]. The monophyly of Acari is currently still contested (*e.g*., [11, 100, 101]); however, it is generally agreed that Acari comprises two internally monophyletic groups, the Parasitiformes (*e.g.,* ticks) and Acariformes (mites). Within Acariformes, the number, position, and types of eyes present are extremely diverse, also owing to their ecological diversity (see [8, 44, 45] for review). Examples of this diversity include the retention of both lateral and median eyes, with varying numbers of each (*e.g.*, the mite *Heterochthonius gibbus* has one median eye and a pair of lateral eyes [102]), or the (likely) parallel loss of all eyes independently in several acariform groups (reviewed in [44, 45]). Notwithstanding, it has been hypothesized that the ground plan for acariform mites is the presence of two median eyes and two pairs of lateral eyes, a condition that was inferred in an early study [103]. However, it has been cautioned that determining the plesiomorphic condition of acariform eyes is a complex problem considering the morphological disparity between acariform sub-clades [8].

Nonetheless, given the wide degree of eye diversity in mites, coupled with the emergence of new developmental data on the expression and function of RDGN genes in arachnids, mites are a clade of extreme interest in terms of exploring the developmental evolution of arachnid eyes. Of specific interest is how the acariform mite *Tetranychus urticae* patterns its eyes. *T. urticae* is a member of Trombidiformes, the sister clade to Sarcoptiformes, of which *A. longisetosus* belongs [44]. *T. urticae* has been shown to be a tractable developmental model (*e.g.* [104–107]), with a published genome [108]. This species also retains two pairs of eyes on each side of its head [109]. It will therefore be valuable to follow RDGN expression in this species to provide a basis for comparison for future studies into mite visual developmental evolution.

Another area of possible research is the role of *Pax2* in the development of the acariform ocular region. In a recent study, it was shown that in the spider *P. tepidariorum*, the *Pax2* paralog *Pax2.1* was expressed in the lateral eye primordia [110]. *Pax2* expression was also observed in all eye primordia in the spider *T. pagana* [35], as well as in all eye primordia of *P. opilio* and of the scorpion *Centruroides sculpturatus* [36]. This suggests that the roles for *Pax6* genes in chelicerates may have been replaced by *Pax2*. It will therefore be interesting to follow the expression of *Pax2* in *A. longisetosus* to potentially identify additional vestigial eye tissues in this eyeless arachnid.

### Eye loss in arachnids

The main motivation for this study was the observation that *A. longisetosus* retains all the component genes of the RDGN that are also expressed in arachnids that have eyes [54]. This paradoxical observation led to the hypotheses that they are either retained due to these genes being utilized in other developmental contexts, being expressed in vestigial eye tissues or structures, or some combination of both.

The above data demonstrate that these genes do take part in the development of both the non-neural and neural tissues of the ocular region and other components of the CNS and may hint at the vestigial development of eye tissue. The expression of both *Al-eya* and *Al-so* in the rims of the ocular region do suggest that these genes may pattern vestigial eye precursors. This hypothesis stems from the observations that RNAi targeting *so* in *P. tepidariorum* led to the loss of all eyes [56], that *atonal* and *so* orthologs are expressed in the embryonic eyes of all surveyed arachnid taxa [9, 10, 34–36], and that the expression patterns of *Al-so* and *Al-eya* are extremely similar to those of all studied Araneomorph spiders at comparable developmental stages [9, 10, 33, 35].

A recent RNAseq study that focused on the differential expression of eye patterning genes between two congeneric species of whip spiders, including one species with eyes (*i.e., C. ioanniticus*) and another with reduced eyes (*i.e., C. israelensis*), showed that its *Pax6A* gene and both of its *Optix* paralogs were upregulated in the species with reduced eyes. Furthermore, the expression of the *orthodenticle* paralog *otd-B* was relatively higher in the species with eyes [56]. These differences in gene expression patterns do not appear to be similar in *A. longisetosus*, suggesting that different genes were the targets for eye loss in *C. israelensis* and *A. longisetosus*. Additionally, one hypothesis to explain our observations could be that some gene or set of genes repress the development of eyes in *A. longisetosus.* An expression survey in the spider *P. tepidariorum* suggested that Wnt signaling may restrict eye development in this species [33]. We plan to test the hypothesis that Wnt signaling plays a role in suppressing eye development in *A. longisetosus* shortly. Nevertheless, it will be interesting to see what genes have been similarly targeted in other eyeless arachnid clades, and future comparative studies could highlight common “dials” in the RDGN that natural selection can turn to lead to the convergent loss of eyes in arachnids.

Another area of future research is to understand the role of the remaining opsin genes that are retained in the *A. longisetosus* genome, nominally *Al-rhodopsin-7* and *Al-peropsin* [54]. Rhodopsin-7 proteins are involved in arthropod circadian photoreception, and Peropsins are non-visual pigments [78, 111–115]. A role for circadian rhythm maintenance could be responsible for the retention of *Al-rhodopsin-7*; however, the role of *Al-peropsin* remains elusive. Peropsin and rhodopsin in arthropods are usually expressed in the eyes (*e.g.,* [78, 113, 115]) which are absent in *A. longisetosus*. Once a protocol for RNAi in *A. longisetosus* is developed, it will be interesting to explore the function of these opsins in our eyeless mite.

## Conclusions

Eye loss is extensive across mite species; however, paradoxically, the eyeless mite *A. longisetosus* retains two *Pax6* paralogs in its genome, as well as other components of the arthropod retinal determination gene network (RDGN). To explore the potential role for these genes, we first described the development of the ocular region of *A. longisetosus and* showed that there are differences in the morphogenesis of the mite ocular region compared to other arachnid exemplars. However, despite these differences, *A. longisetosus* does retain structures that have been implicated in the development of arachnid eyes, *i.e*., the ocular vesicles and the medial subdivisions.

By following the expression of genes canonically associated with arthropod eye development, we found support for the hypothesis that *A. longisetosus* does pattern some early eye precursors. However these never develop into morphologically discernible eyes. Also, our results support the hypothesis that ancestrally, *Pax6* genes worked with *orthodenticle* to specify the neural cells of the protocerebrum independent of *orthodenticle’s* role in eye specification. Lastly, our results suggest that one *Pax6* ortholog, *eyeless*, was likely used ancestrally in chelicerates to specify the paired optic vesicles of the chelicerate brain as well as the mushroom bodies. We also found evidence that the role for the *Pax6* paralog, *twin of eyeless*, is involved in the formation of the appendages as well as in the prosomal shield.

## Supplementary Information

Additional file 1.

Additional file 2.

Additional file 3.

Additional file 4.

Additional file 5.

Additional file 6.

Additional file 7.

Additional file 8.

Additional file 9.

Additional file 10.

Additional file 11.

Additional file 12.

Additional file 13.

Additional file 14.

Additional file 15.

Additional file 16.

Additional file 17.

## Data Availability

No datasets were generated or analysed during the current study.
