# Supplementary figures and images for: The expression of Pax6 and retinal determination genes in the eyeless arachnid A. longisetosus reveals vestigial eye primordia
Source: EvoDevo. 2025 Jul 9;16:12. doi: 10.1186/s13227-025-00245-7 (PMC12239259; doi:10.1186/s13227-025-00245-7)

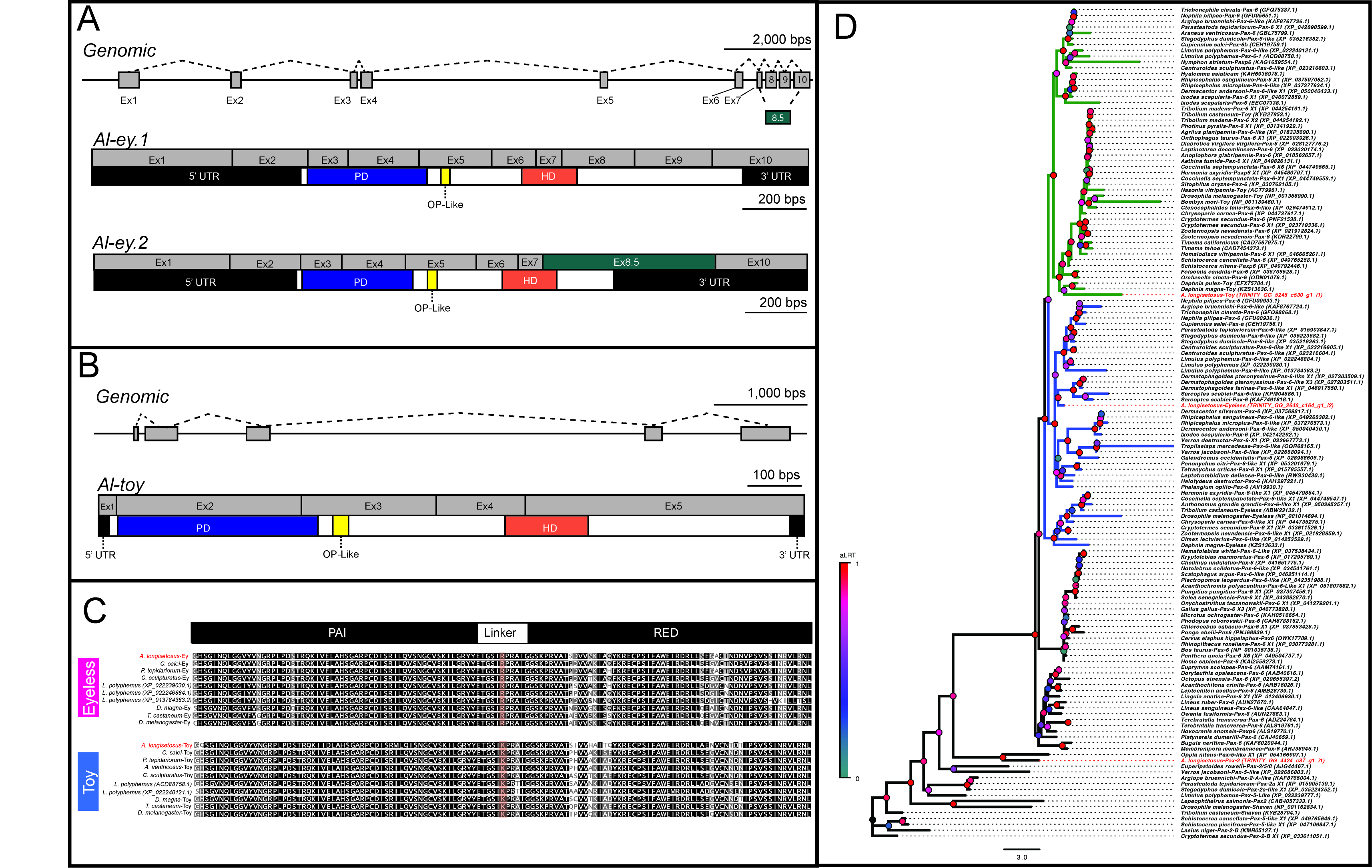

Supplement: Supplementary file 1 — Additional file 1. [file 13227_2025_245_MOESM1_ESM.tif]

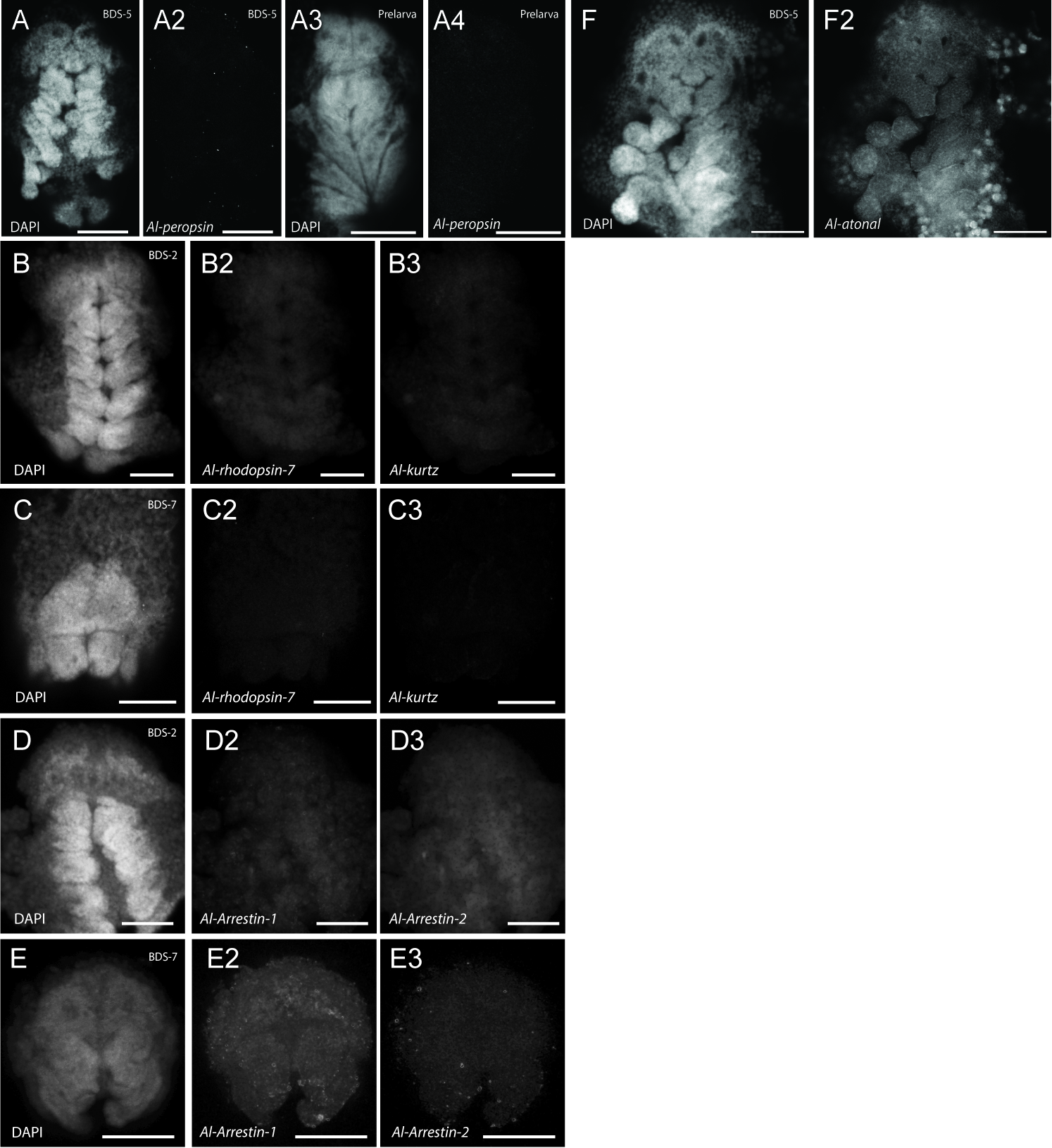

Supplement: Supplementary file 16 — Additional file 16. [file 13227_2025_245_MOESM16_ESM.tif]

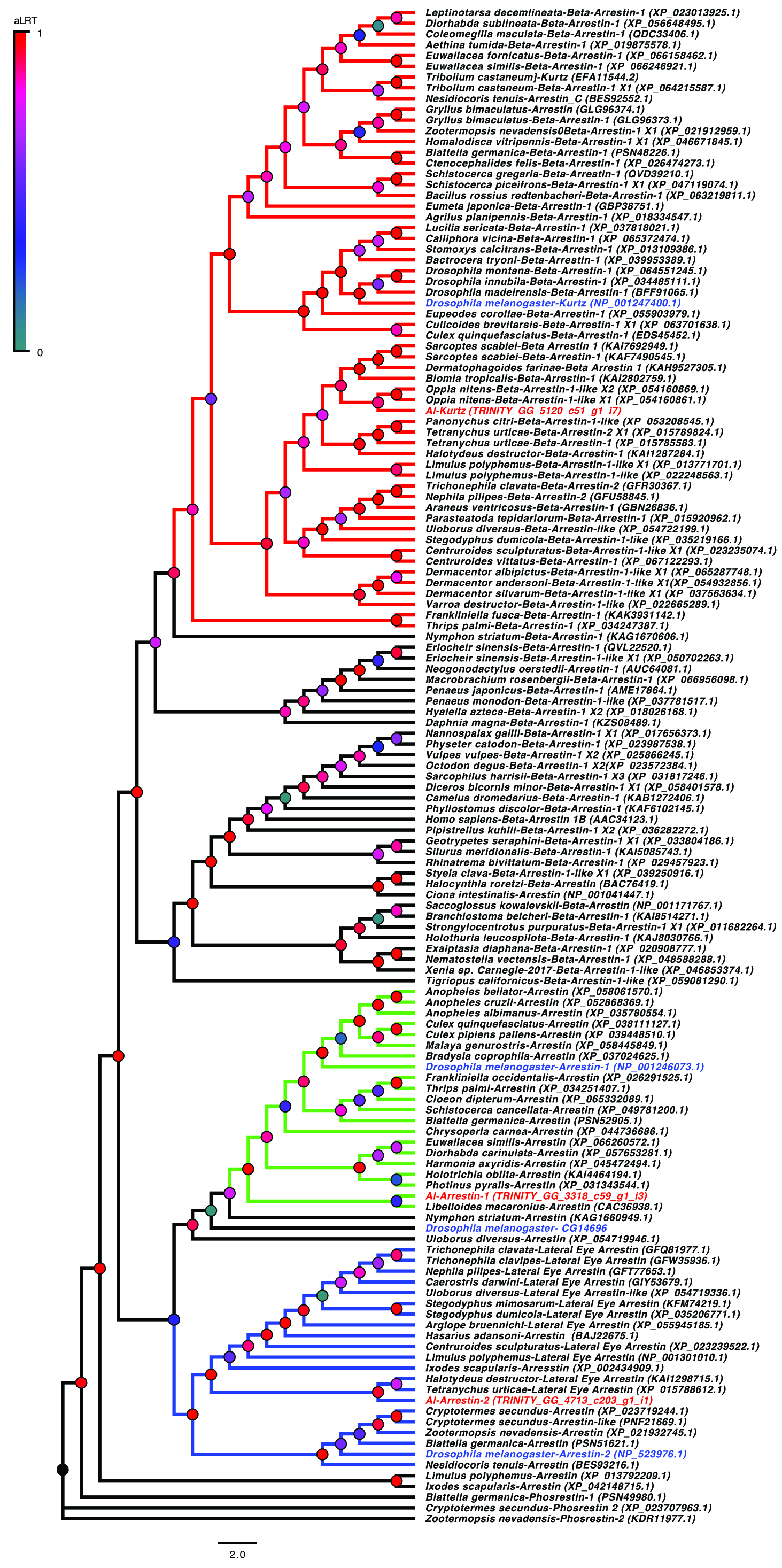

Supplement: Supplementary file 17 — Additional file 17. [file 13227_2025_245_MOESM17_ESM.tif]
