# Supplementary material for: The expression of Pax6 and retinal determination genes in the eyeless arachnid A. longisetosus reveals vestigial eye primordia
Source: EvoDevo. 2025 Jul 9;16:12. doi: 10.1186/s13227-025-00245-7 (PMC12239259; doi:10.1186/s13227-025-00245-7)
Supplement: Supplementary file 2 — Additional file 2. [file 13227_2025_245_MOESM2_ESM.docx]

**Supplementary Text**

**The identification and phylogenetic assessment of the *A. longisetosus* *Pax6* orthologs**

We searched the recently published *A. longisetosus* genome and transcriptome [1] for putative *Pax6* orthologs using tBLASTn [2] and *D. melanogaster* Pax6 protein sequences as queries. This approach allowed us to identify two *Al-ey* transcripts that we refer to as *Al-ey.1* (TRINITY_GG_2648_c164_g1_i2) and *Al-ey.2* (TRINITY_GG_2648_c164_g1_i1), respectively (Fig. S1A). Both transcripts mapped to the same genomic locus (tig00005243_pilon), suggesting that they are isoforms rather than paralogs. The *Al-ey.1* isoform is 2,268 bps long, whereas the slightly longer *Al-ey.2* isoform is 2,354 bps long. Although *Al-ey.2* is longer, this difference is due to a longer 3’UTR. Surprisingly, *Al-ey.2* has a shorter coding sequence in comparison to *Al-ey.1, i.e.*, 670 bps and 1,401 bps, respectively. Therefore, the predicted amino-acid sequence of *Al-ey.1* has a 117 C-terminal extension that *Al-ey.2* lacks. By using Splign [3] to align these transcripts to the genome, we found that the differences between these two isoforms are a result of the differential use of exons towards the 5’ end of the gene. The *Al-ey.1* isoform is spliced from ten exons, whereas *Al-ey.2* is spliced from nine. The 3’ end of *Al-ey.1* is constructed from three exons, *i.e.*, exons 8-10. *Al-ey.2,* however, lacks exons 8 and 9, and instead uses an alternative exon upstream from exon 10. We denoted this exon as “Exon 8.5” due to its position between exons 8 and 9 (Fig. S1A). Using the above methodology, we also identified a single putative *Al-toy* transcript (TRINITY_GG_5245_c530_g1_i1; Fig. S1B). This 1,324 bp transcript is comprised of five exons, and maps to a distinct genomic locus (tig00005236_pilon) from that of the *Al-ey* transcripts.

The deduced protein sequences of the *Al-ey* and *Al-toy* isoforms were then evaluated with the NCBI Conserved Domain Database tool [4]. Both isoforms contain the three characteristic domains found in Pax6 proteins; the paired domain, the octapeptide-like domain, and the homeodomain (Fig. S1A-C) (see [5]). It was recently shown that arthropod Eyeless proteins contain a diagnostic lysine at position 64 in the linker region of the paired domain. This differs from arthropod Toy sequences, which instead have an arginine at this site [6,7]. The amino acid sequences of both *Al-ey* transcripts contain this diagnostic lysine residue (Fig. S1). Furthermore, the deduced Al-Toy amino acid sequence has the characteristic arginine at that site (Fig. S1C). These results support our hypothesis that the retrieved *Pax6* sequences are *de facto* distinct *eyeless* and *toy* orthologs.

To further test the hypotheses that these transcripts represent *bona fide* orthologs of *toy* and *ey*, we performed a maximum-likelihood phylogenetic assessment (PhyML) [8] of the deduced amino acid sequences of these transcripts with those from other metazoan *Pax6* orthologs (Fig. S1D). We also used the amino acid sequence of a putative *Pax2/5/8 A. longisetosus* ortholog (TRINITY_GG_4424_c37_g1_i1) as an outgroup in conjunction with other selected metazoan *Pax2/5/8* orthologs. This phylogenetic interrogation placed *Al-ey* and *Al-toy* in their predicted clades to the exclusion of *Al-Pax2/5* with high support (*i.e.*, aLRT scores of 0.98 for *Al-ey* and 0.96 for *Al-toy;* Fig. S1A). Taken together, our results support the identity of these transcripts as distinct singleton *eyeless* and *toy* orthologs.

**Supplemental References**

1. Brückner A, Barnett AA, Bhat P, Antoshechkin IA, Kitchen SA. Molecular evolutionary trends and biosynthesis pathways in the Oribatida revealed by the genome of Archegozetes longisetosus. Acarologia. 2022;62:532–73.

2. Altschul SF, Gish W, Miller W, Myers EW, Lipman DJ. Basic local alignment search tool. J Mol Biol. 1990;215:403–10.

3. Kapustin Y, Souvorov A, Tatusova T, Lipman D. Splign: Algorithms for computing spliced alignments with identification of paralogs. Biol Direct. 2008;3:1–13.

4. Lu S, Wang J, Chitsaz F, Derbyshire MK, Geer RC, Gonzales NR, et al. CDD/SPARCLE: The conserved domain database in 2020. Nucleic Acids Res. 2020;48:D265–8.

5. Kozmik Z. Pax genes in eye development and evolution. Curr Opin Genet Dev. 2005;15:430–8.

6. Friedrich M. Ancient genetic redundancy of eyeless and twin of eyeless in the arthropod ocular segment. Dev Biol. 2017;432:192–200.

7. Friedrich M. Coming into clear sight at last: Ancestral and derived events during chelicerate visual system development. BioEssays. 2022;44:1–11.

8. Guindon S, Dufayard J, Lefort V. Guindon et al. - 2010 - New Algorithms and Methods to Estimate Maximim-Likelihood Phylogenies Assessing the Performance of PhyML 3.0. Syst Biol. 2010;59:307–21.
