# Supplementary material for: The expression of Pax6 and retinal determination genes in the eyeless arachnid A. longisetosus reveals vestigial eye primordia
Source: EvoDevo. 2025 Jul 9;16:12. doi: 10.1186/s13227-025-00245-7 (PMC12239259; doi:10.1186/s13227-025-00245-7)
Supplement: Supplementary file 3 — Additional file 3. [file 13227_2025_245_MOESM3_ESM.docx]

| Ortholog Search | SMS Substitution Model, BIC | SMS Decoration, BIC | Tree Log-Likelihood |
| --- | --- | --- | --- |
| *eyeless (ey), twin of eyeless (toy)* | Q.insect, 63856.57 | R+F, 131422.34 | -63856.57 |
| *beta-arrestin* | Q.insect, 82423.62 | R+F, 82423.62 | -414303.87 |

**Table S1:** Statistics for the phylogenetic trees. See text for details.
