# Supplementary material for: The expression of Pax6 and retinal determination genes in the eyeless arachnid A. longisetosus reveals vestigial eye primordia
Source: EvoDevo. 2025 Jul 9;16:12. doi: 10.1186/s13227-025-00245-7 (PMC12239259; doi:10.1186/s13227-025-00245-7)
Supplement: Supplementary file 4 — Additional file 4. [file 13227_2025_245_MOESM4_ESM.docx]

| Gene | Transcript | HCR Amplifier |
| --- | --- | --- |
| *Al-arrestin-1* | TRINITY_GG_3318_c59_g1_i3 | B3 |
| *Al-arrestin-2* | TRINITY_GG_4713_c203_g1_i1 | B1 |
| *Al-ato* | TRINITY_GG_4863_c1996_g1_i1 | B2 |
| *Al-dac* | TRINITY_GG_5120_c350_g1_i2 | B2 |
| *Al-ey* | TRINITY_GG_2648_c164_g1_i2 | B1 |
| *Al-eya* | TRINITY_GG_4744_c173_g1_i10 | B2 |
| *Al-krz* | TRINITY_GG_5120_c51_g1_i7 | B2 |
| *Al-otd* | TRINITY_GG_5990_c97_g1_i2 | B3 |
| *Al-peropsin* | TRINITY_GG_4858_c50_g1_i1 | B3 |
| *Al-rhodopsin* | TRINITY_GG_6262_c55_g1_i2 | B3 |
| *Al-Six3* | TRINITY_GG_5990_c859_g1_i1 | B2 |
| *Al-so* | TRINITY_GG_5990_c254_g1_i5 | B3 |
| *Al-toy* | TRINITY_GG_5245_c530_g1_i1 | B1, B2 |
| *Al-wg* | TRINITY_GG_4863_c585_g1_i7 | B2 |

**Table S2:** The transcript IDs and amplifier sequences used to construct each HCR probe. Note that for *Al-toy,* two probe sets were constructed.
