# Supplementary material for: The expression of Pax6 and retinal determination genes in the eyeless arachnid A. longisetosus reveals vestigial eye primordia
Source: EvoDevo. 2025 Jul 9;16:12. doi: 10.1186/s13227-025-00245-7 (PMC12239259; doi:10.1186/s13227-025-00245-7)
Supplement: Supplementary file 5 — Additional file 5. [file 13227_2025_245_MOESM5_ESM.docx]

| *Developmental Landmark* | *A. longisetosus* | *P. tepidariorum* | *C. salei* | *T. pagana* | *P. oplio* |
| --- | --- | --- | --- | --- | --- |
| - Bifurcated ocular lobes are present. | BDS-1 | St. 9.1 | St. 11 | St. 10 | St. 6 |
| - Invaginating Neural Precursors (INPs) first appear - Anterior and lateral furrows appear - Medial "groove" between ocular lobes is present | BDS-2 | St. 10.1   - INPs - Lateral Furrows   St. 11   - Anterior Furrows | St. 11   - INPs   St. 12   - Lateral Furrows   St. 14   - Anterior Furrows | St. 9.2   - INPs   St. 10.2   - Lateral Furrows - Anterior Furrows | St. 8   - INPs   St. 10   - Lateral Furrows - Anterior Furrows |
| - Stomodaeum appears | BDS-3 | St. 10.1 | St. 11 | St. 10.1 | St. 8 |
| - Labral halves have fused | BDS-4 | St. 10.2 | St. 15 | St. 11 | St. 9 |
| - Lateral and anterior furrows have formed a continuous tube. - The medial subdivisions appear | BDS-5 | St. 10.2   - Medial subdivisions appear (no continuous tube) | St. 13   - Medial subdivisions appear (no continuous tube) | St. 10.2   - Medial subdivisions appear (no continuous tube) | NA |
| - Lateral furrows close - Medial subdivisions contact the lateral side of the head to close the anterior furrow. | BDS-6 | St. 12 | St. 16 | St. 12 | NA |
| - The prosomal shield has moved to cover the brain. - The compartments of the brain are distinguishable, with the anterior furrows forming the arcuate bodies, the lateral furrows forming the optic vesicles, and the mushroom bodies forming in between these. | BDS-7 | St. 13-14 | St. 18-19 | St. 13-14 | St. 11-13 |

**Table S3: The comparison of the stages of brain development in *A. longisetosus* compared to the spiders *P. tepidariorum*, *C. salei*, and *T. pagana*, and with the harvestman *P. opilio*.** Data for *P. tepidariorum* is from ref. [73], *C. salei* from [72], *T. pagana* from [35], and *P. opilio* from [69].
