# Supplementary material for: The expression of Pax6 and retinal determination genes in the eyeless arachnid A. longisetosus reveals vestigial eye primordia
Source: EvoDevo. 2025 Jul 9;16:12. doi: 10.1186/s13227-025-00245-7 (PMC12239259; doi:10.1186/s13227-025-00245-7)
Supplement: Supplementary file 6 — Additional file 6. [file 13227_2025_245_MOESM6_ESM.docx]

**Table S4:** Probe pairs designed for *Al-arrestin-1* HCRs (B3 initiator)

| Pair | Initiator | Spacer | Hybridzation | Hybridzation | Spacer | Initiator |
| --- | --- | --- | --- | --- | --- | --- |
| 1 | GTCCCTGCCTCTATATCT | TT | TTTAATAGACTTGAGTTAGCATTGC | AAGCTATGAAAAAGTTGTCGGACGA | TT | CCACTCAACTTTAACCCG |
| 2 | GTCCCTGCCTCTATATCT | TT | TCACTAGTAAGGGAAGAGTTACCTC | ACATTATTGGAGAGGGTGTCGTACG | TT | CCACTCAACTTTAACCCG |
| 3 | GTCCCTGCCTCTATATCT | TT | TGGAAAAGTGATGTGTAGGGTTAGA | AAAAATAGGAATGTCTTGTATGACC | TT | CCACTCAACTTTAACCCG |
| 4 | GTCCCTGCCTCTATATCT | TT | TAGTGATGGAACATTACAGCGACGA | AATGATAGCGACATATCCCGTTCGG | TT | CCACTCAACTTTAACCCG |
| 5 | GTCCCTGCCTCTATATCT | TT | ATCCGTTCAGCTCTTCAACACTGTC | CTACTTAAGTATGATATTTTCACTG | TT | CCACTCAACTTTAACCCG |
| 6 | GTCCCTGCCTCTATATCT | TT | TCACCAAATTTGAACGGAAAAGTCC | TTGGTTTATGTCTCAATTGTGATGC | TT | CCACTCAACTTTAACCCG |
| 7 | GTCCCTGCCTCTATATCT | TT | AGTATAGAGTCAGGTATAGCGTAGA | AGTGACAGACAGCCTAAAGAGTAAA | TT | CCACTCAACTTTAACCCG |
| 8 | GTCCCTGCCTCTATATCT | TT | ACAGAGGCATTGGTGGAAAGTTTGT | CATGTCAGCTACCAGTTACCACCGA | TT | CCACTCAACTTTAACCCG |
| 9 | GTCCCTGCCTCTATATCT | TT | CAACCGTTTACAGCGCTATTAAAAC | TCAGGATTTTTACAGCAAAAGCTCA | TT | CCACTCAACTTTAACCCG |
| 10 | GTCCCTGCCTCTATATCT | TT | CTCATCCAGCCCTTAATGGTTCGAC | CCTTTAAATTGAATACTTCAGACTC | TT | CCACTCAACTTTAACCCG |
| 11 | GTCCCTGCCTCTATATCT | TT | ATTTATGAGACCAAAGACCAAGTGC | CCAAACAAACAAAACGATAAAAAGG | TT | CCACTCAACTTTAACCCG |
| 12 | GTCCCTGCCTCTATATCT | TT | GCTAATCAACAAAATTATAACCAGC | CCATTACAACCAAAGTTACGAACCA | TT | CCACTCAACTTTAACCCG |
| 13 | GTCCCTGCCTCTATATCT | TT | AGACGAGCGCACAAACCATCAGACC | CATTGAATACGGAAGCAGTTTGAGT | TT | CCACTCAACTTTAACCCG |
| 14 | GTCCCTGCCTCTATATCT | TT | AAATAGTGATTCTCCATCACAGACA | GAGAATATTTGCAGAACAATCACTT | TT | CCACTCAACTTTAACCCG |
| 15 | GTCCCTGCCTCTATATCT | TT | GAAAGAAATCGAAACCAATGGTTGG | GACAATTTCGATTATGAGTCAACTC | TT | CCACTCAACTTTAACCCG |
| 16 | GTCCCTGCCTCTATATCT | TT | AAAGTTGATTTGAGAAACTTGCAAG | TCGCATTGACTTTGGGACTGCAGCT | TT | CCACTCAACTTTAACCCG |
| 17 | GTCCCTGCCTCTATATCT | TT | CTTTGAGACATCGATAAAATGGAAT | GGTCCGACAGATTTAGATTTCTCTT | TT | CCACTCAACTTTAACCCG |
| 18 | GTCCCTGCCTCTATATCT | TT | TGAATATACGGCTCATATTTGGGTC | TTACTTTTGTTAGTTCGTCATCTGC | TT | CCACTCAACTTTAACCCG |
| 19 | GTCCCTGCCTCTATATCT | TT | TCTTCTGGTTTTCCACCTTGAAGTA | TTGATCAGGCGATTGGCCGCCTCAA | TT | CCACTCAACTTTAACCCG |
| 20 | GTCCCTGCCTCTATATCT | TT | GCTTGTAATGATTCTCTTTCCTGAG | TTGATCAACTTTTCCAGAACCATTA | TT | CCACTCAACTTTAACCCG |
