# Supplementary material for: The expression of Pax6 and retinal determination genes in the eyeless arachnid A. longisetosus reveals vestigial eye primordia
Source: EvoDevo. 2025 Jul 9;16:12. doi: 10.1186/s13227-025-00245-7 (PMC12239259; doi:10.1186/s13227-025-00245-7)
Supplement: Supplementary file 7 — Additional file 7. [file 13227_2025_245_MOESM7_ESM.docx]

**Table S5:** Probe pairs designed for *Al-arrestin-2* HCRs (B1 initiator)

| Pair | Initiator | Spacer | Hybridzation | Hybridzation | Spacer | Initiator |
| --- | --- | --- | --- | --- | --- | --- |
| 1 | GAGGAGGGCAGCAAACGG | AA | CATAAGAGATAACAATACCAATTGC | CACCGACGTATAGCCTGACGCGAAT | TA | GAAGAGTCTTCCTTTACG |
| 2 | GAGGAGGGCAGCAAACGG | AA | TAGATCAGTATCTGTTTCTCTGAGC | ATCAGATTGTGAGAATGTGGTTGAA | TA | GAAGAGTCTTCCTTTACG |
| 3 | GAGGAGGGCAGCAAACGG | AA | TTGTTTTGTTGTGCCGATGGGATCA | CCGTTTAATGCAATCCCTCTTTTGT | TA | GAAGAGTCTTCCTTTACG |
| 4 | GAGGAGGGCAGCAAACGG | AA | CATTTACAGGGTATCCATCACGAGA | GATAGACTTTAGAGAGTGATGCGCC | TA | GAAGAGTCTTCCTTTACG |
| 5 | GAGGAGGGCAGCAAACGG | AA | CTTCTGTTCGTTGAATGGCGGAAAC | TACTATAATGACCACTTATGAAATT | TA | GAAGAGTCTTCCTTTACG |
| 6 | GAGGAGGGCAGCAAACGG | AA | GCCGTGAAAGTATAGTTCTCGATCG | TATTATAACGTTAACCGATATGACA | TA | GAAGAGTCTTCCTTTACG |
| 7 | GAGGAGGGCAGCAAACGG | AA | CTGAACATAAAACTCTTACTGATCG | CTCACTTCAAGATTCAATTTGCCGG | TA | GAAGAGTCTTCCTTTACG |
| 8 | GAGGAGGGCAGCAAACGG | AA | CTAATAGCCATTGAAACTGAGTCGG | ATTGATTGCTTAACGAACTGTAGTT | TA | GAAGAGTCTTCCTTTACG |
| 9 | GAGGAGGGCAGCAAACGG | AA | CATATTCTATTCCAAGAGGTGGTCC | CTTTATCAGTTACATACAAGATCAG | TA | GAAGAGTCTTCCTTTACG |
| 10 | GAGGAGGGCAGCAAACGG | AA | AGACGGCGGGGCGTTATGCGGCAAT | ATCTTCAGGACCAGGTTGTATAGTG | TA | GAAGAGTCTTCCTTTACG |
| 11 | GAGGAGGGCAGCAAACGG | AA | AGTTTTTGCACGAGTCGTTCCTGAA | AATGTGAAAGGAATAGCATTGGGAC | TA | GAAGAGTCTTCCTTTACG |
| 12 | GAGGAGGGCAGCAAACGG | AA | ATTTCGTCTTCTTCTCTTCCATATC | AATTGTCGTGAAAAGTTGAGTCCCA | TA | GAAGAGTCTTCCTTTACG |
| 13 | GAGGAGGGCAGCAAACGG | AA | ATCTTTAAGATATTGTTGGTCTACC | GGCTGTGATTTGACCAAATAACTGT | TA | GAAGAGTCTTCCTTTACG |
| 14 | GAGGAGGGCAGCAAACGG | AA | TCTTGATGATCACCAAAATCTCGAT | ACAACTCCATTGAGTGGATCACAAT | TA | GAAGAGTCTTCCTTTACG |
