# Supplementary material for: The expression of Pax6 and retinal determination genes in the eyeless arachnid A. longisetosus reveals vestigial eye primordia
Source: EvoDevo. 2025 Jul 9;16:12. doi: 10.1186/s13227-025-00245-7 (PMC12239259; doi:10.1186/s13227-025-00245-7)
Supplement: Supplementary file 8 — Additional file 8. [file 13227_2025_245_MOESM8_ESM.docx]

| Pair | Initiator | Spacer | Hybridzation | Hybridzation | Spacer | Initiator |
| --- | --- | --- | --- | --- | --- | --- |
| 1 | CCTCGTAAATCCTCATCA | AA | TCCGTTTGTTGTTGTTGCTGTTGCG | TAATTGCAAAACTTTTAATCTACTG | AA | ATCATCCAGTAAACCGCC |
| 2 | CCTCGTAAATCCTCATCA | AA | CTTGTTGGTTTTGAGTGATTTGCTG | GGGGAGTCGGTGACGATTTCAGAGA | AA | ATCATCCAGTAAACCGCC |
| 3 | CCTCGTAAATCCTCATCA | AA | CTGCTGCTGATGTATCTCGTGCTCC | ATTCTGTGTATTGTGTGATATCTGT | AA | ATCATCCAGTAAACCGCC |
| 4 | CCTCGTAAATCCTCATCA | AA | CAAATGCTCTCTCATTTCTCGCTCC | TTTTTGTTCTTCATTCAGTTGTTTT | AA | ATCATCCAGTAAACCGCC |
| 5 | CCTCGTAAATCCTCATCA | AA | AGTTCAGCTTTTTGCAAATGCAATT | TCAAGTTGTGCCGCCAACTCAGCCC | AA | ATCATCCAGTAAACCGCC |
| 6 | CCTCGTAAATCCTCATCA | AA | CAATTGCCAAAAGCCCCTCAATATT | TTTGTTGTTGTCTAGCATTATGAGC | AA | ATCATCCAGTAAACCGCC |
| 7 | CCTCGTAAATCCTCATCA | AA | TTGTTGAGTTTGCTGTTGCTGTGGA | CAGCAATGTTTCCAAACAATGTTCC | AA | ATCATCCAGTAAACCGCC |
| 8 | CCTCGTAAATCCTCATCA | AA | TTGCGGTTTCACTCGCACCCCGTGT | GAGGAGTTGTCGAACTGTTTCGATT | AA | ATCATCCAGTAAACCGCC |
| 9 | CCTCGTAAATCCTCATCA | AA | GCTGACAGGGTTGTCGCTATTGTTA | ACACGATATACCGTTAAATCCAGAA | AA | ATCATCCAGTAAACCGCC |
| 10 | CCTCGTAAATCCTCATCA | AA | TCGTCGTCATCATCAGTAGTGTCAT | CTATTATTACTTTCCACGTCGTCTT | AA | ATCATCCAGTAAACCGCC |
| 11 | CCTCGTAAATCCTCATCA | AA | TGCGCTGCTGGTGTTACTGCTGCTG | ATTATTATTATTGTTTGTATTGTTG | AA | ATCATCCAGTAAACCGCC |
| 12 | CCTCGTAAATCCTCATCA | AA | GTTACGAGAGCTTAAGTTAAGTGCG | GTTATTTCCACTACTATTGACATTA | AA | ATCATCCAGTAAACCGCC |
| 13 | CCTCGTAAATCCTCATCA | AA | TGTTGCTGTGAAAGTAACCATAAAT | GAGTTCGGGACATGTTGTTGTTGCT | AA | ATCATCCAGTAAACCGCC |
| 14 | CCTCGTAAATCCTCATCA | AA | CCAGAACCTTCCGATGTGGCGTTGT | GTTGGATTGAAATCTGAGGCCACGG | AA | ATCATCCAGTAAACCGCC |
| 15 | CCTCGTAAATCCTCATCA | AA | CTGGAGGCGGACTTGCATTTGAAAA | CATTGCTTCCGTGATGCGAAGCTAT | AA | ATCATCCAGTAAACCGCC |
| 16 | CCTCGTAAATCCTCATCA | AA | TGGTGCGGTTCCAGCGCTGAAGTGT | CACAAGTTGTTGCTGTTGCTGAGGA | AA | ATCATCCAGTAAACCGCC |
| 17 | CCTCGTAAATCCTCATCA | AA | TTCGCTGCTAGCATTGCTGCTGCGG | AAGAAAGGAAATGCGGCCGAATAAC | AA | ATCATCCAGTAAACCGCC |
| 18 | CCTCGTAAATCCTCATCA | AA | TATTAAGACTGCTATGCGTTTGCTT | CGGCACTTGTGTTACCACTACTATT | AA | ATCATCCAGTAAACCGCC |
| 19 | CCTCGTAAATCCTCATCA | AA | ATCATCAGAGAAACGGGGCTTTTTA | GTCTATATTTTCACTGGCATAGTCA | AA | ATCATCCAGTAAACCGCC |
| 20 | CCTCGTAAATCCTCATCA | AA | TTGTGTTGCTGTGGCCCTGAGGTGA | GTTGGGTATCCGAGATAGGGCACAA | AA | ATCATCCAGTAAACCGCC |
| 21 | CCTCGTAAATCCTCATCA | AA | TGACTGAACTTCTCTTGGGTGGACG | ATGATGGGACTGGGCTAACAATGCC | AA | ATCATCCAGTAAACCGCC |

**Table S7:** Probe pairs designed for *Al-dachshund* HCRs (B2 initiator)
