# Supplementary material for: The expression of Pax6 and retinal determination genes in the eyeless arachnid A. longisetosus reveals vestigial eye primordia
Source: EvoDevo. 2025 Jul 9;16:12. doi: 10.1186/s13227-025-00245-7 (PMC12239259; doi:10.1186/s13227-025-00245-7)
Supplement: Supplementary file 9 — Additional file 9. [file 13227_2025_245_MOESM9_ESM.docx]

**Table S10:** Probe pairs designed for *Al-krz* HCRs (B2 initiator)

| Pair | Initiator | Spacer | Hybridzation | Hybridzation | Spacer | Initiator |
| --- | --- | --- | --- | --- | --- | --- |
| 1 | CCTCGTAAATCCTCATCA | AA | TTGGGCTTTTCTTGTTGGGCTTTTA | TGGGCTTTTCTTGTTGGGCTTTTCT | AA | ATCATCCAGTAAACCGCC |
| 2 | CCTCGTAAATCCTCATCA | AA | GGTATCCTCACATAGAACAGCTCTC | TTAAATACAGATATACCTAAAGACA | AA | ATCATCCAGTAAACCGCC |
| 3 | CCTCGTAAATCCTCATCA | AA | CAGTCTCTCCTCTTAGTCTTAGTCG | GGATTCAAAATTCAATACTTTATGC | AA | ATCATCCAGTAAACCGCC |
| 4 | CCTCGTAAATCCTCATCA | AA | TCTCTTCGGGTTTAGGATGCATCAG | CTACTTTTGAGGCATTTATGTTATT | AA | ATCATCCAGTAAACCGCC |
| 5 | CCTCGTAAATCCTCATCA | AA | CTTCATGTTTAAGTTGACCGTCAAG | CAATTGTTGAAGACGCTAAGTTTGT | AA | ATCATCCAGTAAACCGCC |
| 6 | CCTCGTAAATCCTCATCA | AA | TGTACATTAACTGCAATATCTTCTC | ACAGTTCGGTTCGAATTATTAGCGA | AA | ATCATCCAGTAAACCGCC |
| 7 | CCTCGTAAATCCTCATCA | AA | CTGAGTTTCTTTTATGAGGTTTTTC | AAACCACTTTCCTGATCGCTAATCG | AA | ATCATCCAGTAAACCGCC |
| 8 | CCTCGTAAATCCTCATCA | AA | CTAATTTCTTTATCAGCCTTTCTTG | CAAAGAAGAACGGAAAAGCATTGGA | AA | ATCATCCAGTAAACCGCC |
| 9 | CCTCGTAAATCCTCATCA | AA | AATCTGGATCTATCAGTACAACGCC | GACCAAATACTTTACGATCTTTTAT | AA | ATCATCCAGTAAACCGCC |
| 10 | CCTCGTAAATCCTCATCA | AA | TATGAAACGTTTCGTTCAAACTGTC | TCTGACATTAAGAGAAGCTATGCTT | AA | ATCATCCAGTAAACCGCC |
| 11 | CCTCGTAAATCCTCATCA | AA | TATGGCTTAATCACCAGTCAAATCG | CTAACTTATCCATCACTTCTTTCTT | AA | ATCATCCAGTAAACCGCC |
| 12 | CCTCGTAAATCCTCATCA | AA | TAAAACTGGAACTCAAGTCGAAGGC | TTTTATGCCACGAGAATGCATTTTA | AA | ATCATCCAGTAAACCGCC |
| 13 | CCTCGTAAATCCTCATCA | AA | TAGTCCAAGACATCATTTACGAGAA | GGCCTTAGAACAGTCGCGACTCATT | AA | ATCATCCAGTAAACCGCC |
| 14 | CCTCGTAAATCCTCATCA | AA | TCCCTCTATCCGTATTACAACTTAC | CCGTACATGACATCGCGTGTGAACA | AA | ATCATCCAGTAAACCGCC |
| 15 | CCTCGTAAATCCTCATCA | AA | AATTTGACTCACGTCCTGAATACTC | CAGCCACAAACACGGTTTTGGTGGT | AA | ATCATCCAGTAAACCGCC |
| 16 | CCTCGTAAATCCTCATCA | AA | AAGAATTGCATTAAAGCAACGATCG | GCTGTTAGTAAAAAGTGAATGATAG | AA | ATCATCCAGTAAACCGCC |
| 17 | CCTCGTAAATCCTCATCA | AA | GGCAGTGCTGCCGCTACTCTTCTGA | CTTGCTTTGTCATTGGTCTCCATTC | AA | ATCATCCAGTAAACCGCC |
| 18 | CCTCGTAAATCCTCATCA | AA | GGCATTTGTTATGTCATATACAACC | CCATTTGGACGTTTGATGGAATGAA | AA | ATCATCCAGTAAACCGCC |
| 19 | CCTCGTAAATCCTCATCA | AA | CTGTTCTGTCTTCTAAATACATAGT | CTGCAGTGTCCCATAATTGTAATCG | AA | ATCATCCAGTAAACCGCC |
| 20 | CCTCGTAAATCCTCATCA | AA | CAACACTTTGTTCGCCCAGAAATAC | TAAAACGAGTAATTAATGACGTCTT | AA | ATCATCCAGTAAACCGCC |
