# Supplementary material for: The expression of Pax6 and retinal determination genes in the eyeless arachnid A. longisetosus reveals vestigial eye primordia
Source: EvoDevo. 2025 Jul 9;16:12. doi: 10.1186/s13227-025-00245-7 (PMC12239259; doi:10.1186/s13227-025-00245-7)
Supplement: Supplementary file 10 — Additional file 10. [file 13227_2025_245_MOESM10_ESM.docx]

**Table S11:** Probe pairs designed for *Al-otd* HCRs (B3 initiator).

| Pair | Initiator | Spacer | Hybridzation | Hybridzation | Spacer | Initiator |
| --- | --- | --- | --- | --- | --- | --- |
| 1 | GTCCCTGCCTCTATATCT | TT | TGATAAGAGTGCATTTTGTCATTAG | CGAATATGATCGGGATGTCATGAAA | TT | CCACTCAACTTTAACCCG |
| 2 | GTCCCTGCCTCTATATCT | TT | GGACTTATTATCCGGATTATATTCC | ATTTCACAACACTTGAAACTTCCAG | TT | CCACTCAACTTTAACCCG |
| 3 | GTCCCTGCCTCTATATCT | TT | TTAATTGGTGGTGTCCTAGCGGTCA | CAGTCGTTAGGCGCAGAGATTCCTC | TT | CCACTCAACTTTAACCCG |
| 4 | GTCCCTGCCTCTATATCT | TT | CATGTGTAGACATTACAGGATTCAT | TCTGATGGCCAGTAGGAGCATGTAA | TT | CCACTCAACTTTAACCCG |
| 5 | GTCCCTGCCTCTATATCT | TT | ATTGAGAGCAGATGAGCCCATAGTG | TGAGCTCATAGTTGGGCCACTCATT | TT | CCACTCAACTTTAACCCG |
| 6 | GTCCCTGCCTCTATATCT | TT | ATTGTCGGCATATGATAATCCATAT | ACAGCACCTAACTGTGTGTGATGAG | TT | CCACTCAACTTTAACCCG |
| 7 | GTCCCTGCCTCTATATCT | TT | AACTTTGAGGTGGATAGCAAGAGGC | CATAATGATATGCTGATGCAGGTCC | TT | CCACTCAACTTTAACCCG |
| 8 | GTCCCTGCCTCTATATCT | TT | GTAGGCAGCAGTTGCCCTCTGCATA | AGCACTGCTTGCCATTTGGTGGTGA | TT | CCACTCAACTTTAACCCG |
| 9 | GTCCCTGCCTCTATATCT | TT | GGAGCGATAGCAGCGGGACTCCATA | CTATTACCAGACATCAGATCCGATA | TT | CCACTCAACTTTAACCCG |
| 10 | GTCCCTGCCTCTATATCT | TT | TAACTGAAGAACATAATGGATTCCC | ATGTGTTGGCACTTGAATTGTTTGA | TT | CCACTCAACTTTAACCCG |
| 11 | GTCCCTGCCTCTATATCT | TT | TGACGGCGGCTTGTATGGAGAATCT | GTTACCGCTCGATGTTATGTTTGGC | TT | CCACTCAACTTTAACCCG |
| 12 | GTCCCTGCCTCTATATCT | TT | TTTGTGGCACTACTAGTCCCGTTTT | GGACTCTTGGCCTTCTTCGGCCTCG | TT | CCACTCAACTTTAACCCG |
| 13 | GTCCCTGCCTCTATATCT | TT | CTCTTCTATTTTTAAACCAAACCTG | GTTGTTGGGCCTGCTGGCGACACTT | TT | CCACTCAACTTTAACCCG |
| 14 | GTCCCTGCCTCTATATCT | TT | TTTTGCTGAACAATGCTTCCAAGAC | CTCTCATAAATATATCAGGATATCT | TT | CCACTCAACTTTAACCCG |
| 15 | GTCCCTGCCTCTATATCT | TT | TTCTCTTCTTTGTTTTCTTGGCGGC | GAGTTGTGCTCTGGTGAAAGTAGTC | TT | CCACTCAACTTTAACCCG |
| 16 | GTCCCTGCCTCTATATCT | TT | AAAAACATATCAGGATATCCAACAC | CTCGGACCGTTCGGCGAAATGGCCC | TT | CCACTCAACTTTAACCCG |
| 17 | GTCCCTGCCTCTATATCT | TT | TGTGATGCCTGCGATCCCATTCATT | ATGCAAGAGATCGACGGCACCGGGT | TT | CCACTCAACTTTAACCCG |
| 18 | GTCCCTGCCTCTATATCT | TT | GGACGTTGGCTGAAAAGCCAGTGTT | GCCAGAAGCGAATGGATTGCAAGAC | TT | CCACTCAACTTTAACCCG |
| 19 | GTCCCTGCCTCTATATCT | TT | GATGATGGCGGAGACGAAGGCGATG | ACTCCTGATGACGATATGTTTCTGA | TT | CCACTCAACTTTAACCCG |
| 20 | GTCCCTGCCTCTATATCT | TT | AAGTACCTCCCGTTGGAGTCGAATT | TATTGCACCCACTCAGAGATGTTGT | TT | CCACTCAACTTTAACCCG |
| 21 | GTCCCTGCCTCTATATCT | TT | TTCCTCTTCGGATACTGAGGAGTAC | CCCAGGACTACCGACTTGATGCAGC | TT | CCACTCAACTTTAACCCG |
| 22 | GTCCCTGCCTCTATATCT | TT | TTGTTGGATGTGTTGTGTTTTGATG | TGATGTGCGGATAATAGTTGTGATG | TT | CCACTCAACTTTAACCCG |
| 23 | GTCCCTGCCTCTATATCT | TT | CGTTGCGGAGATATTGCAGGAAAAA | GGAATCCGTGTAATGACCGAATCGT | TT | CCACTCAACTTTAACCCG |
| 24 | GTCCCTGCCTCTATATCT | TT | GTTAACTTCTAGCATGTTTTTGCCT | CGACAACGTTGAGCTGTATAACTGT | TT | CCACTCAACTTTAACCCG |
| 25 | GTCCCTGCCTCTATATCT | TT | CTATCTCTGTCTCTAAAACTTATGC | AGAGATGTGCACTTATAGAGATGGT | TT | CCACTCAACTTTAACCCG |
| 26 | GTCCCTGCCTCTATATCT | TT | GACAATTAAGCTTTGATTATTCGGC | TCAACTCACAGACAAAACACTCGTG | TT | CCACTCAACTTTAACCCG |
| 27 | GTCCCTGCCTCTATATCT | TT | TTGAGCTTTGGTTTTGGTCACATTG | TCAAAGTTTAGTTCAACAGTTGAGA | TT | CCACTCAACTTTAACCCG |
| 28 | GTCCCTGCCTCTATATCT | TT | TCTGACGAATCCATCATTAATTGAC | ACTTACCCGTCAGTATTTGAATCAG | TT | CCACTCAACTTTAACCCG |
| 29 | GTCCCTGCCTCTATATCT | TT | CTATTTCGGAGATAACATTCGCAGG | CGATTGAATGACAATCAAAATTATT | TT | CCACTCAACTTTAACCCG |
| 30 | GTCCCTGCCTCTATATCT | TT | TGTTCACATCAGTTAACTAGAGATC | ACTTTGACTGTGCTTTCAAACAGTC | TT | CCACTCAACTTTAACCCG |
