# Supplementary material for: The expression of Pax6 and retinal determination genes in the eyeless arachnid A. longisetosus reveals vestigial eye primordia
Source: EvoDevo. 2025 Jul 9;16:12. doi: 10.1186/s13227-025-00245-7 (PMC12239259; doi:10.1186/s13227-025-00245-7)
Supplement: Supplementary file 11 — Additional file 11. [file 13227_2025_245_MOESM11_ESM.docx]

**Table S12:** Probe pairs designed for *Al-peropsin* (B3 initiator).

| Pair | Initiator | Spacer | Hybridzation | Hybridzation | Spacer | Initiator |
| --- | --- | --- | --- | --- | --- | --- |
| 1 | GTCCCTGCCTCTATATCT | TT | TCTCTAAACTGTTGGCTTATCCGAC | ATTTCACTTTCGATTTTCTGCTAAT | TT | CCACTCAACTTTAACCCG |
| 2 | GTCCCTGCCTCTATATCT | TT | AACATCCACAAAGCGAACAACTCTT | TTGAGTTAAGCTACTCTGGTCTTTG | TT | CCACTCAACTTTAACCCG |
| 3 | GTCCCTGCCTCTATATCT | TT | AAGTCGATTATTGGTTAGGTAGTAG | AAAAACGGATGATAAGAAAGCTCCT | TT | CCACTCAACTTTAACCCG |
| 4 | GTCCCTGCCTCTATATCT | TT | GCAAATAGTGGCGGAACCAAAGTGA | ATTGGATTAAACAGTGTAGAGGTTT | TT | CCACTCAACTTTAACCCG |
| 5 | GTCCCTGCCTCTATATCT | TT | ATACTGTCCACAAGCAGAGTATCGC | AAAGAGGAACTGTTTTAGGATCACC | TT | CCACTCAACTTTAACCCG |
| 6 | GTCCCTGCCTCTATATCT | TT | AACTATGATAGTCGACATTATAGTT | GGGAGACCACGAAAACACAAATACA | TT | CCACTCAACTTTAACCCG |
| 7 | GTCCCTGCCTCTATATCT | TT | ATTAATTCCAGCGTTTCCTCGCTTA | TTTTTCCCTAACCCAAATGTCTTCT | TT | CCACTCAACTTTAACCCG |
| 8 | GTCCCTGCCTCTATATCT | TT | GCATAATAGCAGTAGAAAATAACGC | GAACCATTCCTGACTTTACTAACAA | TT | CCACTCAACTTTAACCCG |
| 9 | GTCCCTGCCTCTATATCT | TT | TTATGTCTCCAATCTATAGTACACG | ATTATGAATGATTTATAAGCCGCGT | TT | CCACTCAACTTTAACCCG |
| 10 | GTCCCTGCCTCTATATCT | TT | CCCAACCTATTAATGGCATTAATGC | TTATACTTGAATCCAAGCCATATCT | TT | CCACTCAACTTTAACCCG |
| 11 | GTCCCTGCCTCTATATCT | TT | ATTTGCAGTAATTGTATTTATCCCG | AAGAGTACAACCAAACAGCGTTTAT | TT | CCACTCAACTTTAACCCG |
| 12 | GTCCCTGCCTCTATATCT | TT | CAAAGCCAGTAATGTTAAAGTGCCG | CTTTTGGCAAGAAATATTATATCTG | TT | CCACTCAACTTTAACCCG |
| 13 | GTCCCTGCCTCTATATCT | TT | AAAGCATATGCTTGACAGCCATCAT | TGTGCAGAACCTGCCAGAAACCCCA | TT | CCACTCAACTTTAACCCG |
