# Supplementary material for: The expression of Pax6 and retinal determination genes in the eyeless arachnid A. longisetosus reveals vestigial eye primordia
Source: EvoDevo. 2025 Jul 9;16:12. doi: 10.1186/s13227-025-00245-7 (PMC12239259; doi:10.1186/s13227-025-00245-7)
Supplement: Supplementary file 12 — Additional file 12. [file 13227_2025_245_MOESM12_ESM.docx]

**Table S13:** Probe pairs designed for *Al-rhodopsin* HCRs (B3 initiator)

| Pair | Initiator | Spacer | Hybridzation | Hybridzation | Spacer | Initiator |
| --- | --- | --- | --- | --- | --- | --- |
| 1 | GTCCCTGCCTCTATATCT | TT | GTTTTATTAAAATGGGTAAACTTTA | CCTACACGACGCTCTTCCGATCTCA | TT | CCACTCAACTTTAACCCG |
| 2 | GTCCCTGCCTCTATATCT | TT | CTGATTGTTGTTAACCTCAGATTCG | TTTAACTCATAAAAGTGTGTGTTGT | TT | CCACTCAACTTTAACCCG |
| 3 | GTCCCTGCCTCTATATCT | TT | AGCCAACATATGACACGAATAGAAC | AAACGGATTATAGCAGATTGAACTC | TT | CCACTCAACTTTAACCCG |
| 4 | GTCCCTGCCTCTATATCT | TT | AATATGAAAGCAATTCAAAGGCAAC | GATGACTTCTGGACTAAAATCGGCA | TT | CCACTCAACTTTAACCCG |
| 5 | GTCCCTGCCTCTATATCT | TT | CTTTTCTGGATATCATTCTCTCCCG | ATAATCTTTCGATCAGATCTTTTGA | TT | CCACTCAACTTTAACCCG |
| 6 | GTCCCTGCCTCTATATCT | TT | TCTCACATAAGCGACTGTTGTTATC | ATATTTTCTTCTTATCTGACACCCG | TT | CCACTCAACTTTAACCCG |
| 7 | GTCCCTGCCTCTATATCT | TT | TCTGGATATATAGCTCTGCATCGAG | ATTATTTGTCGAGATCTATTTTGTG | TT | CCACTCAACTTTAACCCG |
| 8 | GTCCCTGCCTCTATATCT | TT | GTATCTATCGATAGCTATTACGGCC | TTGATAAAACCTAGAGTTTATTGCT | TT | CCACTCAACTTTAACCCG |
| 9 | GTCCCTGCCTCTATATCT | TT | ACCTGCATTGAAGGCAATAGTTTGC | GTGAACGTTGAAACATATACAAAAG | TT | CCACTCAACTTTAACCCG |
| 10 | GTCCCTGCCTCTATATCT | TT | GCAATATCCTCGCGATAATGAACGG | AAATCACACCAAAAGGCCAGTTATT | TT | CCACTCAACTTTAACCCG |
| 11 | GTCCCTGCCTCTATATCT | TT | TGGTTTGATCGGAAAAATTGAAACC | ACTTGAGTTTTGCACTGAATCCATC | TT | CCACTCAACTTTAACCCG |
