# Supplementary material for: The expression of Pax6 and retinal determination genes in the eyeless arachnid A. longisetosus reveals vestigial eye primordia
Source: EvoDevo. 2025 Jul 9;16:12. doi: 10.1186/s13227-025-00245-7 (PMC12239259; doi:10.1186/s13227-025-00245-7)
Supplement: Supplementary file 13 — Additional file 13. [file 13227_2025_245_MOESM13_ESM.docx]

**Table S16:** Probe pairs designed for *Al-toy* HCRs (B1 initiator)

| Pair | Initiator | Spacer | Hybridzation | Hybridzation | Spacer | Initiator |
| --- | --- | --- | --- | --- | --- | --- |
| 1 | GAGGAGGGCAGCAAACGG | AA | CGATTGTGGATTTGGTTGAGCAGAC | TATAAAATGATTGGAGTAATTGTGA | TA | GAAGAGTCTTCCTTTACG |
| 2 | GAGGAGGGCAGCAAACGG | AA | AAATAAGGTTGAGATGATTGAGGCG | TCGAACGGATCCACAGAATGGCCCA | TA | GAAGAGTCTTCCTTTACG |
| 3 | GAGGAGGGCAGCAAACGG | AA | TGTTGAAGTTTTGGATTGAATTACT | GCGGAGGAGGCGTTAGGGCCGAGAA | TA | GAAGAGTCTTCCTTTACG |
| 4 | GAGGAGGGCAGCAAACGG | AA | GTTGACGAAATTTCGGTTGTTTTCC | CGATGAGAGTGTGGCCAAAGAGGAA | TA | GAAGAGTCTTCCTTTACG |
| 5 | GAGGAGGGCAGCAAACGG | AA | ATTTGGTTATCGGCAGGAGTGTTAC | TTTGAACTCTTGTTAGTATTGCTAT | TA | GAAGAGTCTTCCTTTACG |
| 6 | GAGGAGGGCAGCAAACGG | AA | TGGCTCTTCTGTTTGAAAACCAAAC | TTCTCAATTTTTCTTCTCGTCTCCA | TA | GAAGAGTCTTCCTTTACG |
| 7 | GAGGAGGGCAGCAAACGG | AA | ATCAGCCAGTTTCTCGCGAGCAAAC | AATTCTAGCTTCCGGTAAACTTATT | TA | GAAGAGTCTTCCTTTACG |
| 8 | GAGGAGGGCAGCAAACGG | AA | TTCGCTGTAATCTTCTTTTGAGTCG | TTTGTTCATCAGTAAAAGCAGTCCT | TA | GAAGAGTCTTCCTTTACG |
| 9 | GAGGAGGGCAGCAAACGG | AA | CCATCTGATGAATAATTATTTTCGG | CTCAATTGCGACTCTTCATCTGTAG | TA | GAAGAGTCTTCCTTTACG |
| 10 | GAGGAGGGCAGCAAACGG | AA | GCCGAAGACAGCCTTCATGAGCACT | TGACATCACAAGTGAGCTTATCTGT | TA | GAAGAGTCTTCCTTTACG |
| 11 | GAGGAGGGCAGCAAACGG | AA | TACCGGAGGGCTACCACTGGATGTT | TGTCACATCTCCACAATTAGTTAGA | TA | GAAGAGTCTTCCTTTACG |
| 12 | GAGGAGGGCAGCAAACGG | AA | GGTGAATGCGGTGATGAGGAAGGGG | CTACCATTATTTTGGCCAAAGTGCT | TA | GAAGAGTCTTCCTTTACG |
| 13 | GAGGAGGGCAGCAAACGG | AA | GATGTGCTGAAATCATGCGAAATTT | ATGATGTGGAATGATACCATTGCCA | TA | GAAGAGTCTTCCTTTACG |
| 14 | GAGGAGGGCAGCAAACGG | AA | TTTCTGTGCGGCCAAATTTCTTAAT | CAAATTATTGCCACTGACTTGTTGT | TA | GAAGAGTCTTCCTTTACG |
| 15 | GAGGAGGGCAGCAAACGG | AA | GTATCGGTGTTGCAAACGTTTTCAG | CTGTTTATTGATGAGACACTTGGAA | TA | GAAGAGTCTTCCTTTACG |
| 16 | GAGGAGGGCAGCAAACGG | AA | AAATGGACGGACACTCGCGTTTGTA | ACAATCTATCACGTATTTCCCACGC | TA | GAAGAGTCTTCCTTTACG |
| 17 | GAGGAGGGCAGCAAACGG | AA | GGCAACACGTGGTTTACTTCCGCCT | AGTAATCGCGTGCACTACTATCGAA | TA | GAAGAGTCTTCCTTTACG |
| 18 | GAGGAGGGCAGCAAACGG | AA | TAATAGCGGCCAAGAATCTTCGAGA | GCTCTTGGTTTGATAGACCCAGTTT | TA | GAAGAGTCTTCCTTTACG |
| 19 | GAGGAGGGCAGCAAACGG | AA | ATATATCACAAGGTCGGGCACCGCT | AGCCGTTCGATATTTGTAGCATTCG | TA | GAAGAGTCTTCCTTTACG |
| 20 | GAGGAGGGCAGCAAACGG | AA | TGAGTCCGGCAGTGGCCGACCGTTC | TGCCAAATCTATGATTTTCTGTCGG | TA | GAAGAGTCTTCCTTTACG |
| 21 | GAGGAGGGCAGCAAACGG | AA | CTTTGACCCTTATGATGCATGTCTG | TAGACTCCACCCAATTGATTGATTC | TA | GAAGAGTCTTCCTTTACG |
