# Supplementary material for: The expression of Pax6 and retinal determination genes in the eyeless arachnid A. longisetosus reveals vestigial eye primordia
Source: EvoDevo. 2025 Jul 9;16:12. doi: 10.1186/s13227-025-00245-7 (PMC12239259; doi:10.1186/s13227-025-00245-7)
Supplement: Supplementary file 14 — Additional file 14. [file 13227_2025_245_MOESM14_ESM.docx]

**Table S17:** Probe pairs designed for *Al-toy* HCRs (B2 initiator).

| Pair | Initiator | Spacer | Hybridzation | Hybridzation | Spacer | Initiator |
| --- | --- | --- | --- | --- | --- | --- |
| 1 | CCTCGTAAATCCTCATCA | AA | CGATTGTGGATTTGGTTGAGCAGAC | TATAAAATGATTGGAGTAATTGTGA | AA | ATCATCCAGTAAACCGCC |
| 2 | CCTCGTAAATCCTCATCA | AA | AAATAAGGTTGAGATGATTGAGGCG | TCGAACGGATCCACAGAATGGCCCA | AA | ATCATCCAGTAAACCGCC |
| 3 | CCTCGTAAATCCTCATCA | AA | TGTTGAAGTTTTGGATTGAATTACT | GCGGAGGAGGCGTTAGGGCCGAGAA | AA | ATCATCCAGTAAACCGCC |
| 4 | CCTCGTAAATCCTCATCA | AA | GTTGACGAAATTTCGGTTGTTTTCC | CGATGAGAGTGTGGCCAAAGAGGAA | AA | ATCATCCAGTAAACCGCC |
| 5 | CCTCGTAAATCCTCATCA | AA | ATTTGGTTATCGGCAGGAGTGTTAC | TTTGAACTCTTGTTAGTATTGCTAT | AA | ATCATCCAGTAAACCGCC |
| 6 | CCTCGTAAATCCTCATCA | AA | TGGCTCTTCTGTTTGAAAACCAAAC | TTCTCAATTTTTCTTCTCGTCTCCA | AA | ATCATCCAGTAAACCGCC |
| 7 | CCTCGTAAATCCTCATCA | AA | ATCAGCCAGTTTCTCGCGAGCAAAC | AATTCTAGCTTCCGGTAAACTTATT | AA | ATCATCCAGTAAACCGCC |
| 8 | CCTCGTAAATCCTCATCA | AA | TTCGCTGTAATCTTCTTTTGAGTCG | TTTGTTCATCAGTAAAAGCAGTCCT | AA | ATCATCCAGTAAACCGCC |
| 9 | CCTCGTAAATCCTCATCA | AA | CCATCTGATGAATAATTATTTTCGG | CTCAATTGCGACTCTTCATCTGTAG | AA | ATCATCCAGTAAACCGCC |
| 10 | CCTCGTAAATCCTCATCA | AA | GCCGAAGACAGCCTTCATGAGCACT | TGACATCACAAGTGAGCTTATCTGT | AA | ATCATCCAGTAAACCGCC |
| 11 | CCTCGTAAATCCTCATCA | AA | TACCGGAGGGCTACCACTGGATGTT | TGTCACATCTCCACAATTAGTTAGA | AA | ATCATCCAGTAAACCGCC |
| 12 | CCTCGTAAATCCTCATCA | AA | GGTGAATGCGGTGATGAGGAAGGGG | CTACCATTATTTTGGCCAAAGTGCT | AA | ATCATCCAGTAAACCGCC |
| 13 | CCTCGTAAATCCTCATCA | AA | GATGTGCTGAAATCATGCGAAATTT | ATGATGTGGAATGATACCATTGCCA | AA | ATCATCCAGTAAACCGCC |
| 14 | CCTCGTAAATCCTCATCA | AA | TTTCTGTGCGGCCAAATTTCTTAAT | CAAATTATTGCCACTGACTTGTTGT | AA | ATCATCCAGTAAACCGCC |
| 15 | CCTCGTAAATCCTCATCA | AA | GTATCGGTGTTGCAAACGTTTTCAG | CTGTTTATTGATGAGACACTTGGAA | AA | ATCATCCAGTAAACCGCC |
| 16 | CCTCGTAAATCCTCATCA | AA | AAATGGACGGACACTCGCGTTTGTA | ACAATCTATCACGTATTTCCCACGC | AA | ATCATCCAGTAAACCGCC |
| 17 | CCTCGTAAATCCTCATCA | AA | GGCAACACGTGGTTTACTTCCGCCT | AGTAATCGCGTGCACTACTATCGAA | AA | ATCATCCAGTAAACCGCC |
| 18 | CCTCGTAAATCCTCATCA | AA | TAATAGCGGCCAAGAATCTTCGAGA | GCTCTTGGTTTGATAGACCCAGTTT | AA | ATCATCCAGTAAACCGCC |
| 19 | CCTCGTAAATCCTCATCA | AA | ATATATCACAAGGTCGGGCACCGCT | AGCCGTTCGATATTTGTAGCATTCG | AA | ATCATCCAGTAAACCGCC |
| 20 | CCTCGTAAATCCTCATCA | AA | TGAGTCCGGCAGTGGCCGACCGTTC | TGCCAAATCTATGATTTTCTGTCGG | AA | ATCATCCAGTAAACCGCC |
| 21 | CCTCGTAAATCCTCATCA | AA | CTTTGACCCTTATGATGCATGTCTG | TAGACTCCACCCAATTGATTGATTC | AA | ATCATCCAGTAAACCGCC |
