# Supplementary material for: The expression of Pax6 and retinal determination genes in the eyeless arachnid A. longisetosus reveals vestigial eye primordia
Source: EvoDevo. 2025 Jul 9;16:12. doi: 10.1186/s13227-025-00245-7 (PMC12239259; doi:10.1186/s13227-025-00245-7)
Supplement: Supplementary file 15 — Additional file 15. [file 13227_2025_245_MOESM15_ESM.docx]

| Pair | Initiator | Spacer | Hybridzation | Hybridzation | Spacer | Initiator |
| --- | --- | --- | --- | --- | --- | --- |
| 1 | CCTCGTAAATCCTCATCA | AA | GATCGGTTTTAACCATTTCAACAGG | AGTGTTTACTCGTTTTCAACACCAT | AA | ATCATCCAGTAAACCGCC |
| 2 | CCTCGTAAATCCTCATCA | AA | GCTTTACAAACCTTGCATTTGACCT | CACAAACACGTATGAACTGTTTTTC | AA | ATCATCCAGTAAACCGCC |
| 3 | CCTCGTAAATCCTCATCA | AA | GCTCTAATTCCTCTCTGATTTCAGT | AACACCAATGAAAAGTACAATTACA | AA | ATCATCCAGTAAACCGCC |
| 4 | CCTCGTAAATCCTCATCA | AA | ACATCCTTCAACACCGATAGAAGTG | ATATCCTCGCCCACAACATAATAAA | AA | ATCATCCAGTAAACCGCC |
| 5 | CCTCGTAAATCCTCATCA | AA | ACTCCAAATTTAGGATTAGGAGTAC | TTACAAACTCTTCCTTTAGTCCCAA | AA | ATCATCCAGTAAACCGCC |
| 6 | CCTCGTAAATCCTCATCA | AA | TGCTGACCATTACCCTCGAAGCGCC | TTTTTCTTGTTAATCCTCTGTAGTC | AA | ATCATCCAGTAAACCGCC |
| 7 | CCTCGTAAATCCTCATCA | AA | GTCTCTGAATGGAGGCAACCGCATC | AAATCTATCTTTAAGATTATTGCCA | AA | ATCATCCAGTAAACCGCC |
| 8 | CCTCGTAAATCCTCATCA | AA | ATTCCATGACATTTGCATTCACGCC | CACGTGCGTACAGTACATGAACCTG | AA | ATCATCCAGTAAACCGCC |
| 9 | CCTCGTAAATCCTCATCA | AA | CTTCATTGTTGTGCAGATTCATTAT | TCTCATTTGAAACATGCGAACGACC | AA | ATCATCCAGTAAACCGCC |
| 10 | CCTCGTAAATCCTCATCA | AA | ATCAACAAAGGCTCGCGCAAACTTA | TCTGAGATCTCGTCCTCTTTCAGCT | AA | ATCATCCAGTAAACCGCC |
| 11 | CCTCGTAAATCCTCATCA | AA | CCCCACTCCCAGTCCAGACCATTTG | CCAAATTCGATATTATCCGAACAGC | AA | ATCATCCAGTAAACCGCC |
| 12 | CCTCGTAAATCCTCATCA | AA | ATGTCTCTATCAAACCTTCGCTGCA | TCCGATTATTACGATAATCACATGT | AA | ATCATCCAGTAAACCGCC |
| 13 | CCTCGTAAATCCTCATCA | AA | ACTCGTTATCGCATAAACGAACGCA | TCTAGAAATTGAATGCGCCACTGCC | AA | ATCATCCAGTAAACCGCC |
| 14 | CCTCGTAAATCCTCATCA | AA | CCAAAAATTCCTTTGCCTTTCATGT | TCTCTGCAACCCCTTTGTACAATTC | AA | ATCATCCAGTAAACCGCC |
| 15 | CCTCGTAAATCCTCATCA | AA | CTTTGAATTGGTTTTGGCATTCGTT | CGGTTGTGGGACAGTTCCATCGTCT | AA | ATCATCCAGTAAACCGCC |
| 16 | CCTCGTAAATCCTCATCA | AA | AATGAGAACTCCAGGATTGTCCCTG | TGCCATTTTCATGGCTTTGGCAACG | AA | ATCATCCAGTAAACCGCC |
| 17 | CCTCGTAAATCCTCATCA | AA | TGTGCCGCCAGTAATTGAATTAGTG | ACGTAAAGGAACATTAATACCGTTA | AA | ATCATCCAGTAAACCGCC |
| 18 | CCTCGTAAATCCTCATCA | AA | CACCCGAGTGTGCGATTGCCCACCA | GCGGGTCTAGTACTAGATTTGTTGG | AA | ATCATCCAGTAAACCGCC |
| 19 | CCTCGTAAATCCTCATCA | AA | TCTTGGCATCAATCTTGACGGGCAT | ATCCCTTTCCCCGACCGCCTTTGCC | AA | ATCATCCAGTAAACCGCC |
| 20 | CCTCGTAAATCCTCATCA | AA | TATAATAGCACAAACACCGACTCTA | AATGATCAGAGACATGATCAGCATC | AA | ATCATCCAGTAAACCGCC |
| 21 | CCTCGTAAATCCTCATCA | AA | TTGTTACATGTCCTCAAACGCATGG | GTCCAAATGGCACTTAGAGGCACCC | AA | ATCATCCAGTAAACCGCC |
| 22 | CCTCGTAAATCCTCATCA | AA | GCCCGATCGGCCGACACGGCCGCAC | AAACAGCAAACACTAATTTGATGTT | AA | ATCATCCAGTAAACCGCC |
| 23 | CCTCGTAAATCCTCATCA | AA | TGAGTCGACACGTGCGCTCTTATCA | AAAAAGTTTCCAAATCTGCGTTCAG | AA | ATCATCCAGTAAACCGCC |
| 24 | CCTCGTAAATCCTCATCA | AA | TCGTATTTCATGCAGAGGCAAGTCC | CTTTGGTCTTGAAATGCCAGAACGC | AA | ATCATCCAGTAAACCGCC |
| 25 | CCTCGTAAATCCTCATCA | AA | TATTTACCAGTGCTTTACTGCTCGA | GTTCTGAGCCCACGCCCGCTTCAAC | AA | ATCATCCAGTAAACCGCC |
| 26 | CCTCGTAAATCCTCATCA | AA | AGAAAAAGTAAAGTTTCACAAACGC | TCAAAACCAGCCAACAAAGAGATGA | AA | ATCATCCAGTAAACCGCC |
| 27 | CCTCGTAAATCCTCATCA | AA | AGACTTTCAGTTGGATAGACTTGGC | TCAATCAACTCTATCTATGAATTGA | AA | ATCATCCAGTAAACCGCC |
| 28 | CCTCGTAAATCCTCATCA | AA | CGATGTCTGGTTAAGAATCAGAACT | TGTCGTTTCTCCGATTCAGAGAAGT | AA | ATCATCCAGTAAACCGCC |
| 29 | CCTCGTAAATCCTCATCA | AA | AGGGACCCAGAATTGGGTGGTCTCC | GAATAAATATTCATTAGTATAATTT | AA | ATCATCCAGTAAACCGCC |
| 30 | CCTCGTAAATCCTCATCA | AA | TTAGATAGTAGACCATTGATTGGAG | AAATAGAAAATTCGCGTCATTGTGA | AA | ATCATCCAGTAAACCGCC |
| 31 | CCTCGTAAATCCTCATCA | AA | TTTGCGAGCCGTGAAGTTTCTAATT | AGGACAAAAGAATGTGTGAACTTAT | AA | ATCATCCAGTAAACCGCC |
| 32 | CCTCGTAAATCCTCATCA | AA | TTCTTAAGTCACGAAATATACCTGA | TCTTTGGACAAGGAACACGCCTTTT | AA | ATCATCCAGTAAACCGCC |
| 33 | CCTCGTAAATCCTCATCA | AA | TTGCTCACACTATATTCCGAAGAGT | TCCTTACGCTATATTCCTGACACTT | AA | ATCATCCAGTAAACCGCC |
| 34 | CCTCGTAAATCCTCATCA | AA | TGCTTATACACTGTTGCTTACACTC | CTGAACATGCTATTGCTTATACTCT | AA | ATCATCCAGTAAACCGCC |
| 35 | CCTCGTAAATCCTCATCA | AA | TTCCTTACACTCTGTTCCTCACACT | TCCTTATACACTATTGCTTGCACTC | AA | ATCATCCAGTAAACCGCC |
| 36 | CCTCGTAAATCCTCATCA | AA | ATCCCTCATACTCTATTGCCAACAC | TTCCTCACACTCTATTCCTTATACT | AA | ATCATCCAGTAAACCGCC |
| 37 | CCTCGTAAATCCTCATCA | AA | CAAATTGAATGAAATTCATTCCTTG | CCCTGAAGGCAGATATTATAGTGGA | AA | ATCATCCAGTAAACCGCC |
| 38 | CCTCGTAAATCCTCATCA | AA | AGGCATAAAAACAACTTAATCCTAT | GTCGTCATTCGTGTTGCGCTATAGA | AA | ATCATCCAGTAAACCGCC |
| 39 | CCTCGTAAATCCTCATCA | AA | GAACCATTTGATGCTTCCCTAACAC | AGCAAAGACTGAACCATGAGATGAA | AA | ATCATCCAGTAAACCGCC |

**Table S18:** Probe pairs designed for *Al-wg* HCRs (B2 initiator).
